# Olefin Metathesis Catalyzed by a Latent Ruthenathiete Complex

**DOI:** 10.1021/acs.organomet.6c00021

**Published:** 2026-03-19

**Authors:** Alec B. Pabarue, Tianqi Zhang, Mizhi Xu, John Bacsa, Will R. Gutekunst

**Affiliations:** † School of Chemistry and Biochemistry, 1372 Georgia Institute of Technology, Atlanta, Georgia 30332, United States; ‡ X-ray Crystallography Center, Department of Chemistry, 1371 Emory University, 1515 Dickey Drive, Atlanta, Georgia 30322, United States

## Abstract

Four-membered chelate complexes of ruthenium are rare, with only a handful of examples present in the literature. The first four-membered sulfur chelate, a *cis*-dichloro ruthenathiete complex, is reported to demonstrate latency toward olefin metathesis reactions until heated or irradiated with UV light. This complex’s reactivity was benchmarked through ring-closing metathesis, cross-metathesis, and ring-opening metathesis polymerization reactions in deuterated chloroform and toluene as solvents. Despite the more strained 4-membered ring, the complex displays similar latency to 5-membered analogs previously reported in the literature. Key H−π interactions were observed between the mesityl methyl groups on the *N*-heterocyclic carbene and the aromatic groups on the alkylidene ligand, which are proposed to play a central role in the stability of the ruthenathiete. Collectively, this introduces a new class of sulfur-chelated ruthenium complexes for use in stimuli-promoted metathesis reactions.

## Graphical abstract

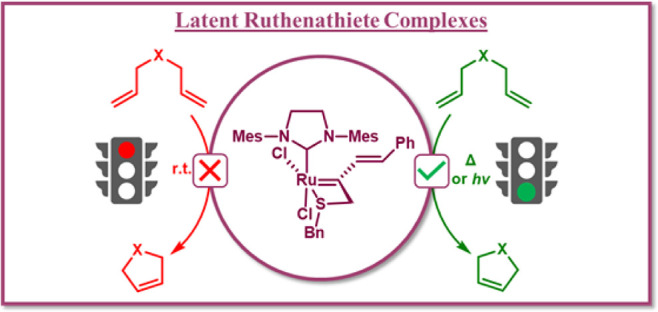

## Introduction

Olefin metathesis is a widely used and efficient method for forming carbon–carbon bonds, with ruthenium-based carbene catalysts commonly used due to their ease of handling and functional group tolerance.^1–4^ While most ruthenium carbene catalysts have been developed for efficient initiation at moderate temperature through the dissociation of pyridine or phosphine ligands upon dissolution, latent catalysts that generate an active 14-electron complex in response to certain stimuli (heat, light, or exposure to acid) have found particular utility in polymer science.^5–16^ For example, acyclic diene metathesis (ADMET) polymerization is a step-growth process for making polyolefins that is often performed in the bulk to prevent the formation of cyclic polymers.^17^ However, for monomers with high melt temperatures, catalysts that slowly activate at high temperatures are critical for achieving high molecular weights.^18^ Latent catalysts have also proven useful in scenarios where precise spatiotemporal control over metathesis is needed, such as 3D-printing materials, degradable thermosets, and patterning crystallinity.^13,19–26^

These stimuli-responsive characteristics are commonly achieved by introducing chelating heteroatoms to the ruthenium alkylidene or benzylidene substituent.^12,13,23–27^ A classic example is the Hoveyda–Grubbs catalyst, which bears a 5-membered oxygen chelate and displays increased stability and recyclability.^28^ However, complete latency at room temperature is achieved by introducing stronger-coordinating atoms such as sulfur or nitrogen. Grubbs and coworkers developed complex **1** which bears a tridentate ligand with both an imine and thioether that showed latency at room temperature and moderate ring-closing metathesis (RCM) conversions (<50%) upon heating to 60 °C for 30 min (Figure 1a).^7^ The Lemcoff group has reported a range of ruthenium complexes with 5-membered sulfur chelates that feature a *cis*-dichloro stereochemistry (**2**). *Cis/trans* isomerization occurs in these complexes upon exposure to a thermal or photochemical stimulus, increasing the lability of the sulfur ligand due to the *trans*-effect.^29–32^ The *cis* complexes are inert at room temperature but activate upon heating above 80 °C and isomerization to the *trans* isomer.^15,29,33,34^ Additionally, Lemcoff showed metathesis reactivity could be further inhibited by increasing the valency of the chelate, with the bis-thioether complex **3** showing lower activity than monothioether complexes.^35^ More recently, additional groups have examined 6-membered sulfur chelates.^30,36,37^ Zubkov reported *cis* 6-membered sulfur chelates (**4**) that were found to have higher catalytic activity than their 5-membered counterparts (**2**). Zubkov and Shcheglova also isolated *trans* 6-membered ruthenium chelates (**5**) which are active at room temperature unlike the corresponding *cis* complexes.^30,37^ Despite the many examples of 5- and 6-membered sulfur chelates to ruthenium, the 4-membered ruthenathietes remain unexplored, and ruthenium complexes bearing an alkylidene that forms a 4-membered chelate to any heteroatom are rare.^38–40^ Roper and coworkers developed a ruthenium complex that featured a bis-pyrrole alkylidene that forms an X-type chelate upon deprotonation of one of the pyrroles (**6**, Figure 1b).^40^ The Caulton group reported ruthenium complex **7** that was synthesized from the double C–H activation of the pincer ligand [(^t^Bu_2_PCH_2_SiMe_2_)_2_N^–^],^38^ though this complex was only stable at −78 °C and rapidly decomposed in the presence of pyridine or carbon monoxide. Finally, Fürstner showed that 4-membered chelate (**9**) forms in situ from the *gem*-hydrogenation of alkynes, but this species was never isolated from a mixture of **8** and **9**.^39^ Homo- and heterobimetallic four-membered rings containing ruthenium have also been reported and proven useful in modulating reactivity metathesis reactions.^16^ Here, we report the first examples of ruthenathiete complexes and benchmark their catalytic activity in olefin metathesis reactions (Figure 1c).

**Figure 1:**
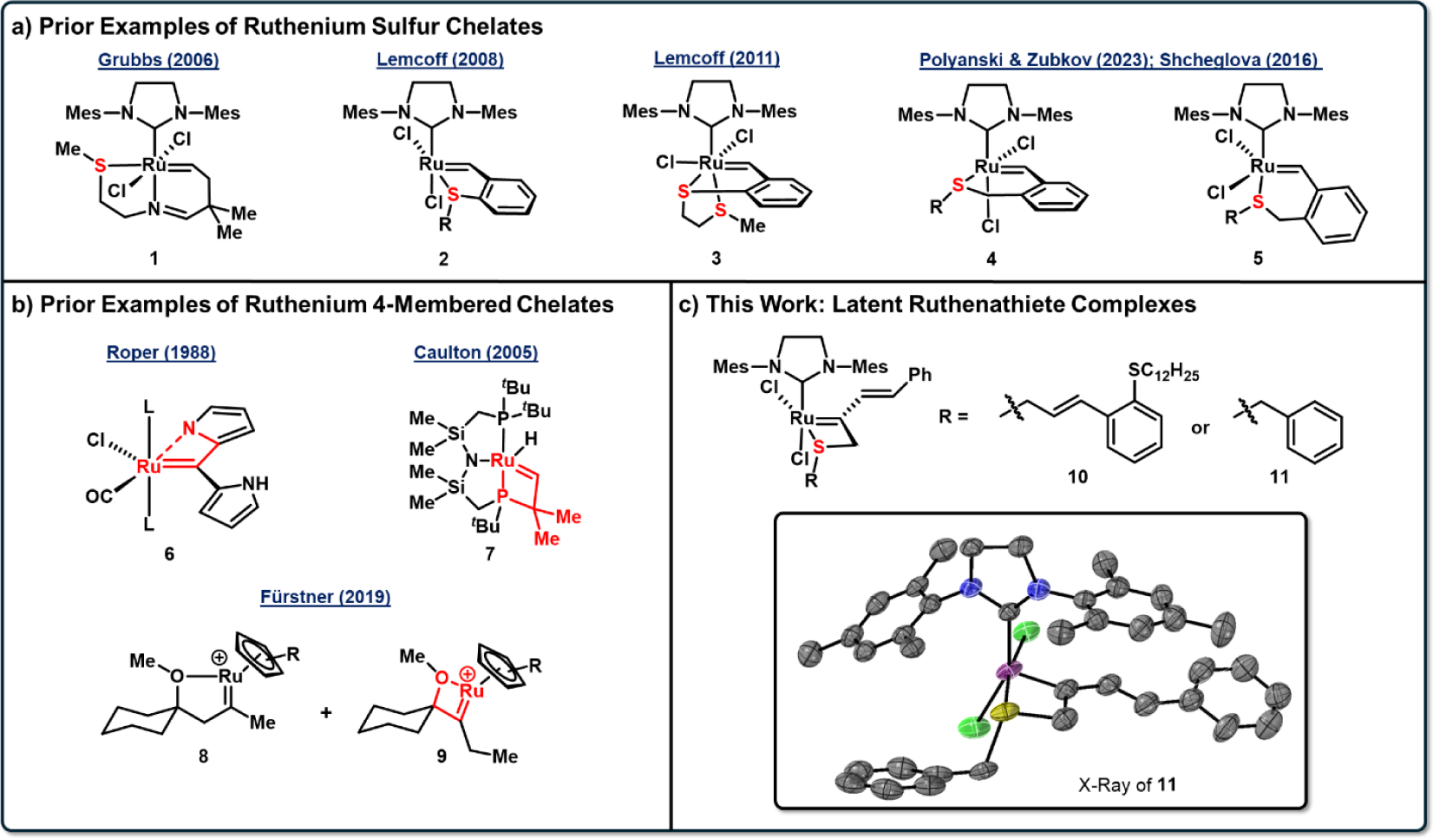
a) Dichloro 5- and 6-membered sulfur ruthenium chelate complexes developed. b) Four-membered ruthenium alkylidene chelates to nitrogen, phosphorus, and oxygen. c) 4-membered ruthenathiete chelate complexes **10** and **11** developed in this work.

## Results and Discussion

Previous research in our laboratory investigated the ability of terminal alkynes to serve as directing groups in metathesis processes to produce new monomers, reagents for in situ catalyst modification, and reagents for ROMP termination.^41–44^ During our studies on termination, a variety of 1,6-enynes were explored to determine the impact of the central heteroatom on the cascade metathesis reaction. In this process, rapid addition of the Grubbs third-generation catalyst (**G3**) across the terminal alkyne is followed by an intramolecular cyclization onto the pendant alkene to yield a Lemcoff-type ruthenium complex (**13**) that is inert toward further metathesis (Figure 2a).^42^ Ultimately, sulfonamide-linked enynes were found to be the most efficient, followed by amide and ether linked enynes. Motivated by prior research from Davis and coworkers who demonstrated allylic thioethers undergo accelerated cross metathesis compared to allylic ethers and amines,^45^ thioether-linked 1,6-enyne **12** was prepared and investigated (Figure 2b). Initial reaction between 1.3 eq of enyne **12** and **G3** showed complete consumption of the reactants, but the expected product **13** was not observed in the crude ^1^H NMR spectrum. Purification of the reaction mixture revealed a new ruthenium complex, and the ^1^H and ^13^C NMR spectra were consistent with the ruthenathiete complex **10**. Particularly diagnostic was the presence of an alkylidene ^13^C resonance at 273 ppm that is more shielded than other disubstituted alkylidenes, such as indenylidenes (Figures S1 and S2).^46,47^ The isolation of this complex was particularly surprising, as the coordination to sulfur completely inhibited the *intra*molecular cyclization onto the pendant alkene, an intermediate that is not observed in other enyne systems (Figure 2a). No additional reaction was observed upon heating **10** at 60 °C for 2 h in chloroform (CDCl_3_), and only 45% conversion to chelate **13** and 2,5-dihydrothiophene (**14**) occurred after heating to 100 °C for 4 h (Figures S5 and S6). Unfortunately, all attempts to obtain crystals suitable for single crystal X-ray diffraction (SCXRD) to confirm the ruthenathiete structure were unsuccessful.

**Figure 2:**
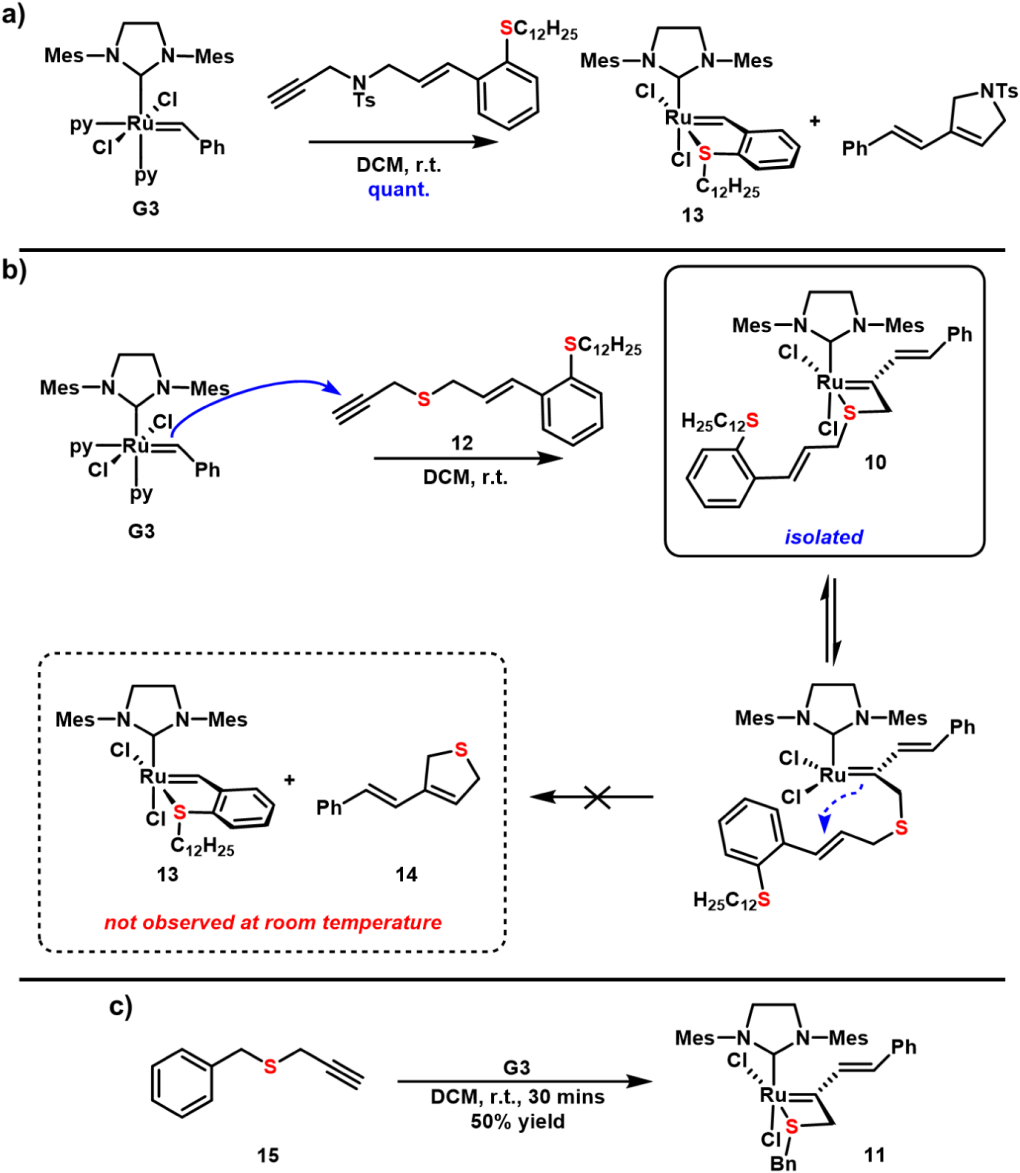
a) Enyne metathesis cascade reaction to generate complex **13**. b) Intercepted enyne cascade mechanism that leads to ruthenathiete **10**. c) Synthesis of complex **11** from Grubbs third-generation catalyst (**G3**) and enyne **15**.

The remarkable resistance of complex **10** toward an intramolecular cyclization prompted further study of ruthenathietes as latent initiators for alkene metathesis. As the pendant alkene would thwart the study of intermolecular metathesis reactions, a truncated 4-membered chelate derived from benzyl propargyl thioether (**15**) was investigated (Figure 2c). **G3** cleanly reacted with **15** to give the green ruthenathiete complex **11** in 50% yield after column chromatography. Gratifyingly, 2:1 cocrystals of toluene and **11** suitable for SCXRD were obtained via vapor diffusion of hexanes into a toluene solution of **11**. Analysis of the structure confirmed the anticipated 4-membered sulfur chelate in a distorted square pyramidal geometry (Figure 3). There are a few notable characteristics seen in the crystal structure of complex **11**. The Ru–S bond length is 2.36 Å, which agrees with L-type sulfur chelates in the Cambridge Crystallographic Database (2.3 Å–2.4 Å).^29,30^ The S1–Ru1–C22 bond angle is 80.7° which lies between reported 4-membered Ru chelates with nitrogen and phosphorus reported by Roper and Caulton (90.9° and 68.6°, respectively).^38,40^ The C23–C22–S1 bond angle is 97.6° and the Ru1–C23–C22 is 109.0° which are contracted compared to sp^3^- and sp^2^-hybridized carbon bond angles, respectively. The sulfur atom is a chiral center that exhibits a pyramidal structure with the benzyl group pointing downward, away from the *N*-heterocyclic carbene (NHC), to minimize steric interactions between the bulky mesityl substituents and benzyl group similarly to what was noted by Zubkov.^30^ A C–H−π interaction can be observed between the methyl hydrogen of each mesityl and the styrene and benzyl π-systems (Figures 3, S22). The H20c–styrene interaction was characterized by an H−π centroid distance of 3.33 Å, C–H−π centroid angle of 154.8°, and a distance between the styrene centroid and the mesityl methyl carbon of 4.33 Å.^48,49^ The H12c–benzyl interaction exhibited an H−π centroid distance of 2.88 Å, C–H−π centroid angle of 165.7°, and C-centroid distance of 3.96 Å. The mesityl methyl C12, which is responsible for the H−π interaction of H12c with the benzyl thioether moiety, is bent out of plane of the mesityl phenyl ring with torsional angles of 8.8° (N1–C4–C9–C12), −168.5° (C5–C4–C9–C12), and 170.4° (C7–C8–C9–C12) (Figure S23). Additionally, the distance between the mesityl *ipso* carbon (C13) and the alkylidene carbon (C23) is 3.16 Å (Figure S24). This is comparable to other ruthenium NHC complexes which show a π–π* interaction between the mesityl and the π* orbital on the carbene, leading to increased stabilization of NHC complexes.^50,51^

**Figure 3:**
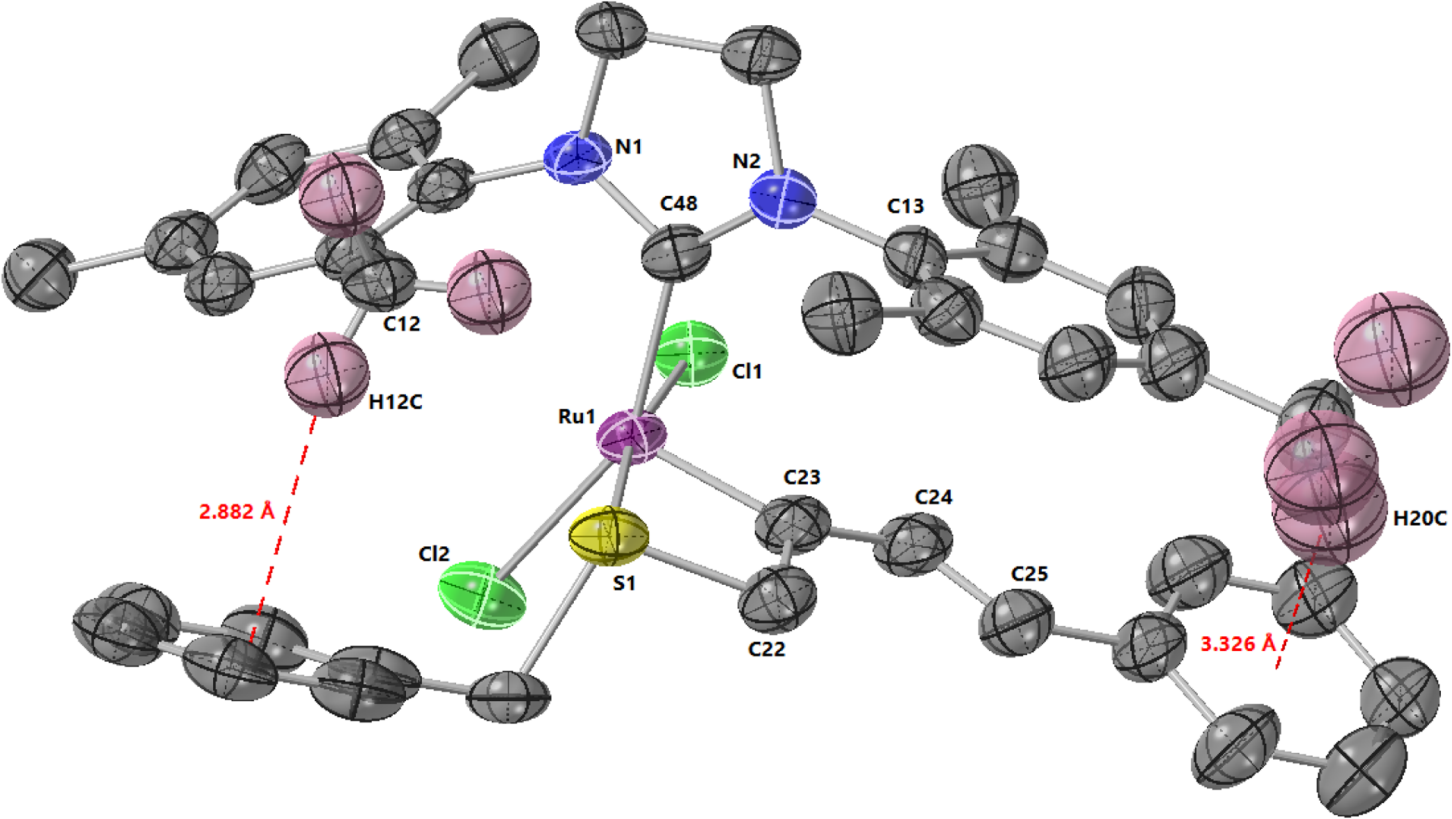
Crystal structure of catalyst **11** determined by X-ray crystallography. Thermal ellipsoids were drawn at 50% probability. Hydrogens (aside from the ones discussed) and solvent molecules were omitted for clarity. Selected bond lengths/angles: Ru1–S1 bond: 2.36 Å, S1–Ru1–C22:80.7°, C23–C22–S1:97.6°, Ru1–C23–C22:109.0°, C23–Ru1–S1:72.4°.

The reactivity of **11** was probed using ring-opening metathesis polymerization (ROMP), ring-closing metathesis (RCM), and cross-metathesis (CM). First, the ROMP of *N*-benzyl norbornene imide **16** was explored under ambient light and in the dark. No conversion of **16** was observed after 7 h in CDCl_3_ at room temperature for either experiment (Figure S19b,c). However, heating solutions of **16** to 35 °C at 0.5 M resulted in 96% conversion after 100 min, as measured by ^1^H NMR (Figure 4, entry 1). Further characterization of this polymer was not possible by size-exclusion chromatography (SEC) due to gelation, possibly due to very high molecular weights obtained by slow initiation of **11**, regardless of initiation temperature (35 °C–100 °C, Figure S20). Transitioning to a monomer that would result in a more soluble polymer, *cis*-cyclooctene (**17**) was examined. Poly­(**17**) was generated in 99% conversion after 1 h at 100 °C and was able to be analyzed by SEC (Figure 4, entry 2, *M*_*n*_: 72.4 kDa, and *Đ*: 2.80). The latency of this catalyst was further probed through the RCM in CDCl_3_ of diallyl sulfonamide **18a** at 0.1 M with respect to substrate and 5 mol % catalyst loading (Figure 5). It was found that **11** led to no conversion at 25 °C for 45 min (Figure 5, blue region) and increasing the reaction temperature to 40 °C resulted in approximately 1% conversion after an additional 60 min (Figure 5, green region). Raising the temperature to 60 °C resulted in an increase of reaction rate, ultimately reaching 82% conversion after 35 h.

**Figure 4:**
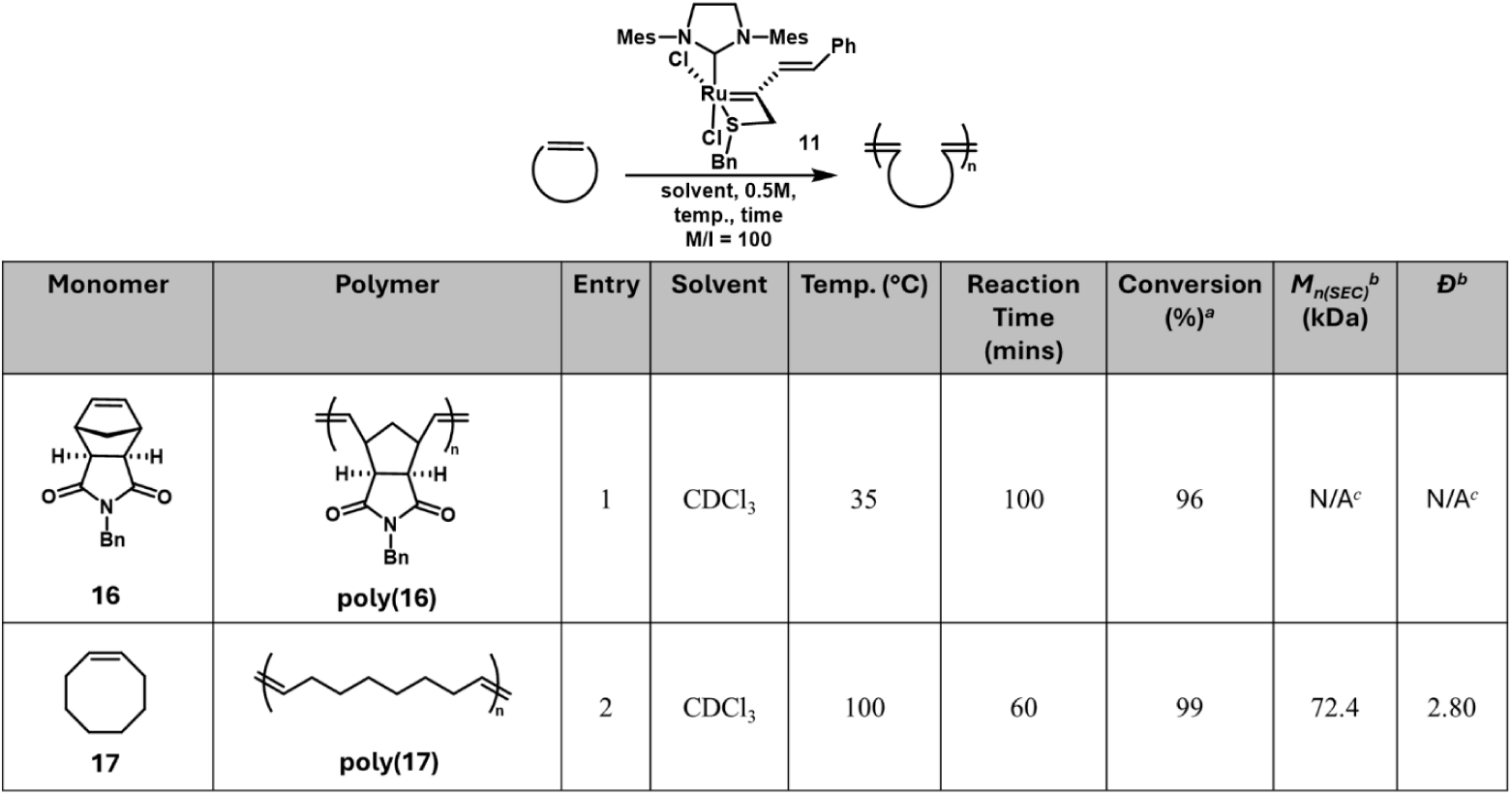
Ring-opening metathesis polymerization (ROMP) of cyclic olefins using complex **11.** ^a^Conversion was monitored via ^1^H NMR. ^b^Experimentally determined molecular weights (**M_n(SEC)_**) and dispersities (**Đ**) are based on size exclusion chromatography (SEC) in CHCl_3_ and calibrated with polystyrene standards. ^c^ **M**_**n**(**SEC)**_ and **Đ** were unable to be determined for **16** due to insolubility.

**Figure 5:**
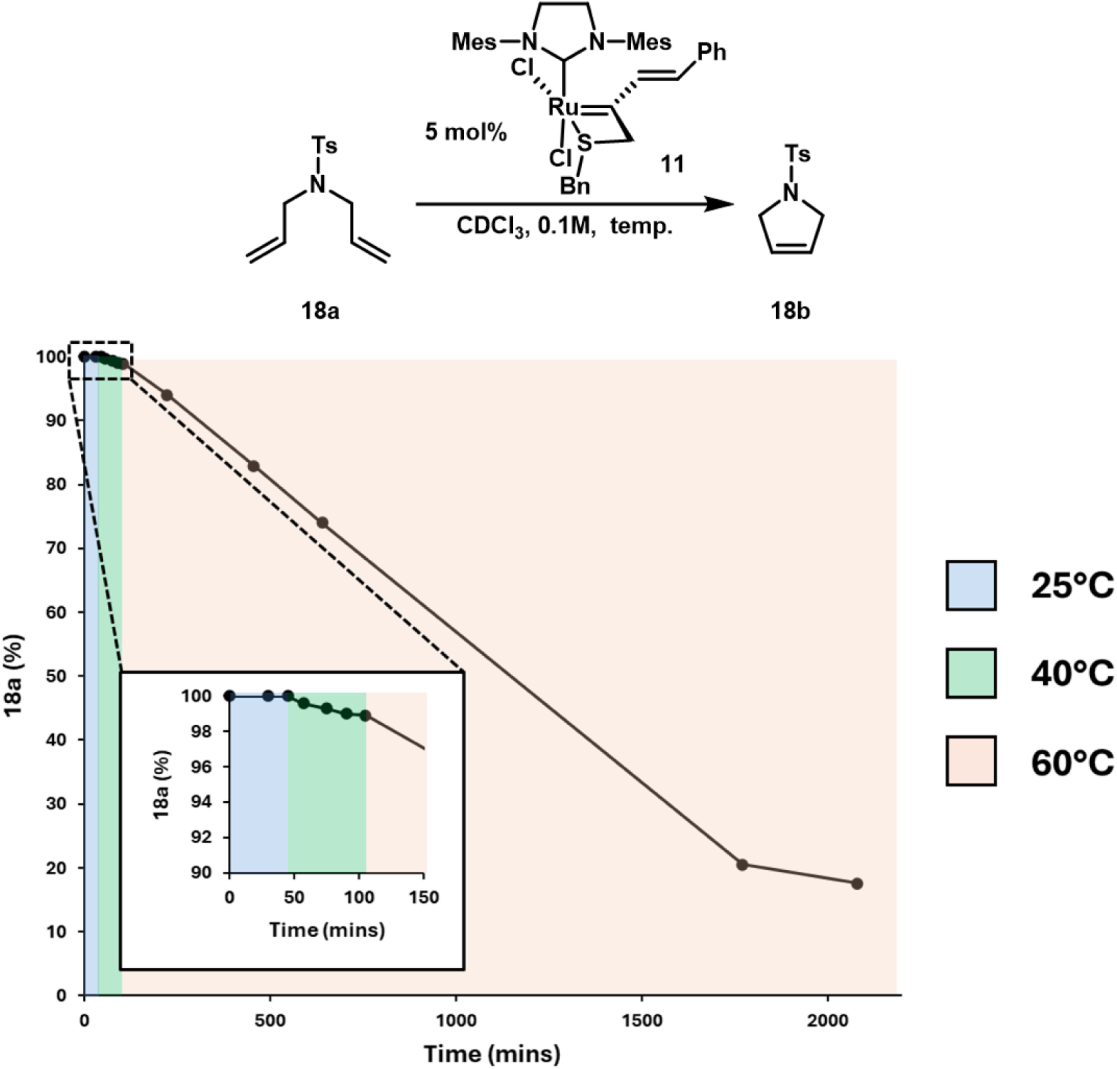
Conversion profile of the RCM of **18a** in CDCl_3_ at 0.1 M with 5 mol % **11** at temperatures of 25 °C, 40 °C, and 60 °C.

Based on this VT-NMR study, all further RCM and CM experiments were performed in sealed microwave vials at different temperatures and reaction times at 0.1 M with 5 mol % catalyst loading. After 24 h in CDCl_3_ at 60 °C, only 46% conversion of **18a** to **18b** was achieved (Figure 6, entry 1). However, increasing the temperature to 100 °C resulted in 99% conversion after 24 h, and decreasing the reaction time to 1 h yielded 73% conversion at this temperature (Figure 6, entries 2, 3). Further substrates that were explored for RCM and CM, including diallyl malonate **19a**, *gem*-disubstituted sulfonamide **20a**, styrene 2**1a**, and 5-bromopentene **22a**. For the RCM of diallyl malonate **19a**, 61% and 98% conversion to the cyclopentene product **19b** was observed after 24 h at 60 and 100 °C, respectively (Figure 6, entries 10, 11). To benchmark reactivity of complex **11** with respect to a 5-membered chelate, the RCM of diallyl malonate **19a** was conducted following reaction parameters reported by Lemcoff in tol-*d*_8_ at 0.1 M with 1 mol % catalyst loading at 90 °C for 24 h and open to air. The RCM reached 92% conversion after the allotted time, which is higher than five-membered chelate **2** (R = *i*Pr) that reached 84% conversion after 2 days under the same conditions (Figure 6, entry 12).^5,29^ More challenging RCM substrates like *gem*-disubstituted olefins were tolerated as sulfonamide **20a** produced pyrroline **20b** in 26% and 95% conversion at 60 and 100 °C, respectively (Figure 6, entries 13, 14). The cross-metathesis of styrene (**21a**) to *trans*-stilbene (**21b**) proceeded more slowly, reaching 26% and 85% conversion at 60 and 100 °C, respectively (Figure 6, entries 15, 16). The CM of a more electron-rich olefin such as 5-bromopentene (**22a**) resulted in similar conversions as styrene, with **22b** being formed in 21% and 83% conversion at 60 and 100 °C, respectively (Figure 6, entries 17, 18).

**Figure 6:**
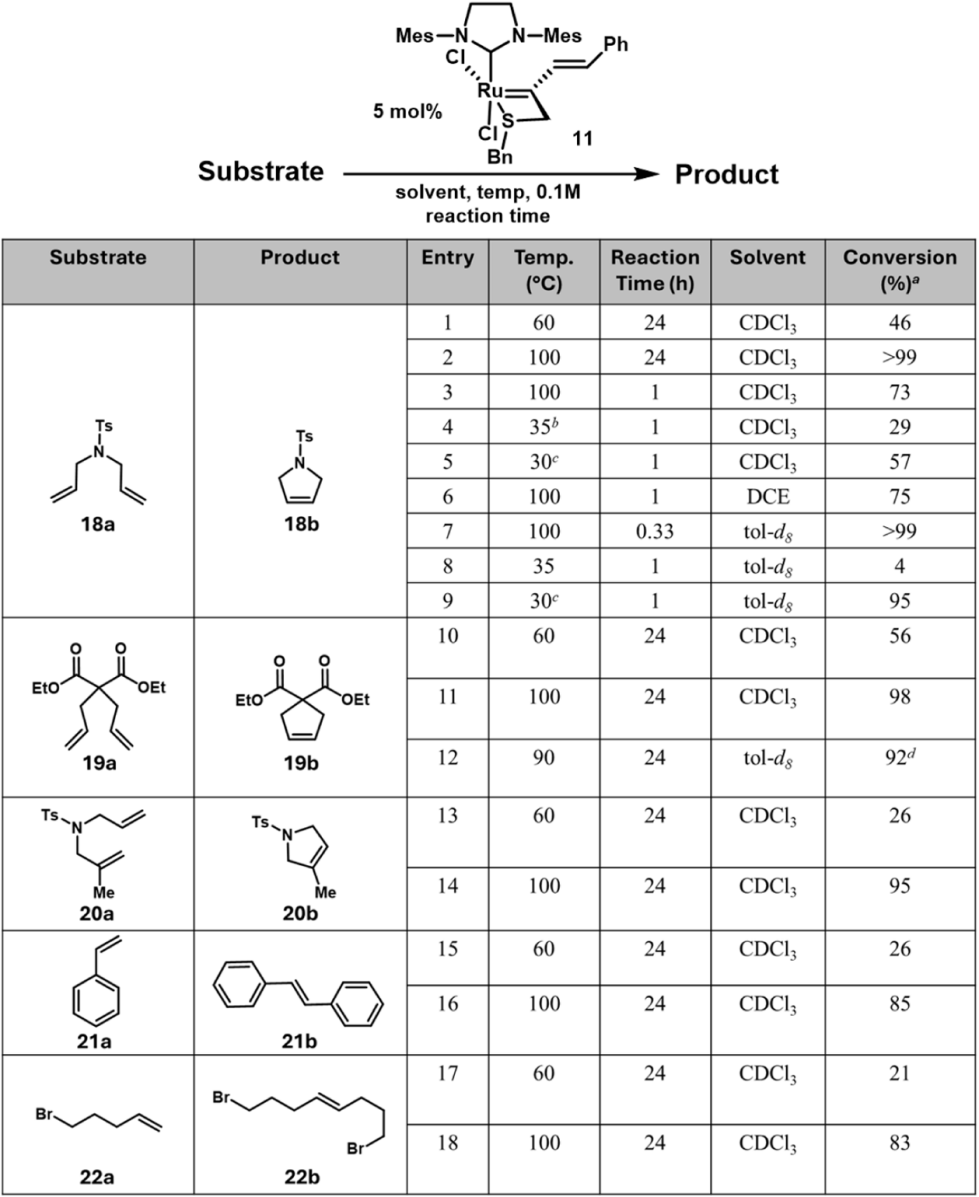
Substrate scope of metathesis reactions catalyzed by **11**. ^a^Conversion was monitored via ^1^H NMR. ^b^UV irradiation (300 nm) with sample temperature at 35 °C. ^c^UV irradiation (390 nm) with sample temperature at 30 °C. ^d^1 mol % instead of 5 mol %, open to air.

Next, the effect of solvent on metathesis reactions was investigated. For the RCM of **18a**, changing the solvent from CDCl_3_ to dichloroethane (DCE) afforded a similar conversion after 1 h at 100 °C (75%, Figure 6, entry 6). Interestingly, the less polar deuterated toluene (tol-*d*_8_) solvent resulted in a higher reaction rate than either of the chlorinated solvents, with >99% conversion observed after only 20 min at 100 °C (Figure 6, entry 7). Attempting an RCM in toluene at 35 °C yielded **18b** in only 4% conversion after 1 h which is higher than the same reaction carried out in CDCl_3_ at 40 °C which reached about 1% conversion according to the VT-NMR study (Figure 6, entry 8). These results are consistent with prior observations from Fürstner that aromatic solvents can disrupt the stabilizing π-interactions of the NHC mesityl with the alkylidene moiety, allowing for alkylidene bond rotation, as well as observations from Lemcoff on the greater persistence of the *trans* isomer in less polar solvents.^52–54^ The disruption of intramolecular interactions in complex **11** is further supported by changes in the chemical shifts and splitting patterns of the NHC proton resonances in the ^1^H NMR spectrum of catalyst **11** in tol-*d*_8_ compared to CDCl_3_ (Figure S11). To see whether these solvent effects were also operative in the ROMP of COE, polymerization samples with a **17**:**11** ratio of 100:1 were heated to 60 °C for 45 min in toluene and chloroform. It was found that polymerizations in tol-*d*_8_ resulted in significantly higher conversion (94%) than in CDCl_3_ (34%, Table S1). An increase in reactivity in tol-*d*_8_ was also observed when revisiting the intramolecular cyclization of complex **10** (Figure 2b). 17% conversion to 5-membered chelate **13** and dihydrothiophene **14** was observed upon heating a solution of **10** in tol-*d*_8_ for 2 h at 60 °C, whereas no conversion was observed in CDCl_3_ (Figure S3). Complete conversion of **10** was obtained when heating in tol-*d*_8_ at 100 °C for 3 h (Figure S4).

To further examine the stability of **11** in these two solvents, solutions in CDCl_3_ and tol-*d*_8_ were left open to air for multiple days. After 5 days at room temperature in tol-*d*_8_, **11** showed 89% conversion to a characteristic aldehyde degradation product (Figure S17), whereas only 16% of aldehyde is observed after 10 days in CDCl_3_ (Figure S18). Due to the enhanced stability of the catalyst in CDCl_3_, additional experiments were performed. **11** was heated in CDCl_3_ at 100 °C under inert atmosphere for 20 h and no significant changes were observed by ^1^H NMR spectroscopy (Figure S16). Next, this catalyst solution was then heated to 50 °C for 24 h while open to air (reaction vial with open vent needle) which resulted in minimal changes to the ^1^H NMR spectrum of **11** and negligible formation of aldehyde oxidation product. Given the more significant formation of aldehyde at lower temperatures, the CDCl_3_ vapor may be shielding oxygen from entering the system efficiently. This highlights the potential of catalyst **11** in air-resistant thermoset resin formulations.^55^

UV light was also tested as a stimulus for initiation by irradiating reaction solutions with 300 or 390 nm light at room temperature. Exposure of **18a** and **11** (5 mol %) to 300 nm light for 1 h in CDCl_3_ resulted in 29% conversion to ring-closed **18b** (Figure 6, entry 4). While irradiation raised the reaction temperature to 35 °C, the significant increase in conversion compared to the nonirradiated experiment at 40 °C (∼1% conversion) suggests a photochemically induced reaction. 390 nm light afforded much higher conversion of **18a** to ring-closed **18b** than 300 nm light (57%, Figure 6 entry 5), which is consistent with a strong absorption band in the UV–vis spectrum of **11** between 350 and 450 nm (Figure S25). The solvent effects observed for thermal initiation were also observed for UV-irradiation, as exposure of **18a** and chelate **11** to 390 nm light in toluene increased conversion to 95% after 1 h (Figure 6, entry 9).

Finally, the mechanism of catalysis was explored. When 5-membered ruthenium chelates (e.g., **2** and **13**) are prepared through reaction with an appropriate enyne or styrene derivative, a *trans*-dichloro isomer is kinetically formed that slowly isomerizes to the more stable *cis* isomer.^29,42^ Heating of the *cis* isomer has been able to reestablish the equilibrium and serves as evidence for the *trans*-dichloride being the catalytically active species in metathesis reactions. In an attempt to observe the *trans-*dichloro isomer of complex **11** by heating, ^1^H and ^13^C NMR experiments were performed. ^1^H NMR analysis after heating to 100 °C in CDCl_3_ and VT-NMR studies in 1,1,2,2-tetrachloroethane-*d*_2_ (TCE-*d*_2_) also resulted in no change to the ^1^H and ^13^C NMR spectra (Figures S12, S13 and S14).^7,41^ In an attempt to observe the *trans*-**11** before isomerization to the *cis* isomer, 1.3 equiv of alkyne **15** was added to a solution of **G3** in CDCl_3_ (1 mM) starting at 0 °C and allowed to warm to room temperature. ^1^H NMR analysis after 7 min showed only *cis-* **11** and displaced pyridine ligand (Figure S9). Because of the greater presence of the *trans* isomer in nonpolar solvents for 5-membered chelates,^53^ the same experiment was performed in tol-*d*_8_ solvent but only the *cis* product was observed under these conditions, as well (Figure S10). The existence of any *trans*-**11** complex appears to be very short-lived, with conversion to the more stable *cis*-**11** isomer occurring at much faster rates than other sulfur-chelated ruthenium complexes. The disubstituted ruthenium alkylidene in *trans*-**11** would present significant steric interactions between the mesityl and the styryl groups, likely explaining the inability to directly observe this isomer (Scheme 1). At this time, it is unclear whether the observed catalytic activity of *cis*-**11** is a result of transient isomerization to *trans*-**11** or the generation of an unobserved catalytically active degradation product.

**Scheme 1:**
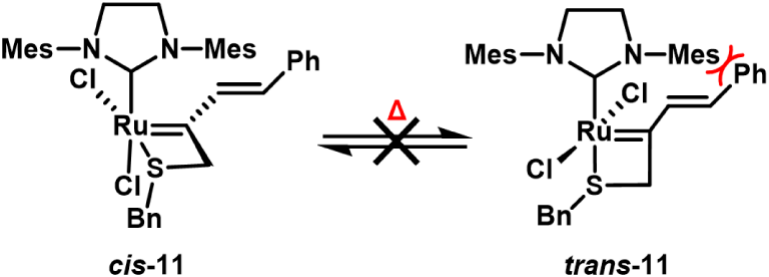
Proposed Rationale for the Inability to Observe *trans*-Dichloro Complex **11**

## Conclusions

In conclusion, the first *cis*-dichloro ruthenathiete complexes **10** and **11** were synthesized, characterized, and benchmarked for reactivity in olefin metathesis reactions. These complexes show no reactivity toward metathesis at room temperature but readily performed catalytic olefin metathesis at elevated temperatures or upon exposure to UV irradiation. Ruthenium complex **11** was able to carry out a variety of metathesis reactions including ring-closing metathesis, cross-metathesis and ring-opening metathesis with latent reactivity is comparable to the 5-membered sulfur chelates developed by Lemcoff and maintained high thermal and air stability in solution. Interestingly, the presence of H−π interactions were observed between the NHC and the alkylidene substituents, which likely contributes to the stability of these complexes. Disruption of these interactions by aromatic solvents, such as toluene, resulted in increased reactivity and increased degradation rates of the **11** in air. Overall, this introduces a new subset of sulfur-chelated ruthenium complexes for use when spatiotemporal control over catalytic processes is advantageous, such as 3D printing or materials patterning.

## Supplementary Material





## Notes

Synthetic and experimental methods; crystallographic data of CCDC2521413; NMR spectra; UV–vis spectra (PDF)
Text file of Cartesian coordinates of CCDC2521413 for convenient viewing (XYZ)

## References

[1] Fraser C., Grubbs R. H. (1995). Synthesis of Glycopolymers of Controlled Molecular
Weight by Ring-Opening Metathesis Polymerization Using Well-Defined
Functional Group Tolerant Ruthenium Carbene Catalysts. Macromolecules.

[2] Dewaele A., Van Berlo B., Dijkmans J., Jacobs P. A., Sels B. F. (2016). Immobilized
Grubbs Catalysts on Mesoporous Silica Materials: Insight into Support
Characteristics and Their Impact on Catalytic Activity and Product
Selectivity. Catal. Sci. Technol..

[3] Peterson G. I., Yang S., Choi T.-L. (2019). Synthesis
of Functional Polyacetylenes
via Cyclopolymerization of Diyne Monomers with Grubbs-Type Catalysts. Acc. Chem. Res..

[4] Nguyen S. T., Johnson L. K., Grubbs R. H., Ziller J. W. (1992). Ring-Opening Metathesis
Polymerization (ROMP) of Norbornene by a Group VIII Carbene Complex
in Protic Media. J. Am. Chem. Soc..

[5] Kost T., Sigalov M., Goldberg I., Ben-Asuly A., Lemcoff N. G. (2008). Latent Sulfur Chelated Ruthenium
Catalysts: Steric
Acceleration Effects on Olefin Metathesis. J.
Organomet. Chem..

[6] P’Poo S. J., Schanz H.-J. (2007). Reversible Inhibition/Activation
of Olefin Metathesis:
A Kinetic Investigation of ROMP and RCM Reactions with Grubbs’
Catalyst. J. Am. Chem. Soc..

[7] Hejl A., Day M. W., Grubbs R. H. (2006). Latent
Olefin Metathesis Catalysts
Featuring Chelating Alkylidenes. Organometallics.

[8] Gułajski Ł., Michrowska A., Bujok R., Grela K. (2006). New Tunable Catalysts
for Olefin Metathesis: Controlling the Initiation through Electronic
Factors. J. Mol. Catal. A: Chem..

[9] Vaisman A., Tafazolian H., Stoianova D., Goldberg J. M., Goulinian K., Doppiu A., Johns A. M., Lemcoff N. G. (2025). Latent Ruthenium
Complexes Supported by Two N-Heterocyclic Carbene Ligands: Synthesis
and Catalytic Activity with Distinctive Activation. Eur. J. Org. Chem..

[10] Szadkowska A., Gstrein X., Burtscher D., Jarzembska K., Woźniak K., Slugovc C., Grela K. (2010). Latent Thermo-Switchable
Olefin Metathesis Initiators Bearing a Pyridyl-Functionalized Chelating
Carbene: Influence of the Leaving Group’s Rigidity on the Catalyst’s
Performance. Organometallics.

[11] Grzesiński Ł., Milewski M., Nadirova M., Kajetanowicz A., Grela K. (2023). Unexpected Latency of Z-Stereoretentive
Ruthenium Olefin Metathesis
Catalysts Bearing Unsymmetrical N-Heterocyclic Carbene or Cyclic­(Alkyl)­(Amino)­Carbene
Ligands. Organometallics.

[12] Gawin R., Makal A., Woźniak K., Mauduit M., Grela K. (2007). A Dormant
Ruthenium Catalyst Bearing a Chelating Carboxylate Ligand: In Situ
Activation and Application in Metathesis Reactions. Angew. Chem. Int. Ed..

[13] Eivgi O., Vaisman A., Nechmad N. B., Baranov M., Lemcoff N. G. (2020). Latent
Ruthenium Benzylidene Phosphite Complexes for Visible-Light-Induced
Olefin Metathesis. ACS Catal..

[14] Monsigny L., Cejas Sánchez J., Piątkowski J., Kajetanowicz A., Grela K. (2021). Synthesis and Catalytic Properties of a Very Latent Selenium-Chelated
Ruthenium Benzylidene Olefin Metathesis Catalyst. Organometallics.

[15] Ben-Asuly A., Aharoni A., Diesendruck C. E., Vidavsky Y., Goldberg I., Straub B. F., Lemcoff N. G. (2009). Photoactivation
of Ruthenium Olefin
Metathesis Initiators. Organometallics.

[16] Almuzaini H. N., Slebodnick C., Schulz M. D. (2023). Exploring the Formation of Copper–Ruthenium
Bimetallic Complexes in Olefin Metathesis. Organometallics.

[17] Bell M., Hester H. G., Gallman A. N., Gomez V., Pribyl J., Rojas G., Riegger A., Weil T., Watanabe H., Chujo Y., Wagener K. B. (2019). Bulk Acyclic Diene Metathesis Polycondensation. Macromol. Chem. Phys..

[18] Marx V. M., Sullivan A. H., Melaimi M., Virgil S. C., Keitz B. K., Weinberger D. S., Bertrand G., Grubbs R. H. (2015). Cyclic Alkyl Amino
Carbene (CAAC) Ruthenium Complexes as Remarkably Active Catalysts
for Ethenolysis. Angew. Chem. Int. Ed..

[19] Greenlee A. J., Weitekamp R. A., Foster J. C., Leguizamon S. C. (2024). PhotoROMP:
The Future Is Bright. ACS Catal..

[20] Fowler H. E., Taylor M. S., Nguyen C. P. H., Boese D. A., Baca E., Greenlee A. J., Kaufman G. E., Gallegos M. A., Huntley E. F., Appelhans L. N., Kaehr B., Leguizamon S. C. (2025). Frontal
Polymerization of Thermosets to Enable Vacuum-Formed Structural Electronics. Nat. Commun..

[21] Lloyd E. M., Feinberg E. C., Gao Y., Peterson S. R., Soman B., Hemmer J., Dean L. M., Wu Q., Geubelle P. H., Sottos N. R., Moore J. S. (2021). Spontaneous Patterning
during Frontal
Polymerization. ACS Cent. Sci..

[22] Darby D. R., Greenlee A. J., Bean R. H., Fairchild D. C., Rodriguez V. C., Jansen A. L., Gallegos S. C., Ramirez S. P., Moore J. S., Leguizamon S. C., Appelhans L. N. (2025). Active
Light-Controlled Frontal Ring-Opening Metathesis Polymerization. Nat. Commun..

[23] Robertson I. D., Dean L. M., Rudebusch G. E., Sottos N. R., White S. R., Moore J. S. (2017). Alkyl Phosphite
Inhibitors for Frontal Ring-Opening
Metathesis Polymerization Greatly Increase Pot Life. ACS Macro Lett..

[24] Leguizamon S. C., Lyons K., Monk N. T., Hochrein M. T., Jones B. H., Foster J. C. (2022). Additive Manufacturing
of Degradable Materials via
Ring-Opening Metathesis Polymerization (ROMP). ACS Appl. Mater. Interfaces.

[25] Rylski A. K., Cater H. L., Mason K. S., Allen M. J., Arrowood A. J., Freeman B. D., Sanoja G. E., Page Z. A. (2022). Polymeric Multimaterials
by Photochemical Patterning of Crystallinity. Science.

[26] Eivgi O., Guidone S., Frenklah A., Kozuch S., Goldberg I., Lemcoff N. G. (2018). Photoactivation of Ruthenium Phosphite Complexes for
Olefin Metathesis. ACS Catal..

[27] Kumandin P. A., Antonova A. S., Alekseeva K. A., Nikitina E. V., Novikov R. A., Vasilyev K. A., Sinelshchikova A. A., Grigoriev M. S., Polyanskii K. B., Zubkov F. I. (2020). Influence of the
N→Ru Coordinate
Bond Length on the Activity of New Types of Hoveyda–Grubbs
Olefin Metathesis Catalysts Containing a Six-Membered Chelate Ring
Possessing a Ruthenium–Nitrogen Bond. Organometallics.

[28] Kingsbury J. S., Harrity J. P. A., Bonitatebus P. J., Hoveyda A. H. (1999). A Recyclable Ru-Based
Metathesis Catalyst. J. Am. Chem. Soc..

[29] Ben-Asuly A., Tzur E., Diesendruck C. E., Sigalov M., Goldberg I., Lemcoff N. G. (2008). A Thermally
Switchable Latent Ruthenium Olefin Metathesis
Catalyst. Organometallics.

[30] Kumandin P. A., Antonova A. S., Novikov R. A., Vasilyev K. A., Vinokurova M. A., Grigoriev M. S., Novikov A. P., Polianskaia D. K., Polyanskii K. B., Zubkov F. I. (2023). Properties and Catalytic Activity
of Hoveyda–Grubbs-Type Catalysts with an S → Ru Coordination
Bond in a Six-Membered Chelate Ring. Organometallics.

[31] Yang H.-C., Huang Y.-C., Lan Y.-K., Luh T.-Y., Zhao Y., Truhlar D. G. (2011). Carbene Rotamer Switching Explains the Reverse Trans
Effect in Forming the Grubbs Second-Generation Olefin Metathesis Catalyst. Organometallics.

[32] Occhipinti G., Nascimento D. L., Foscato M., Fogg D. E., Jensen V. R. (2022). The Janus
Face of High Trans-Effect Carbenes in Olefin Metathesis: Gateway to
Both Productivity and Decomposition. Chem. Sci..

[33] Diesendruck C. E., Vidavsky Y., Ben-Asuly A., Lemcoff N. G. (2009). A Latent S-Chelated
Ruthenium Benzylidene Initiator for Ring-Opening Metathesis Polymerization. J. Polym. Sci. Polym. Chem..

[34] Aharoni A., Vidavsky Y., Diesendruck E., Ben-Asuly A., Goldberg I., Gabriel Lemcoff N. G. (2011). Ligand
Isomerization in Sulfur-Chelated
Ruthenium Benzylidenes. Organometallics.

[35] Ginzburg Y., Anaby A., Vidavsky Y., Diesendruck C. E., Ben-Asuly A., Goldberg I., Lemcoff N. G. (2011). Widening
the Latency
Gap in Chelated Ruthenium Olefin Metathesis Catalysts. Organometallics.

[36] Segalovich-Gerendash G., Baranov M., Lemcoff N. G., Phatake R. S. (2023). Ruthenium Olefin
Metathesis Catalysts with Six-Membered Chelating Dithioacetal Ligands:
Synthesis and Reactivity. Organometallics.

[37] Shcheglova N. M., Kolesnik V. D., Ashirov R. V., Krasnokutskaya E. A. (2016). Latent
Ruthenium Carbene Complexes with Six-Membered N- and S-Chelate Rings. Russ. Chem. Bull..

[38] Ingleson M. J., Yang X., Pink M., Caulton K. G. (2005). [(TBu2PCH2SiMe2)­2N]­RuCH3:
The Origin of Extremely Facile, Double H–C­(Sp3) Activation
Generating a “Hydrido-Carbene” Complex. J. Am. Chem. Soc..

[39] Biberger T., Gordon C. P., Leutzsch M., Peil S., Guthertz A., Copéret C., Fürstner A. (2019). Alkyne Gem-Hydrogenation: Formation
of Pianostool Ruthenium Carbene Complexes and Analysis of Their Chemical
Character. Angew. Chem. Int. Ed..

[40] Clark G. R., Hodgson D. J., Ng M. M. P., Rickard C. E. F., Roper W. R., Wright L. J. (1988). Transition Metal Complexes of the Dipyrromethylidene
(Dipyrrol-2-Ylmethylene) Ligand, LM­[=C­(C4H4N)­2], from Reaction between
Ruthenium- and Osmium-Bound Dichlorocarbene and Pyrrole: Structures
of [RuCl2­{=CCl­(C4H4N)}­(CO)­(PPh3)­2] and [RuCl­{=C­(C4H3N)­(C4H4N)}­(CO)­(PPh3)­2]. J. Chem. Soc., Chem. Commun..

[41] Zhang T., Fu L., Gutekunst R. W. (2018). Practical
Synthesis of Functional Metathesis Initiators
Using Enynes. Macromolecules.

[42] Fu L., Zhang T., Fu G., Gutekunst R. W. (2018). Relay Conjugation
of Living Metathesis Polymers. J. Am. Chem.
Soc..

[43] Fu L., Sui X., Crolais A. E., Gutekunst W. R. (2019). Modular Approach to Degradable Acetal
Polymers Using Cascade Enyne Metathesis Polymerization. Angew. Chem. Int. Ed..

[44] Pabarue A. B., Sui X., Bu Y., Xiong W., Gutekunst W. R. (2025). Ring-Opening
Metathesis Polymerization of Levoglucosenone-Derived Enyne Monomers. J. Polym. Sci..

[45] Lin Y. A., Chalker J. M., Floyd N., Bernardes G. J. L., Davis B. G. (2008). Allyl Sulfides Are Privileged Substrates
in Aqueous
Cross-Metathesis: Application to Site-Selective Protein Modification. J. Am. Chem. Soc..

[46] Fürstner A., Guth O., Düffels A., Seidel G., Liebl M., Gabor B., Mynott R. (2001). Indenylidene
Complexes of Ruthenium:
Optimized Synthesis, Structure Elucidation, and Performance as Catalysts
for Olefin Metathesis—Application to the Synthesis of the ADE-Ring
System of Nakadomarin A. Chem. - Eur. J..

[47] Nascimento D. L., Gawin A., Gawin R., Guńka P. A., Zachara J., Skowerski K., Fogg D. E. (2019). Integrating Activity
with Accessibility in Olefin Metathesis: An Unprecedentedly Reactive
Ruthenium-Indenylidene Catalyst. J. Am. Chem.
Soc..

[48] Wang J., Yao L. (2019). Dissecting C–H•••π and N–H•••π
Interactions in Two Proteins Using a Combined Experimental and Computational
Approach. Sci. Rep..

[49] Brandl M., Weiss M. S., Jabs A., Sühnel J., Hilgenfeld R. (2001). C-H···π-Interactions
in Proteins. J. Mol. Biol..

[50] Fernández I., Lugan N., Lavigne G. (2012). Effects of
Attractive Through Space
π–Π* Interactions on the Structure, Reactivity,
and Activity of Grubbs II Complexes. Organometallics.

[51] Süßner M., Plenio H. (2005). π-Face Donor Properties of N-Heterocyclic Carbenes. Chem. Commun..

[52] Fürstner A., Ackermann L., Gabor B., Goddard R., Lehmann C. W., Mynott R., Stelzer F., Thiel O. R. (2001). Comparative Investigation
of Ruthenium-Based Metathesis Catalysts Bearing N-Heterocyclic Carbene
(NHC) Ligands. Chem. - Eur. J..

[53] Diesendruck C. E., Tzur E., Ben-Asuly A., Goldberg I., Straub B. F., Lemcoff N. G. (2009). Predicting the Cis–Trans
Dichloro Configuration
of Group 15–16 Chelated Ruthenium Olefin Metathesis Complexes:
A DFT and Experimental Study. Inorg. Chem..

[54] Pump E., Cavallo L., Slugovc C. (2015). A Theoretical
View on the Thermodynamic
Cis–Trans Equilibrium of Dihalo Ruthenium Olefin Metathesis
(Pre-)­Catalysts. Monatsh. Chem..

[55] Luo X., Kim Y. M., Lee M. J., Mejia E. B., Shi Y., Sottos N. R., Baur J. W., Xia Y. (2025). Multi-Generational
Frontal Curing and Chemical Recycling of Polydicyclopentadiene Thermosets. Adv. Mater..

