# Supplementary material for: Olefin Metathesis Catalyzed by a Latent Ruthenathiete Complex
Source: Organometallics. 2026 Mar 19;45(7):860–8. doi: 10.1021/acs.organomet.6c00021 (PMC13081106; doi:10.1021/acs.organomet.6c00021)
Supplement: Supplementary file 1 [file om6c00021_si_001.pdf]

Supporting Information

Olefin Metathesis Catalyzed by a Latent Ruthenathiete Complex

*Alec B. Pabarue[1], Tianqi Zhang[1], Mizhi Xu[1], John Bacsa[2], Will R. Gutekunst[1]**

[1]School of Chemistry and Biochemistry, Georgia Institute of Technology, 901 Atlantic Drive NW, Atlanta, Georgia 30332, United States.
[2]X-ray Crystallography Center, Department of Chemistry, Emory University, 1515 Dickey Drive, Atlanta, Georgia 30322, United States

* E-mail: willgute@gatech.edu

Contents

1. General information..............2
2. Synthetic Methods..............3
3. NMR Studies of Complex 10..............11
4. NMR Studies of Complex 11..............15
5. Crystallographic Data of Complex 11..............24
6. UV-Vis Spectrophotometry Data of Complexes 10 and 11..............50
7. General NMR and GPC Data of Intermediates and Metathesis Reactions..............51
8. References..............78

1. General information

All reactions, unless noted, were performed in oven-dried (120°C) glassware with magnetic stirring under an inert atmosphere of dry nitrogen. Dry dichloromethane (DCM) and tetrahydrofuran (THF) were obtained from a JC Meyer solvent purification system. Chloroform-*d* ($CDCl_3$), toluene-*d8* (Tol-d8) were purchased from Cambridge Isotope Laboratories, Inc. Unless otherwise stated, all reagents were purchased at the highest commercial quality and used without further purification. Analytical thin layer chromatography (TLC) was carried out using EM Science silica gel 60 F254 plates; visualization was accomplished with UV light (254 nm). Column chromatography was performed using Siliaflash F60. ^{1}H NMR spectra were recorded on a Bruker spectrometer (500 MHz). Chemical shifts were reported in ppm with the solvent resonance as the internal standard ($CDCl_3$, δ = 7.26; tol-d_8, δ = 2.08). Spectra were reported as follows: chemical shift (δ ppm), multiplicity (s = singlet, d = doublet, t = triplet, q = quartet, m = multiplet, comp = composite of magnetically non-equivalent protons, dd = doublet of doublets), coupling constants (Hz), integration and assignment. ^{13}C NMR spectra were collected on a Bruker instrument (400 and 500 MHz) with complete proton decoupling. Mass spectra (MS) were performed on an LC/MS (Agilent Technologies 1260 Infinity II/6120B Single Quadrupole). Melting points were measured on a MEL-TEMP II Laboratory Devices. Photoreactions were performed inside of a Rayonet RPR-100 photochemical reactor equipped with 300nm light bulbs with a fan to keep the reaction at 35 °C or for 390 nm irradiation, a photoreactor was equipped with two blue LED lamps (Kessil PR160L 390 nm, 75% intensity) approximately 11.0 cm away, and a fan to keep the reaction at 30 °C. 10-20mL microwave vials with 20mm aluminum seals were purchased from VWR (catalog number 89079-402). UV-Vis spectroscopy was performed on a Cary 5000 UV-Vis-NIR Spectrophotometer in 10mm quartz cuvettes purchased from Vernier (catalog number: ZCUV001X). Polymer samples were analyzed using a Tosoh EcoSEC HLC 8320GPC system with TSKgel SuperHZ-L columns eluting $CHCl_3$ containing 0.25% NEt_3 at a flow rate of 0.45 mL/min. All number-average molecular weights and dispersities were calculated from refractive index

chromatograms using PStQuick Mp-M polystyrene standards.

2. Synthetic Methods

Scheme S1. Synthesis of complex **10**. Aldehyde **S1** was synthesized according to a previously reported procedure.[1]

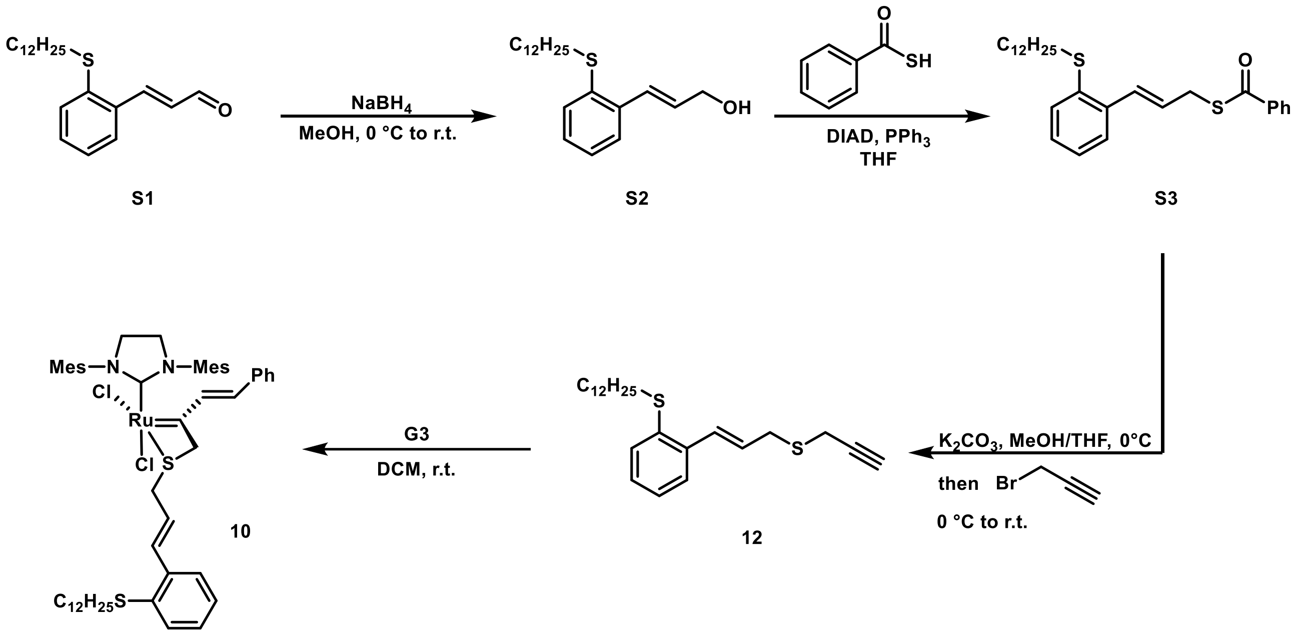

(E)-3-(2-(dodecylthio)phenyl)prop-2-en-1-ol (S2)

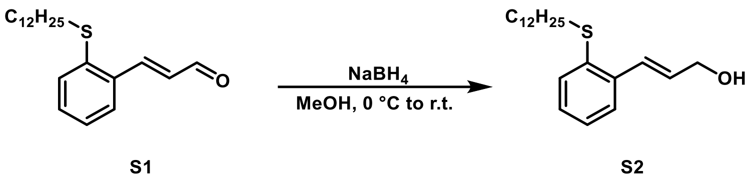

Cinnamaldehyde (**S1**, 1 mmol) was dissolved in 10 mL MeOH and cooled to 0°C. $NaBH_4$ (3 mmol) was added in one portion and allow the reaction to stir for 3 hours. The methanol was removed under reduced pressure, and the mixture was purified by column chromatography (9:1 hexanes/EtOAc) giving a white powder **S2** as a 94:6 E:Z ratio (93% yield). ^{1}H NMR (500 MHz, Chloroform-d) δ 7.49 (dd, J = 7.5, 1.7 Hz, 1H), 7.36 (dd, J = 7.6, 1.6 Hz, 1H), 7.21 (dtd, J = 18.1, 7.4, 1.6 Hz, 2H), 7.17 – 7.12 (m, 1H), 6.31 (dt, J = 15.8, 5.8 Hz, 1H), 4.38 (dd, J = 5.8, 1.6 Hz, 2H), 2.95 – 2.83 (m, 2H), 1.64 (p, J = 7.4 Hz, 4H), 1.43 (p, J = 6.9 Hz, 2H), 1.28 (s, 18H), 0.90 (t, J = 6.9 Hz, 3H). ^{13}C NMR (126 MHz, CDCl3) δ 137.26, 135.43, 130.50, 129.70, 128.89, 127.97, 126.38, 126.23, 63.92, 34.06, 31.93, 29.66, 29.64, 29.61, 29.52, 29.36, 29.20, 29.05, 28.89, 22.71, 14.14. (m/z [**S2**+Na+] = 357.2. Found: 358.1)

(E)-S-(3-(2-(dodecylthio)phenyl)allyl) benzothioate (S3)

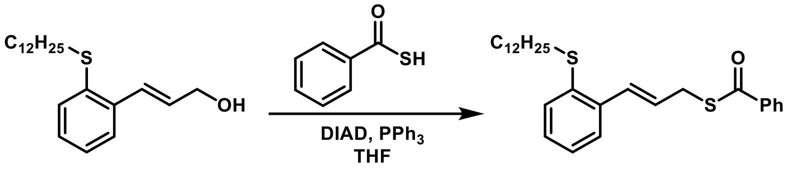

Diisoropyl azodicarboxylate (DIAD, 125.4 mg, 122 uL, 0.62 mmol, 2 eq) was dissolved in THF (2 mL) in a 20 mL vial then cooled to 0°C in an ice bath. Triphenylphospine (162.6 mg, 0.62 mmol, 2 eq) was dissolved in THF (1 mL) then added dropwise. The combined solutions were left to stir for 30 minutes resulting in a white precipitate. Alcohol **S2** and thiobenzoic acid were dissolved in 1 mL THF each and added in one portion. The reaction was stirred for 1 hour at 0°C then was left overnight at room temperature. The solvent was removed under reduced pressure then was purified via column chromatography (9:1 hexane:EtOAc) to give **S3** as a yellow oil (71% yield). ^{1}H NMR (500 MHz, Chloroform-*d*) δ 8.02 – 7.93 (m, 2H), 7.62 – 7.53 (m, 1H), 7.51 – 7.40 (m, 3H), 7.35 (dd, *J* = 7.3, 1.8 Hz, 1H), 7.23 – 7.11 (m, 3H), 6.25 – 6.12 (m, 1H), 3.95 (dd, *J* = 7.3, 1.3 Hz, 2H), 2.87 – 2.79 (m, 2H), 1.65 – 1.53 (m, 2H), 1.46 – 1.33 (m, 2H), 1.25 (d, *J* = 3.3 Hz, 16H), 0.88 (t, *J* = 6.9 Hz, 3H). ^{13}C NMR (101 MHz, $CDCl_3$) δ 191.34, 137.50, 136.99, 135.15, 133.41, 131.10, 130.39, 128.64, 127.93, 127.30, 126.43, 126.36, 126.26, 77.34, 77.03, 76.71, 34.35, 31.93, 31.82, 29.67, 29.65, 29.61, 29.53, 29.37, 29.20, 29.12, 28.87, 22.71, 14.14. (m/z [**S3**+K^+] = 393.2. Found: 393.3)

(E)-dodecyl(2-(3-(prop-2-yn-1-ylthio)prop-1-en-1-yl)phenyl)sulfane (12)

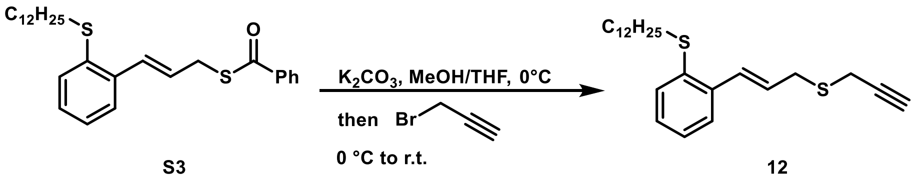

In a vial equipped with a stir bar, thiobenzoate **S3** (99.7 mg, 0.22 mmol) was dissolved in THF (2.5 mL) and MeOH (2.5 mL) and cooled to 0°C in an ice bath. Potassium carbonate (152mg, 1.1mmol, 5eq) was added to the solution and consumption of **S3** was monitored by TLC. Once **S3** was consumed, propargyl bromide (0.33 mL, 0.44 mmol, 2 eq) was added to the vial and the solution was stirred overnight. The solution was then concentrated, dissolved in THF (2 mL) and water (2 mL) with LiOH

(5 eq) and stirred for 4 hours. The mixture was extracted with DCM then the combined organics were washed with brine and dried with Mg_2SO_4 before being concentrated under reduced pressure and purified by column chromatography (97:3 hexane:EtOAc) to give **12** as a brown-yellow oil (78% yield). ^{1}H NMR (400 MHz, Chloroform-*d*) δ 7.49 (dt, *J* = 7.3, 2.4 Hz, 1H), 7.37 (dd, *J* = 7.2, 1.8 Hz, 1H), 7.21 (pd, *J* = 7.3, 1.7 Hz, 2H), 7.09 – 7.00 (m, 1H), 6.09 (dt, *J* = 15.3, 7.5 Hz, 1H), 3.56 – 3.49 (m, 2H), 3.26 (d, *J* = 2.6 Hz, 2H), 2.89 (q, *J* = 7.1 Hz, 2H), 2.30 (t, *J* = 2.6 Hz, 1H), 1.82 – 1.53 (m, 2H), 1.43 (t, *J* = 7.4 Hz, 2H), 1.28 (s, 16H), 0.90 (t, *J* = 6.7 Hz, 3H). 13C NMR (126 MHz, Chloroform-d) δ 194.01, 137.35, 135.22, 130.86, 130.74, 130.08, 129.90, 129.75, 127.94, 127.43, 127.32, 126.37, 126.35, 126.27, 124.93, 79.94, 71.23, 70.78, 42.28, 34.73, 34.13, 33.64, 31.93, 29.71, 29.66, 29.64, 29.60, 29.57, 29.53, 29.49, 29.36, 29.20, 29.10, 28.92, 28.89, 28.80, 22.70, 17.98, 14.14. m/z [**12** + H^+] = 389.2. Found 389.3.

Complex 10

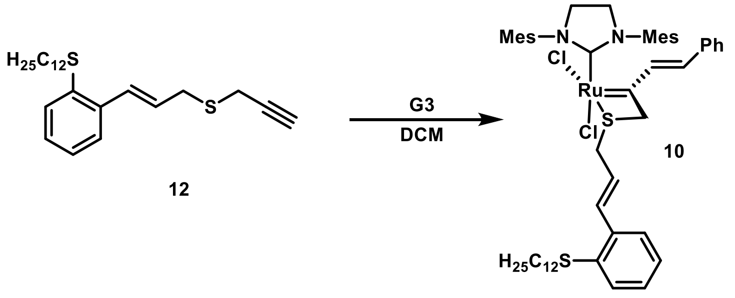

A dried 20 mL vial was loaded with a stir bar and Grubbs 3rd generation catalyst (**G3**, 8.9 mg, 0.012 mmol, 1 eq) and purged with nitrogen. In a separate nitrogen-purged vial, a 66mg/mL stock solution was prepared of (E)-dodecyl(2-(3-(prop-2-yn-1-ylthio)prop-1-en-1-yl)phenyl)sulfane (**12**) in degassed DCM. Sulfide stock solution (0.09 mL, 5.8 mg, 0.015 mmol, 1.3eq). was added to the vial containing **G3**. The reaction was allowed to stir for 20 minutes before being concentrated under reduced pressure. The black solid was dissolved in acetone and purified via column chromatography in acetone yielding the complex **10** as a green solid (49% yield). ^{1}H NMR (500 MHz, Chloroform-*d*) δ 8.37 (d, *J* = 16.4 Hz, 1H), 7.54 – 7.49 (m, 3H), 7.45 (t, *J* = 7.3 Hz, 1H), 7.39 (dd, *J* = 7.8, 1.4 Hz, 1H), 7.31 (d, *J* = 7.5 Hz, 2H), 7.29 (d, *J* = 1.8 Hz, 1H), 7.27 – 7.21 (m, 1H), 7.09 (dd, *J* = 8.8, 6.9 Hz, 2H), 6.80 (d, *J* = 2.1 Hz, 1H), 6.73 (d, *J* = 2.0 Hz, 1H), 6.44 – 6.36 (m, 2H), 6.14 (ddd, *J* = 15.4, 9.8,

5.4 Hz, 1H), 4.31 (td, J = 11.4, 9.0 Hz, 1H), 4.19 (d, J = 16.2 Hz, 1H), 4.17 – 4.02 (m, 3H), 3.89 (d, J = 16.1 Hz, 1H), 3.66 (dd, J = 12.5, 9.9 Hz, 1H), 3.55 – 3.48 (m, 1H), 2.89 (t, J = 7.4 Hz, 2H), 2.64 (s, 3H), 2.48 (s, 3H), 2.40 (s, 3H), 2.31 (s, 3H), 2.29 (s, 3H), 2.20 (d, J = 3.1 Hz, 3H), 1.78 (d, J = 20.2 Hz, 4H), 1.68 – 1.60 (m, 5H), 1.43 (p, J = 7.2 Hz, 3H), 1.27 (d, J = 8.0 Hz, 16H), 0.89 (t, J = 6.9 Hz, 4H). ^{13}C NMR (176 MHz, $CDCl_3$) δ 273.32, 216.20, 207.01, 140.27, 139.90, 139.33, 138.67, 138.34, 137.11, 136.78, 136.10, 135.56, 135.51, 135.20, 132.58, 131.11, 130.83, 130.56, 130.23, 129.64, 129.33, 129.29, 128.47, 128.41, 128.33, 128.04, 127.42, 126.50, 126.23, 124.40, 117.92, 77.22, 77.18, 77.13, 77.03, 76.85, 74.72, 53.45, 51.96, 51.22, 38.11, 34.72, 34.12, 31.92, 30.96, 29.71, 29.65, 29.62, 29.53, 29.36, 29.21, 29.14, 29.09, 28.94, 22.70, 21.26, 20.56, 20.16, 19.31, 18.89, 18.01, 14.14. (m/z [**complex 10**+ACN] = 998.2, found: 998.4)

[(2-Propyn-1-ylthio)methyl]benzene (15)

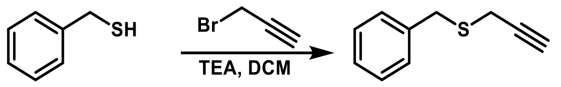

15

[(2-Propyn-1-ylthio)methyl]benzene was synthesized using a modified literature procedure.[2] Benzylmercaptan (1.90 mL, 16.2 mmol) was dissolved in DCM (10 mL) before adding propargyl bromide (80% in solution in PhMe, 2.23 mL, 20 mmol). The reaction mixture was cooled in an ice/water bath and TEA was added (2.51 mL, 18 mmol) dropwise. The reaction was left stirring at room temperature overnight. H_2O was added to solubilize the salts formed. The mixture was added to a separatory funnel and 10 mL of DCM was added. The organic layer was washed twice with H_2O and dried over $MgSO_4$ before being concentrated under reduced pressure. The crude oil was purified by column chromatography, eluted with 9:1 hexane: EtOAc and dried, giving **15** as a yellow oil (72% yield). ^{1}H NMR (500 MHz, Chloroform-*d*) δ 7.37 – 7.27 (m, 5H), 3.88 (s, 2H), 3.08 (d, J = 2.6 Hz, 2H), 2.30 (t, J = 2.6 Hz, 1H). NMR data matches the literature.[3]

Complex 11

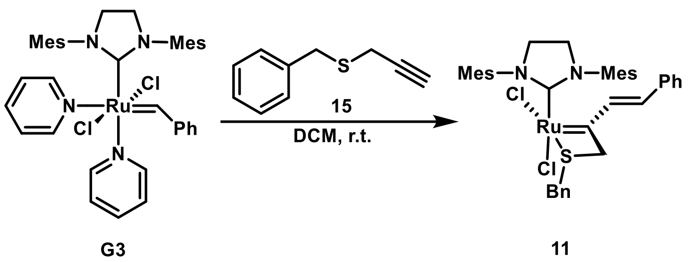

A dried 20mL vial was loaded with a stir bar and Grubbs 3rd generation catalyst (**G3**, 69.8 mg, 0.09 mmol, 1 eq) and purged with nitrogen. In a separate nitrogen-purged vial, a 60 mg/mL stock solution was prepared of [(2-propyn-1-ylthio)methyl]benzene (**15**) in degassed DCM. Degassed DCM (8.3 mL) was added to the vial containing G3 followed by addition of the sulfide stock solution (0.49 mL, 29 mg, 0.18 mmol, 2 eq). The reaction was allowed to stir for 30 minutes before being concentrated under reduced pressure. The black solid was dissolved in acetone and purified via column chromatography in acetone yielding the complex **11** as a green solid (50% yield). ^{1}H NMR (500 MHz, Chloroform-d) δ 8.35 (d, J = 16.4 Hz, 1H), 7.50 (d, J = 7.6 Hz, 1H), 7.44 (t, J = 7.4 Hz, 1H), 7.36 (m, 2H), 7.07 (s, 1H), 6.73 (d, J = 17.3 Hz, 1H), 6.39 – 6.31 (m, 1H), 4.32 – 4.20 (m, 1H), 4.20 – 4.14 (m, 1H), 4.13 – 3.99 (m, 2H), 3.78 (dd, J = 14.3, 8.5 Hz, 1H), 2.62 (s, 2H), 2.44 (s, 2H), 2.34 (s, 2H), 2.24 (s, 2H), 2.13 (s, 2H), 1.75 (s, 2H). ^{13}C NMR (176 MHz, $CDCl_3$) δ 273.85, 216.18, 173.34, 172.96, 170.97, 170.53, 170.12, 140.09, 139.80, 139.33, 138.64, 138.36, 137.06, 136.01, 135.57, 135.24, 135.21, 130.79, 130.65, 130.56, 130.30, 130.20, 129.49, 129.42, 129.36, 128.75, 128.45, 128.04, 127.79, 127.74, 117.85, 77.22, 77.04, 76.85, 75.27, 74.45, 69.12, 68.72, 62.33, 62.30, 61.99, 51.96, 51.19, 38.19, 34.19, 34.13, 34.04, 31.93, 31.75, 29.70, 29.68, 29.66, 29.61, 29.54, 29.52, 29.43, 29.37, 29.33, 29.24, 29.21, 29.10, 29.04, 25.33, 25.28, 24.89, 24.85, 22.70, 22.59, 21.31, 20.90, 20.71, 20.54, 20.15, 18.89, 18.80, 17.95, 14.13, 14.08. (m/z [**complex 11**-Cl] = 696.2, found: 697.0, [**complex 11**-Cl+Na+] = 719.2., found: 719.5)

N,N-Diallyl-4-methylbenzenesulfonamide (18a)

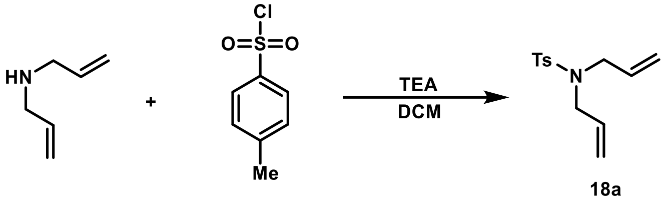

18a

Magnetically stirred solutions of *N,N*-diallylamine (2.2 g, 2.8 mL, 22.9 mmol, 1.2 eq) and tosyl chloride (3.6 g, 19.1 mmol, 1 eq) were prepared in dichloromethane (50 mL) in a 200 mL flask. The solution was treated with triethylamine (3.9 g, 5.3 mL, 38.2 mmol, 2 eq) and stirred for 12 hours. The reaction was acidified with 1M hydrochloric acid was then added to the reaction mixture and extracted 3 times with DCM (100 mL). Na_2SO_4 was added to the combined organics before being filtered and concentrated under reduced pressure to give **18a** (98% yield). ^{1}H NMR (400 MHz, Chloroform-*d*) δ 7.74 – 7.66 (m, 2H), 7.29 (d, *J* = 8.0 Hz, 2H), 5.61 (ddt, *J* = 17.5, 9.8, 6.3 Hz, 2H), 5.21 – 5.05 (m, 4H), 3.80 (dd, *J* = 6.3, 1.3 Hz, 4H), 2.43 (s, 3H). NMR data matches the literature.[4]

N-allyl-4-methylbenzenesulfonamide

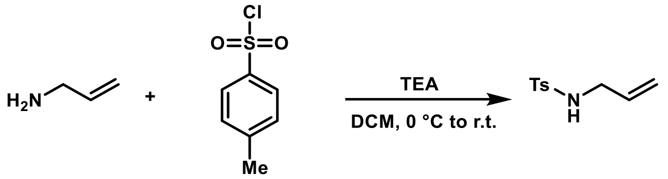

Tosyl chloride (13.4g, 70.3mmol, 1eq) was dissolved in DCM (32 mL) in a 100 mL flask were cooled to 0°C in an ice bath. Allylamine (4.8 g, 6.3 mL, 84.3 mmol, 1.2 eq) was added dropwise to the magnetically stirred solution. The reaction warmed to room temperature and stirred overnight. The reaction was acidified with 1M hydrochloric acid was then added to the reaction mixture and extracted 3 times with DCM (50 mL). Na_2SO_4 was added to the combined organics before being filtered and concentrated under reduced pressure to give *N*-allyl-4-methylbenzenesulfonamide (91% yield). ^{1}H NMR (500 MHz, Chloroform-*d*) δ 7.80 – 7.69 (m, 2H), 7.31 (d, *J* = 8.1 Hz, 2H), 5.72 (ddt, *J* = 17.2, 10.3, 5.8 Hz, 1H), 5.17 (dq, *J* = 17.0, 1.5 Hz, 1H), 5.10 (dp, *J* = 10.1, 1.2 Hz, 1H), 4.46 (s, 1H), 3.59 (tt, *J* = 6.1, 1.6 Hz, 2H), 2.43 (s, 3H). NMR data matches the literature.[4]

***N*-Allyl-4-methyl-*N*-(2-methylallyl)benzenesulfonamide (20a)**

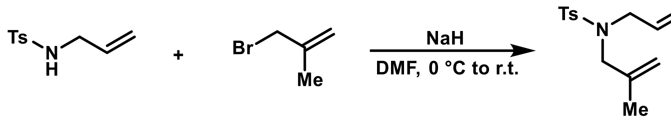

20a

A magnetically stirred solution of *N*-allyl-4-methylbenzenesulfonamide (3.18 g, 15.0 mmol, 1.2 eq) and 3-bromo-2-methylpropene (1.7 g, 12.5 mmol, 1 eq) in DMF (30 mL) in 100 mL flask was cooled to 0°C in an ice bath. The solution was treated with sodium hydride (450 mg, 18.75 mmol, 1.5 eq) and allowed to stir for 16 hours after warming to room temperature. The reaction was acidified with 1M hydrochloric acid was then added to the reaction mixture and extracted 3 times with DCM (50mL). Na_2SO_4 was added to the combined organics before being filtered and concentrated under reduced pressure. The crude oil was purified by column chromatography, eluted with 2:1 hexane: EtOAc and dried, giving a **20a** as pale-yellow oil (36% yield). ^{1}H NMR (500 MHz, Chloroform-*d*) δ 7.73 – 7.67 (m, 2H), 7.29 (d, *J* = 8.1 Hz, 2H), 5.52 (ddt, *J* = 18.0, 9.5, 6.6 Hz, 1H), 5.13 – 5.04 (m, 2H), 4.92 – 4.80 (m, 2H), 3.77 (dt, *J* = 6.5, 1.3 Hz, 2H), 3.70 (s, 2H), 2.43 (s, 3H). NMR data matches the literature.[4]

General procedure for Ring-Opening Metathesis Polymerization

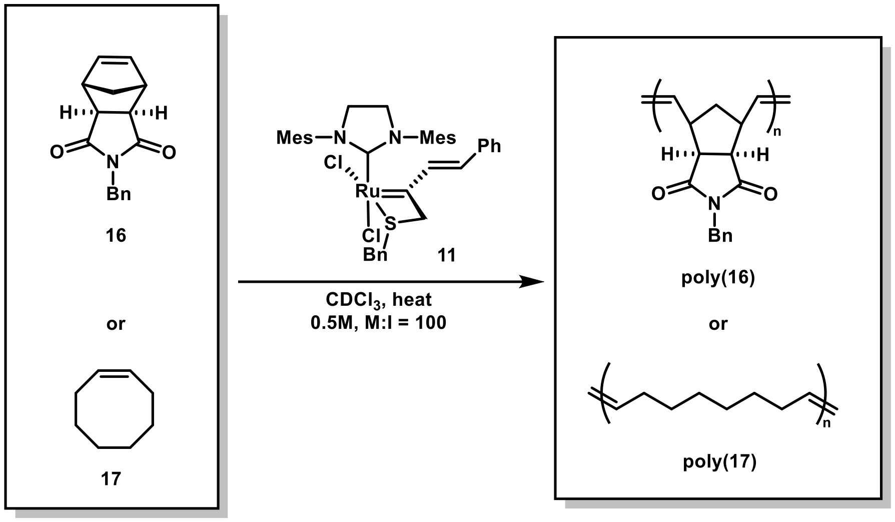

Septum-capped NMR tubes or microwave vials were loaded with *N*-benzyl norbornene imide (**16**, 78 mg, 0.3 mmol) or cyclooctene (**17**, 37 mg, 0.3 mmol) and purged with nitrogen before being dissolved in $CDCl_3$. In a separate vial, a stock solution of catalyst **11** was prepared in degassed $CDCl_3$. Monomer solutions were pre-heated to the desired temperature for 5 minutes (35 °C, 50 °C, 80 °C or 100 °C). The stock solution of **11** (2.25 mg, 0.003 mmol) was added to the NMR tube to make a 0.5M solution with respect to **16** or **17**. The NMR tube or microwave vial was heated at the desired temperature for 100 min.

General procedure for Ring-Closing Metathesis/Cross metathesis reactions

In a microwave vial equipped with a stir bar, substrate **18a** (or **19a**, **20a**, **21a, 22a**) was added (0.1 mmol). The vial was sealed and purged with nitrogen. In a separate vial, a stock solution of catalyst **11** was prepared in degassed solvent ($CDCl_3$, DCE, or tol-d_8). Solvent was added to the high-pressure vial followed by the stock solution of catalyst **11** (0.005 mmol) to make a 0.1M solution. The vial was sealed with electrical tape followed by heating to the appropriate temperature (35 °C, 60 °C, or 100 °C) in an oil bath. After the desired amount of time, the vial was transferred to an ice bath for 2 minutes. Then, the cap of the sealed vial was removed, and the contents of the vial were transferred to an NMR tube for analysis.

Procedure for Ring-Closing Metathesis reaction of S1 in photochemical reactor

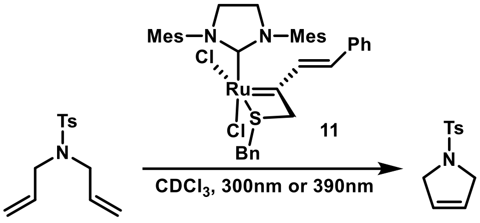

18a **18b**

In an 8mL vial, substrate **18a** was added (21.8 mg, 0.09 mmol). The vial was sealed and purged with nitrogen. In a separate vial, catalyst **11** (13.7 mg) was weighed out and dissolved in degassed $CDCl_3$ (1 mL). $CDCl_3$ After dissolving **18a** in $CDCl_3$ (636 µL), the stock solution of **11** was added to the vial (0.23 µL, 3.17 mg, 0.004 mmol) to make a 0.1M solution with respect to **18a**. The vial was transferred to a Rayonet RPR-100, and the sample was irradiated with 300nm UV light for 1 hour. For 390 nm two blue Kessil LED lamps were used as described in the general methods for 1 hour. Then the solution was transferred to an NMR tube and conversion was analyzed by ^{1}H NMR.

3. NMR Studies of Complex 10

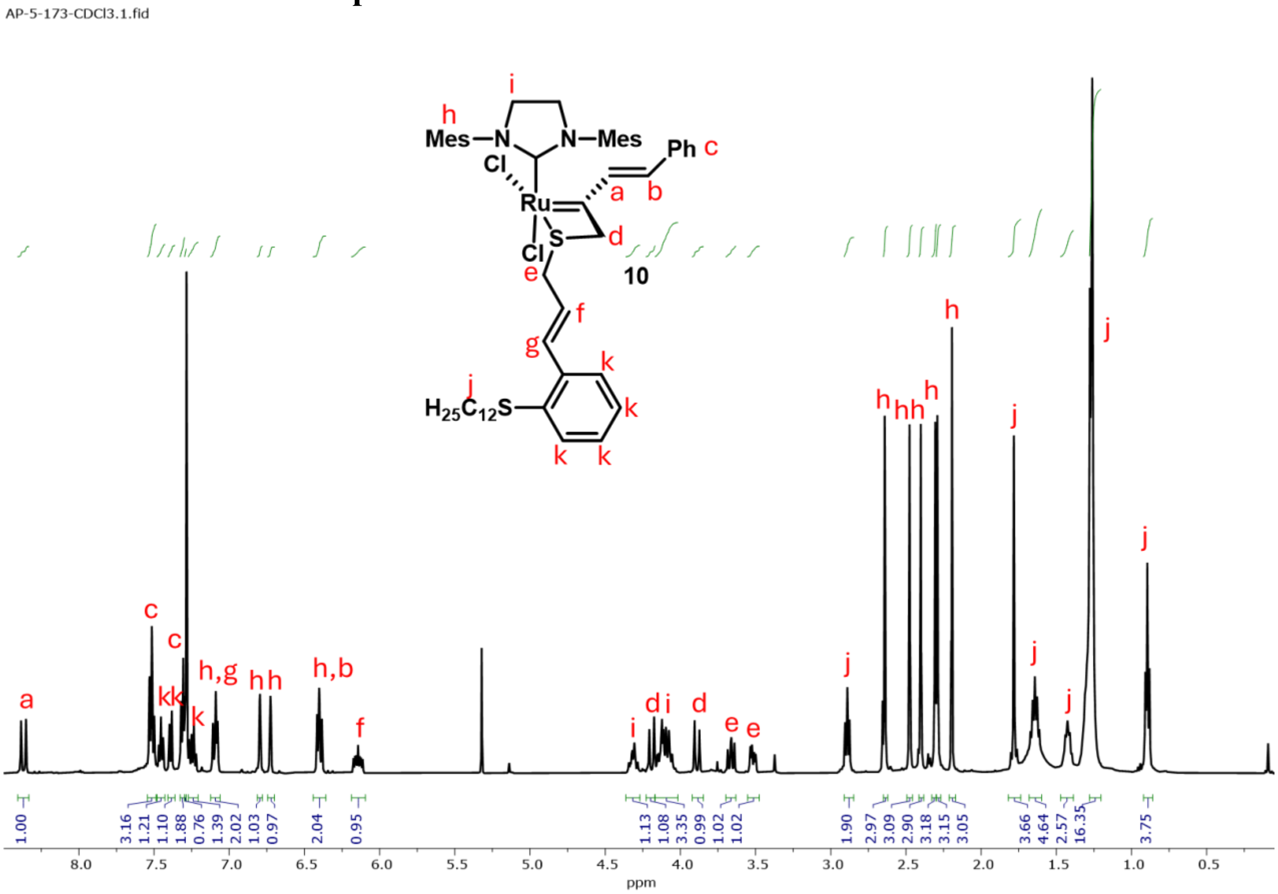

Figure S1. ^{1}H NMR of complex **10** in $CDCl_3$ with all proton assignments.

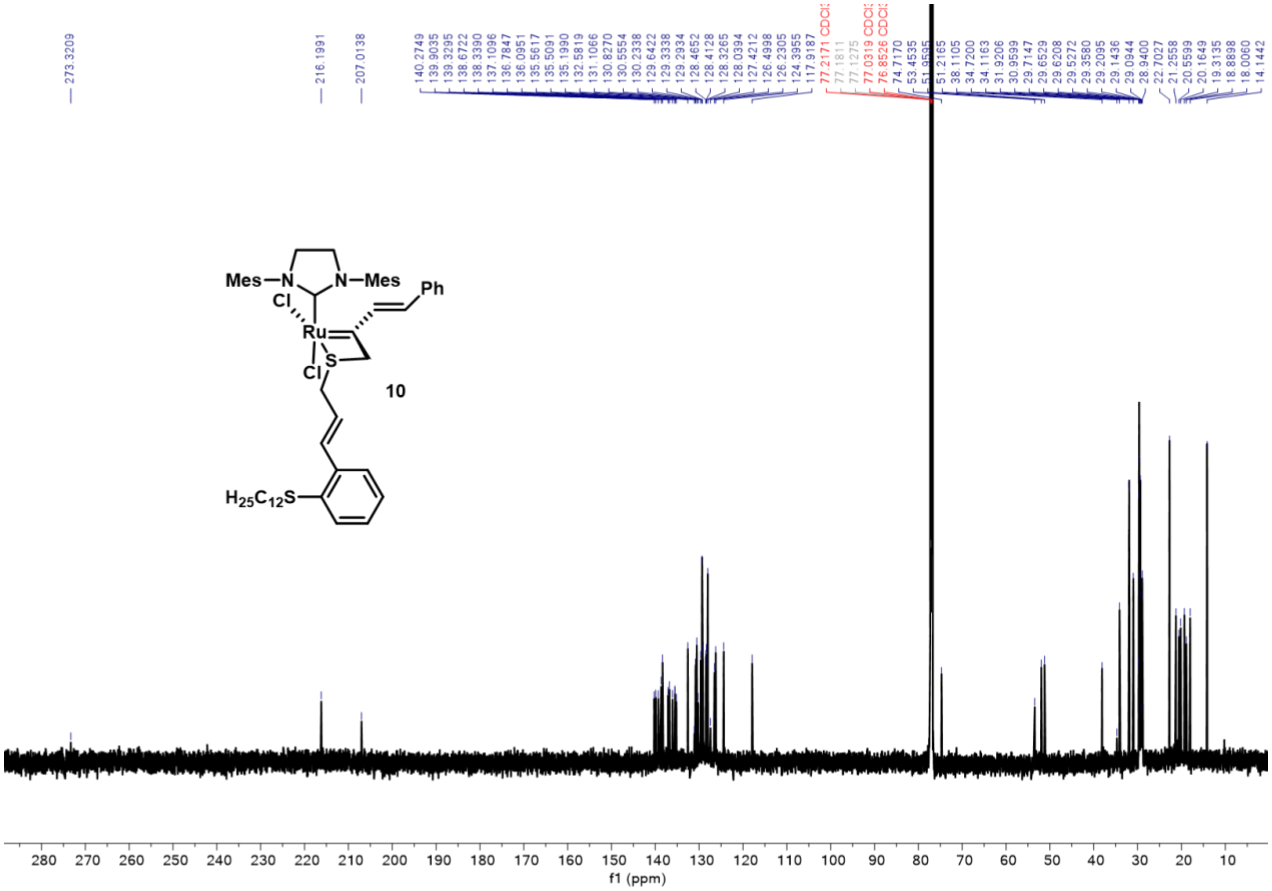

Figure S2. ^{13}C NMR of complex **10** in $CDCl_3$.

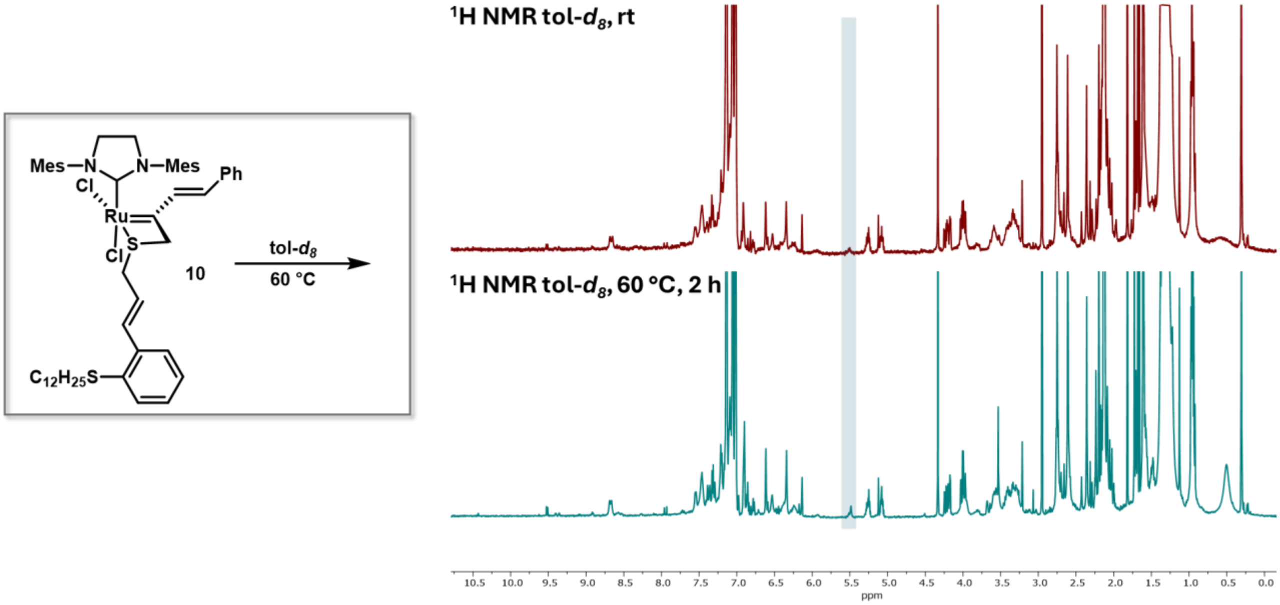

Figure S3. ^{1}H NMR of complex **10** in tol-d_8 before (red) and after (blue) heating at 60 °C for 2 h.

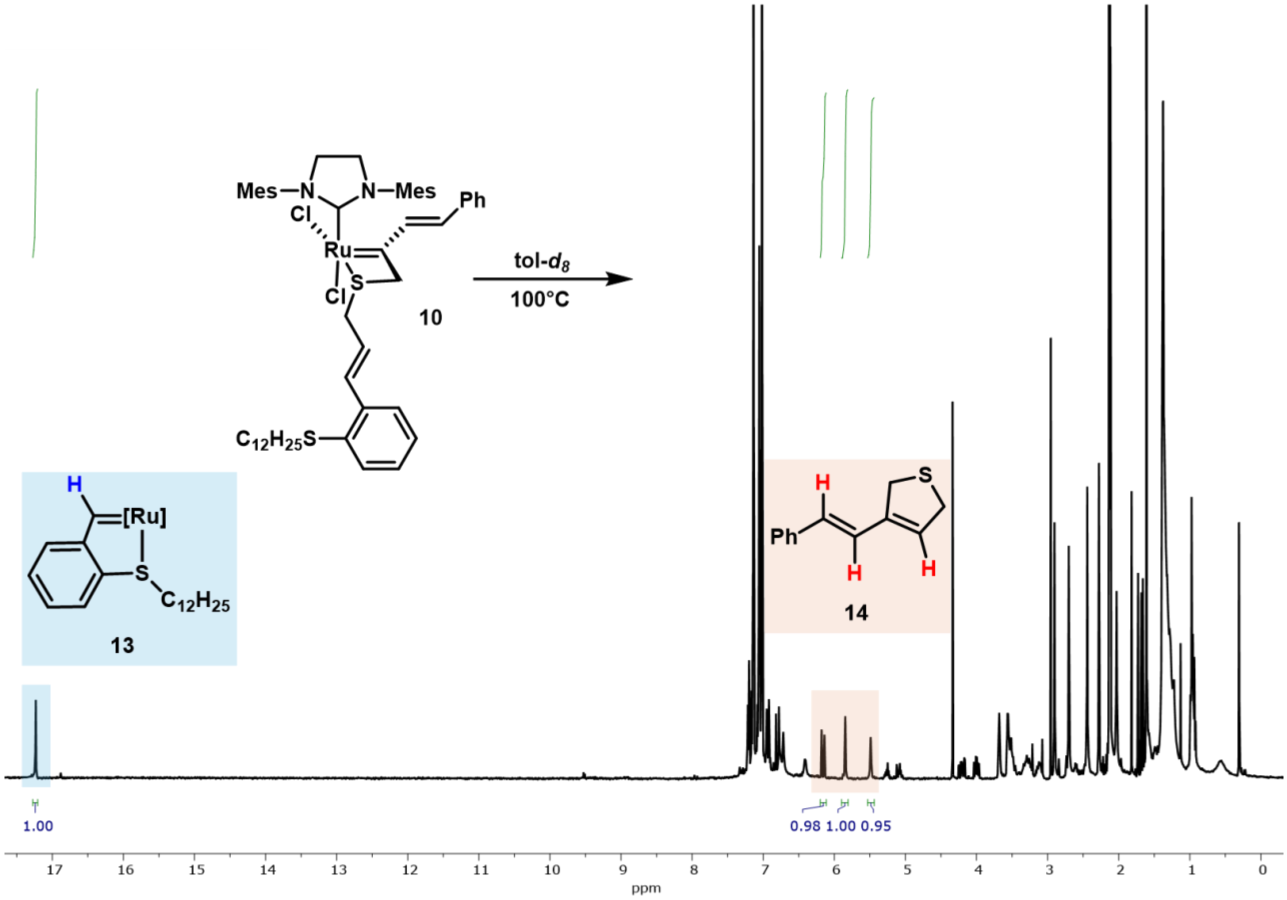

Figure S4. ^{1}H NMR of complex **10** in tol-d_8 after heating at 100 °C for 3 h.

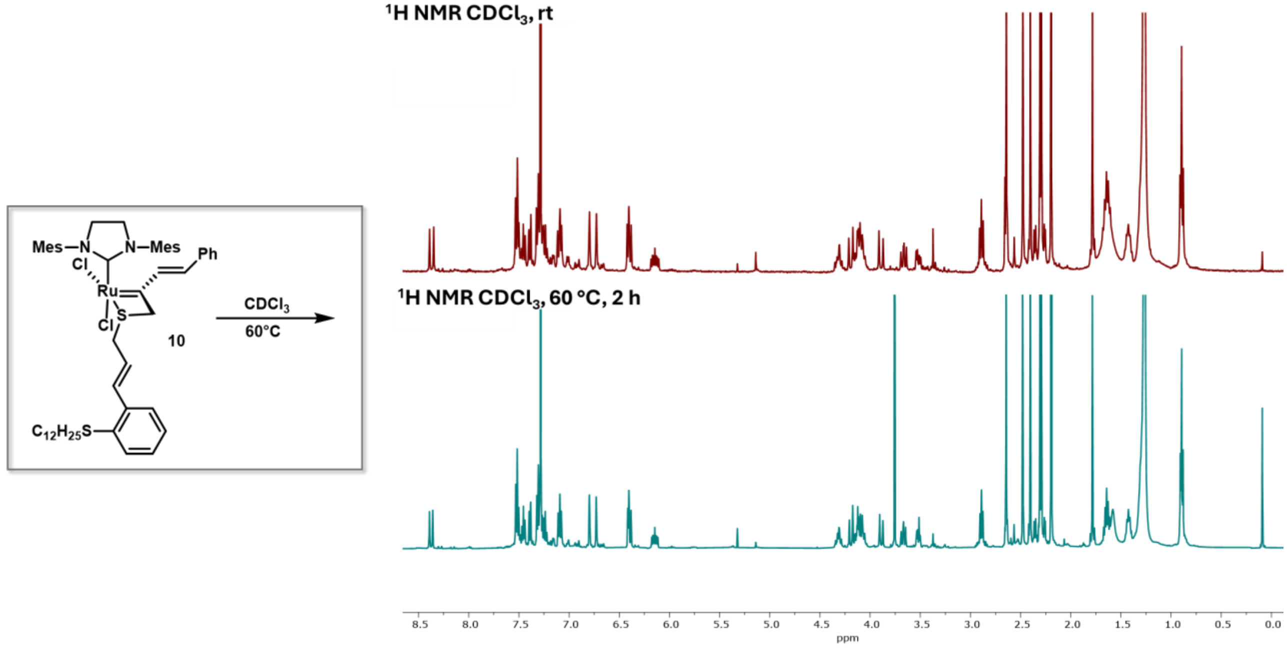

Figure S5. ^{1}H NMR of complex **10** in $CDCl_3$ before (red) and after (blue) heating at 60 °C for 2 h.

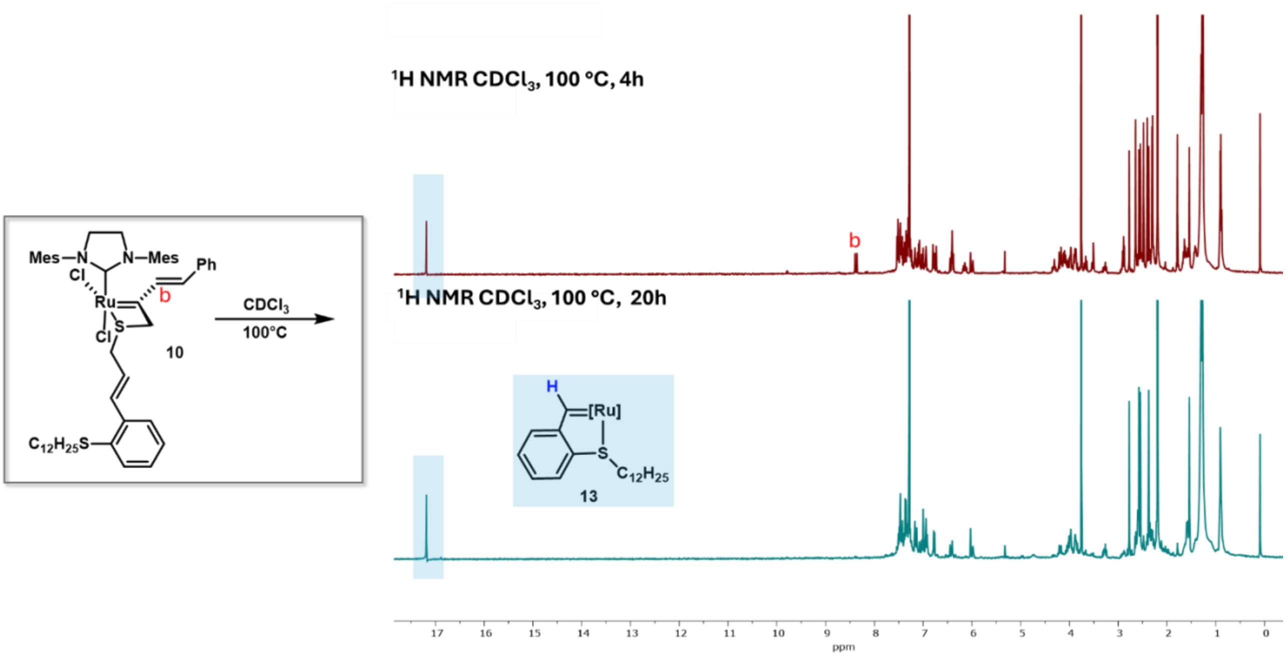

Figure S6. ^{1}H NMR of complex **10** in $CDCl_3$ after being heated at 100 °C for 4 hours (red, 45% conversion) and after being heated at 100 °C for 20 h (blue, full conversion).

4. NMR Studies of Complex 11

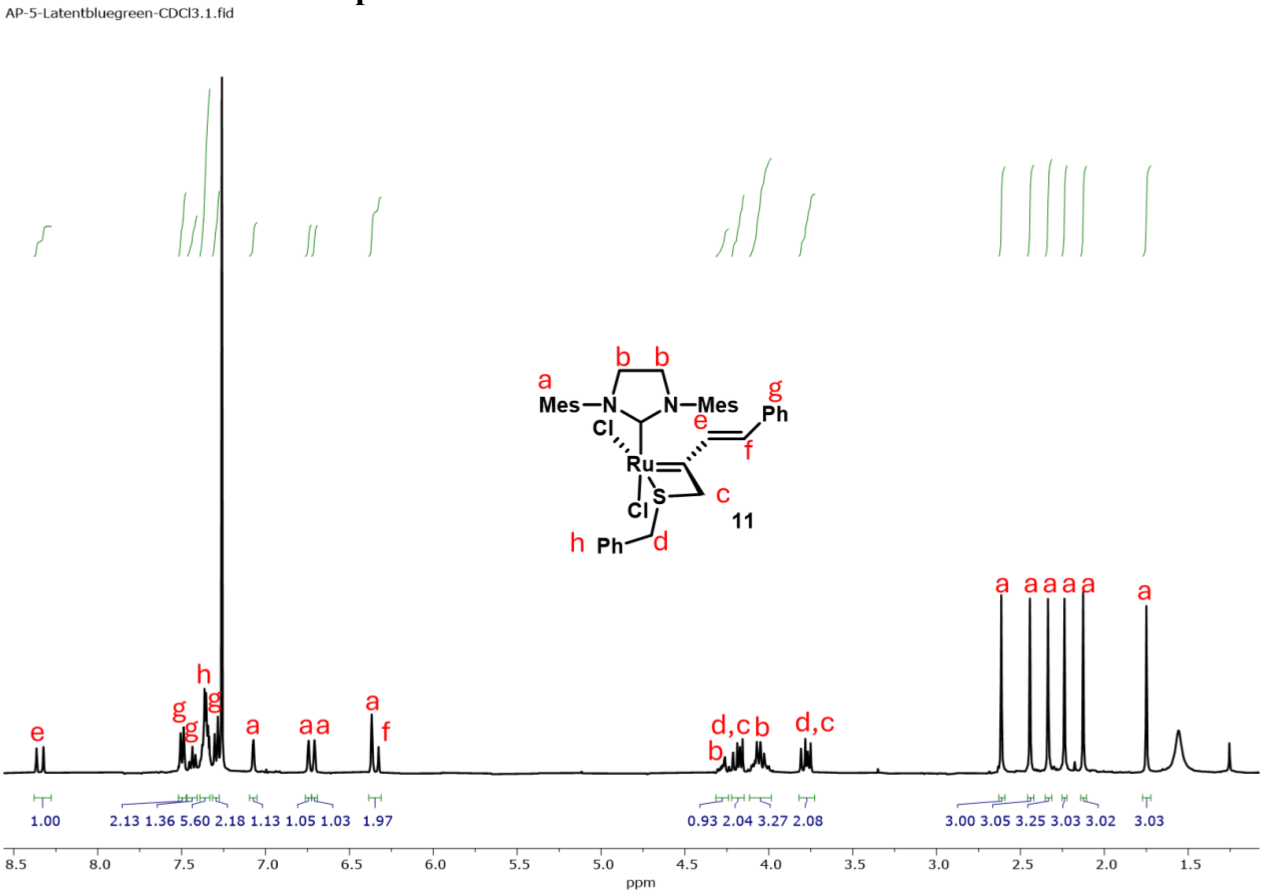

Figure S7. ^{1}H NMR of complex **11** in $CDCl_3$ with proton assignments.

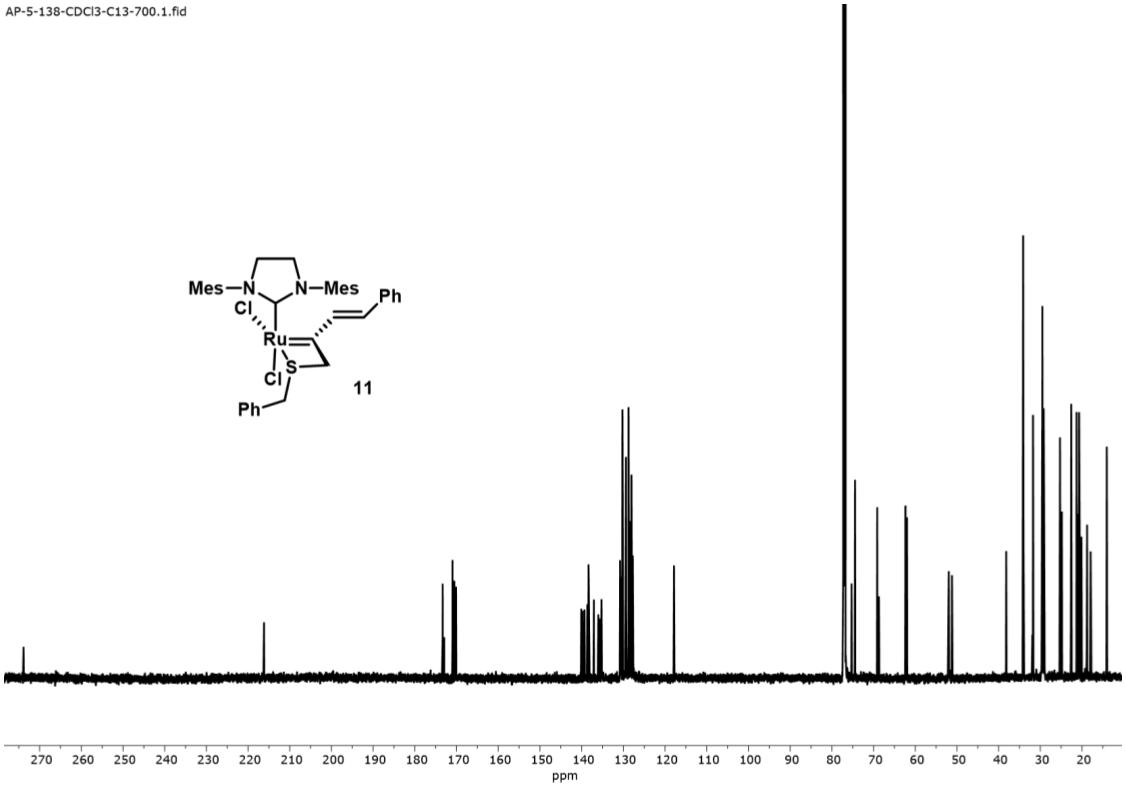

Figure S8. ^{13}C NMR of complex **11** in $CDCl_3$.

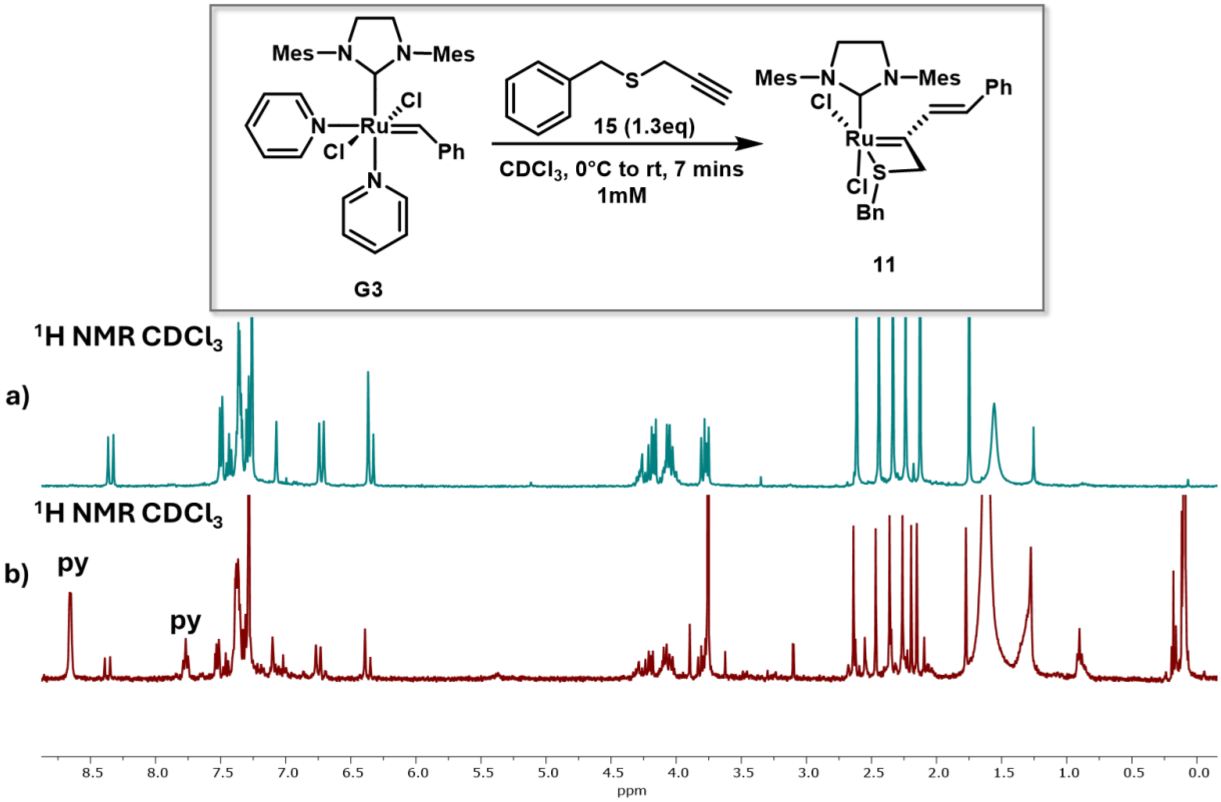

Figure S9. a) ^{1}H NMR of purified complex **11** in $CDCl_3$ and b) ^{1}H NMR of the reaction mixture of **G3** and **3** after 7 minutes at 1mM.

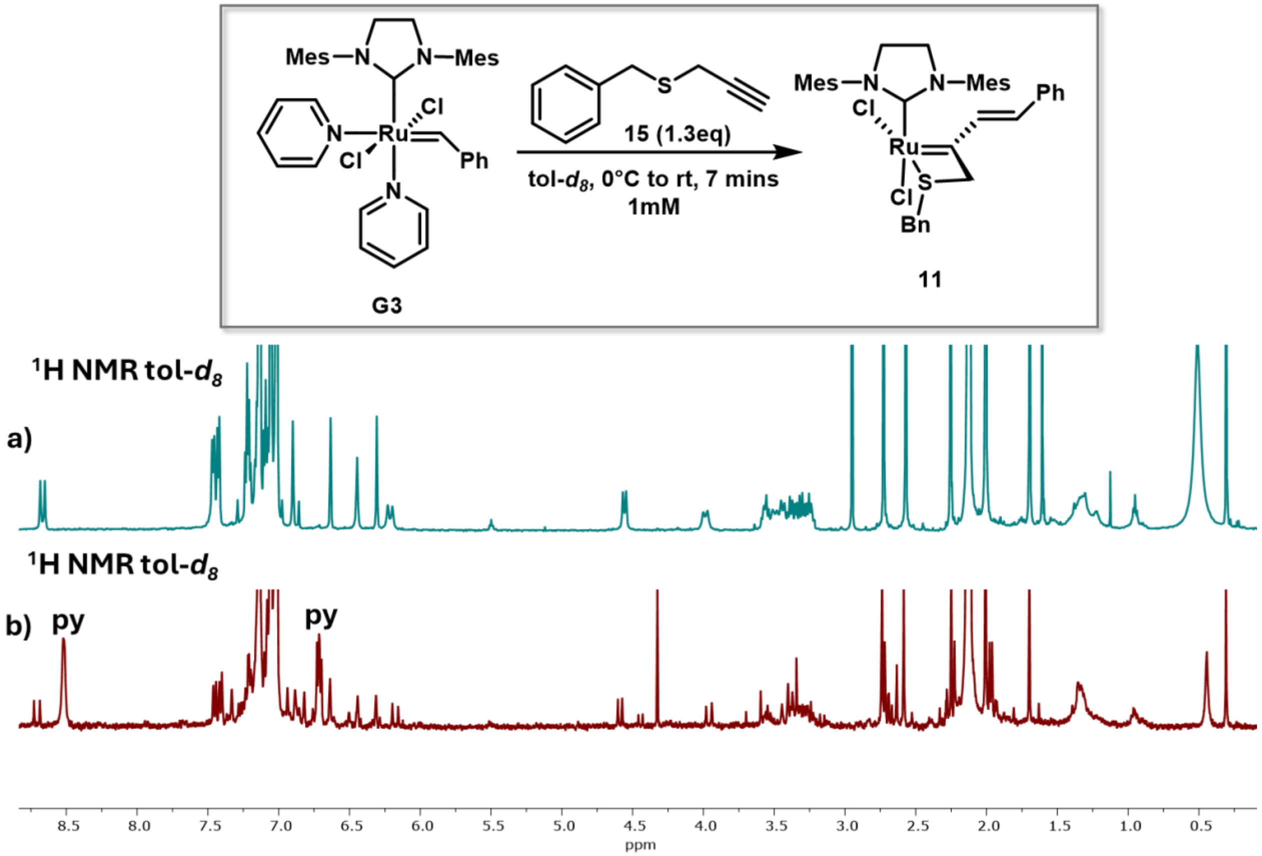

Figure S10. a) ^{1}H NMR of purified complex **11** in tol-d_8 and b) ^{1}H NMR of the reaction mixture of **G3** and **3** after 7 minutes at 1mM.

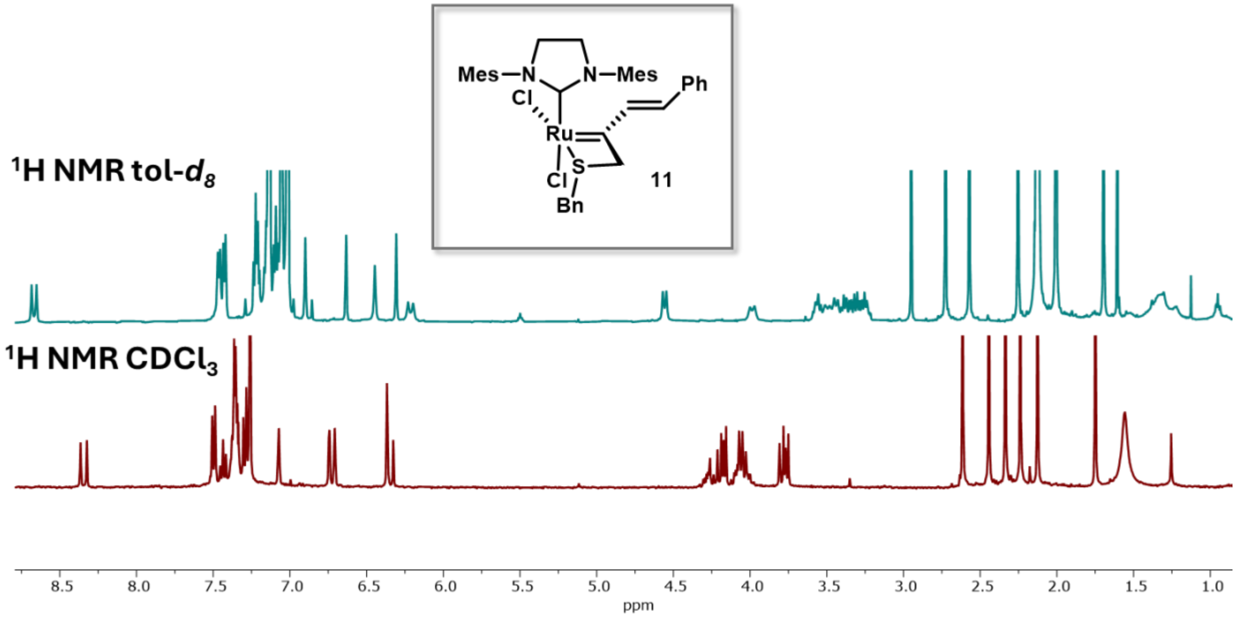

Figure S11. ^{1}H NMR of complex **11** in toluene-d_8 (blue) and $CDCl_3$ (red).

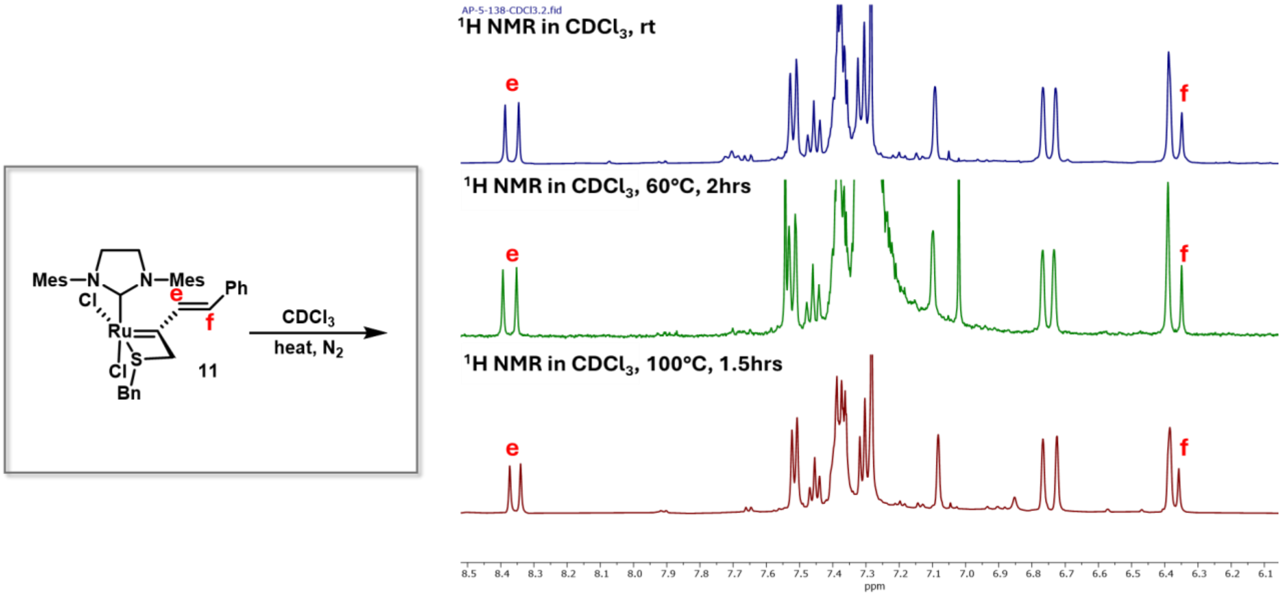

Figure S12. ^{1}H NMR of olefin peaks e and f of catalyst **11** in $CDCl_3$ before and after heating at 60 °C for 2 h and heating at 100 °C for 1.5 h.

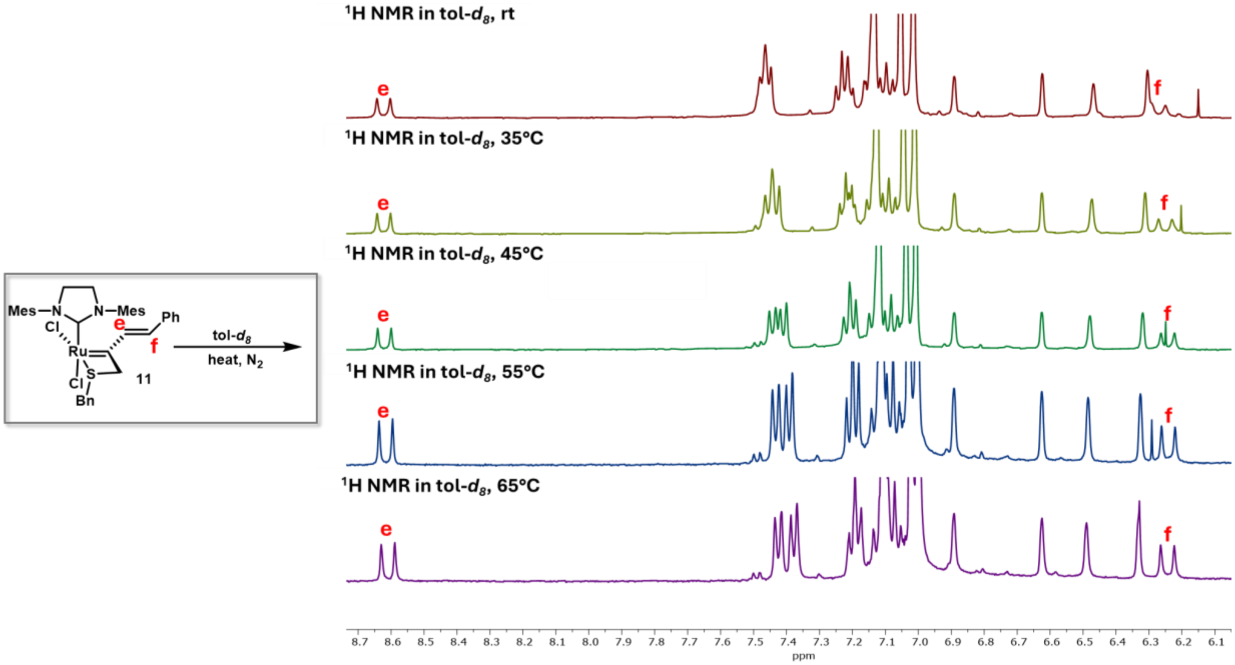

Figure S13. ^{1}H NMR of olefin peaks e and f of catalyst **11** in tol-d_8 at various temperatures.

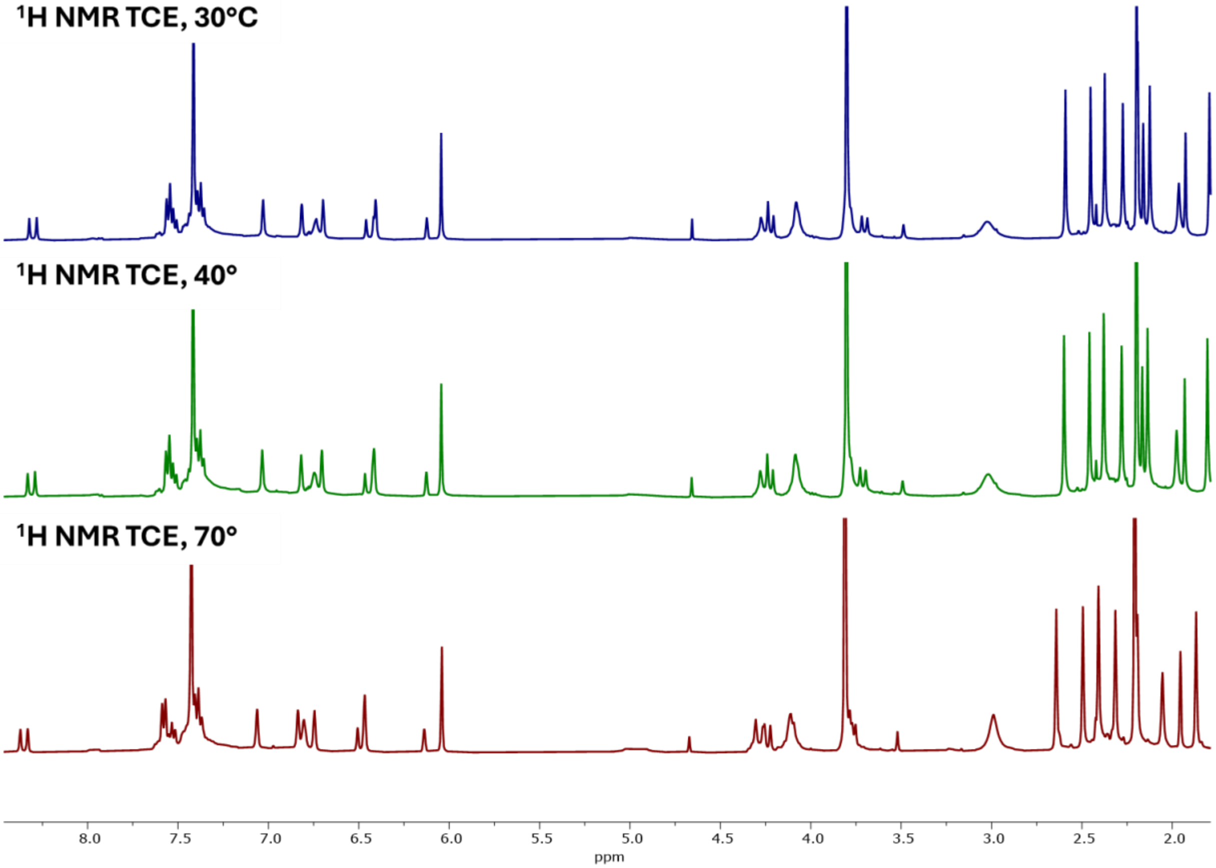

Figure S14. Variable-temperature ^{1}H NMR of **11** in TCE-d_2.

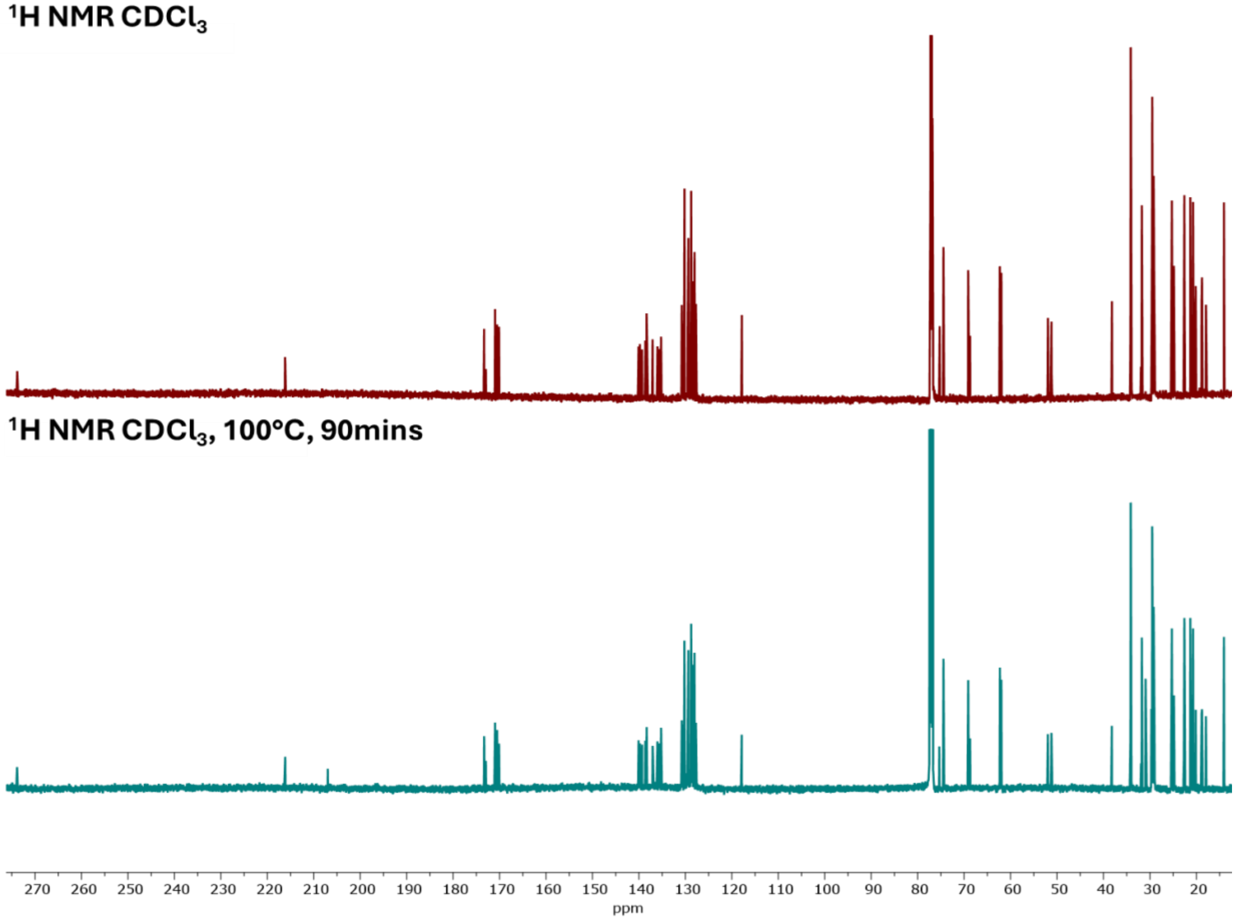

Figure S15. ^{13}C NMR of complex **11** in $CDCl_3$, of catalyst before (top) and after heating at 100 °C for 1.5 h (bottom).

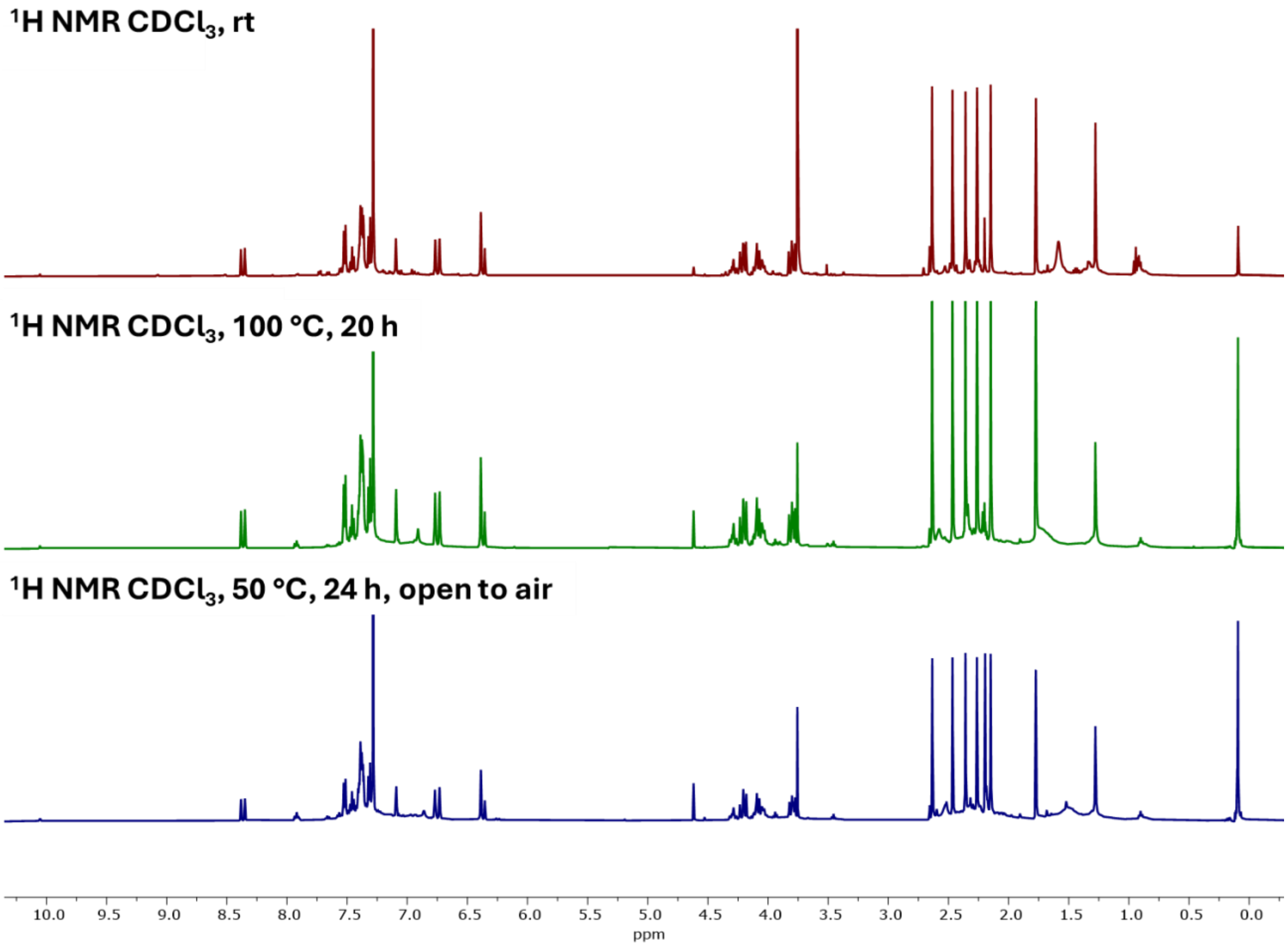

Figure S16. ^{1}H NMR of complex **11** in $CDCl_3$ after heating at 100 °C for 1 h under nitrogen (red), after heating at 100 °C for 20 h (green), and after being heated for 24 h at 50 °C open to air (blue).

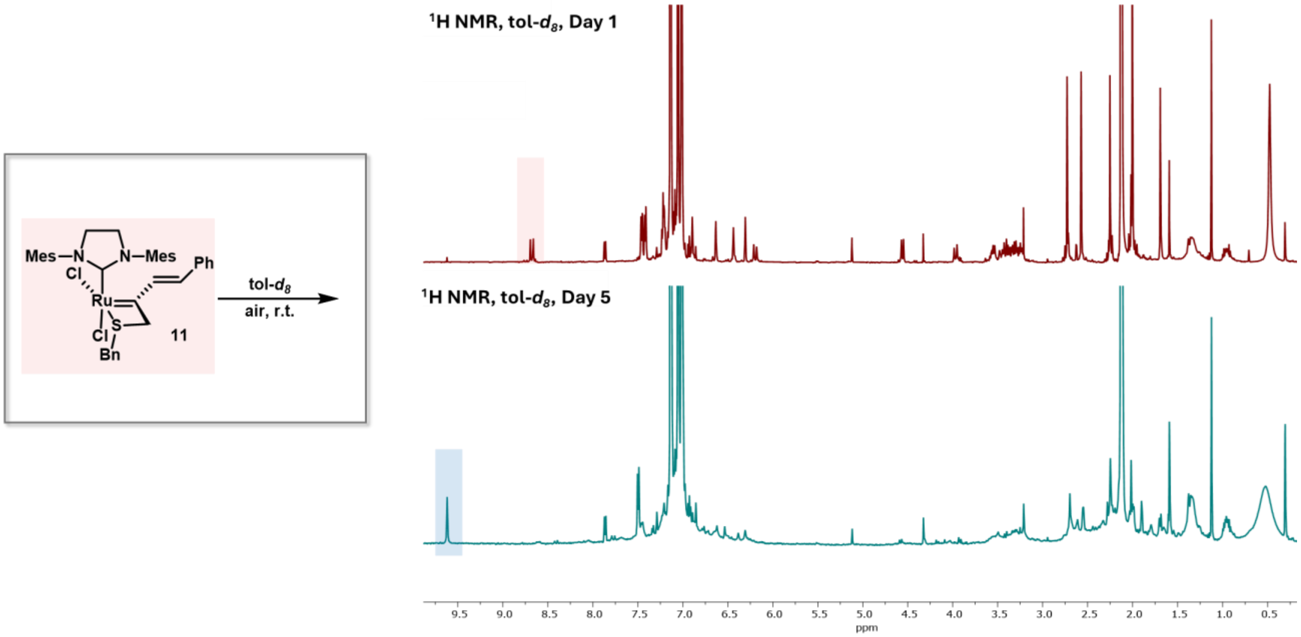

Figure S17. ^{1}H NMR of complex **11** in tol-d_8 after 5 days at room temperature. Aldehyde degradation product is highlighted in blue.

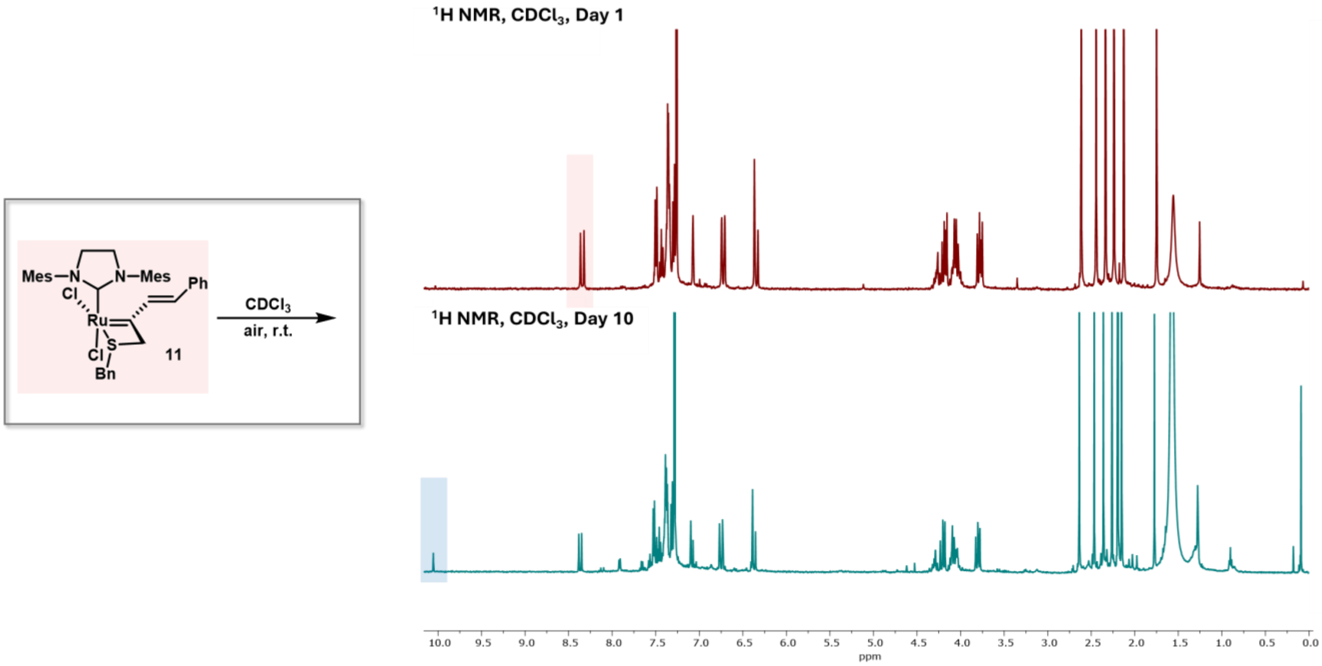

Figure S18. ^{1}H NMR of complex **11** in $CDCl_3$ after 10 days at room temperature. Aldehyde degradation products are highlighted in blue.

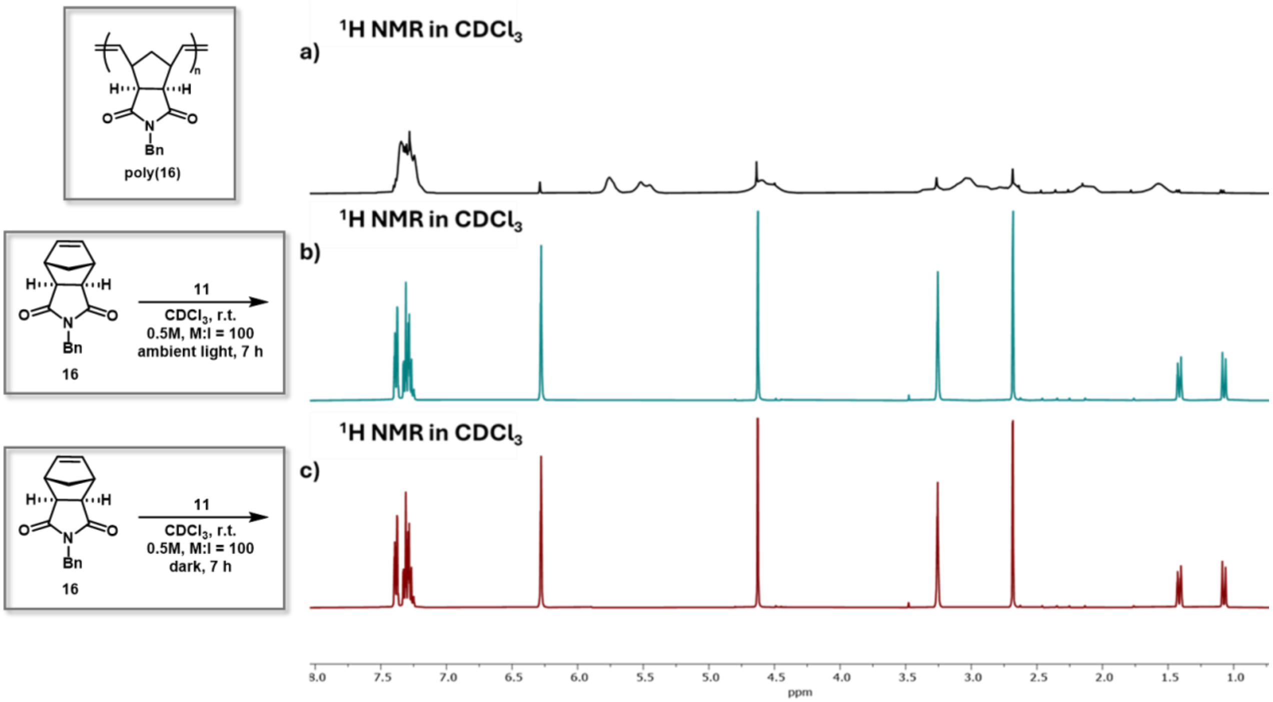

Figure S19. a) ^{1}H NMR of poly(**16**) after being heated with **11** at 35°C, 0.5M for 100 min (monomer to initiator ratio = 100). b) ^{1}H NMR of complex **11** with norbornene imide **16** after being kept under ambient light (rt, 0.5M, 7 hours). c) ^{1}H NMR of complex **11** with norbornene imide **16** after being kept in the dark (rt, 0.5M, 7 hours).

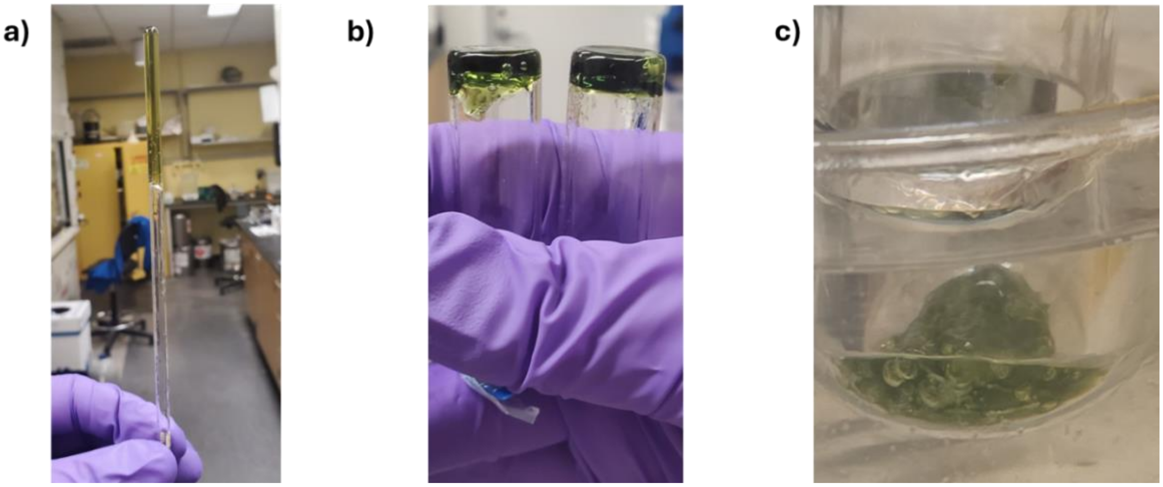

Figure S20. Gelled poly(**16**) products formed in $CDCl_3$ (0.5M, M:I = 100, 100 mins) after initiation at a) 35 °C b) 50 °C (left), 80 °C (right) c) 100 °C.

Monomer	Polymer	Entry	Solvent	Temp. (°C)	Reaction Time (mins)	Conversion (%)[a]	$M_{n(SEC)}$[b] (kDa)	Đ[b]
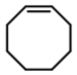 17	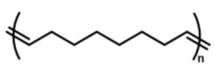 poly(17)	1	$CDCl_3$	60	45	34	114.3	1.60
		2	tol-d_8	60	45	94	102.8	2.22

Table S1. Ring-Opening Metathesis Polymerization (ROMP) of 17 in $CDCl_3$ and tol-d_8 using complex 11 at 60 °C. aConversion was monitored via 1H NMR. bexperimentally determined molecular weights ($M_{n(SEC)}$) and dispersities (Đ) are based on size exclusion chromatography (SEC) in $CHCl_3$ and calibrated with polystyrene standards. ^{1}H NMR spectra and SEC traces can be found on pages S58-S60.

5. Crystallographic Data of Complex 11

Experimental. Single light green plate crystals of **TZ06-092** recrystallized by vapor diffusion from hexane into a toluene solution of **11**. A suitable crystal with dimensions 0.16 × 0.13 × 0.07 mm^3 was selected and mounted on a Rigaku XtaLAB Synergy-S diffractometer. The crystal was kept at a steady T = 100(1) K during data collection. The structure was solved with the ShelXT 2018/2[5] solution program using dual methods and by using Olex2[6] as the graphical interface. The model was refined with olex2.refine 1.3-dev[7] using full matrix least squares minimisation on $\boldsymbol{F^2}$.

Structure Quality Indicators

**Reflections:**	d min (CuKα) 2Θ=136.4° 0.83	I/σ(I) 21.0	Rint m=3.79 6.38%	Full 135.4° 96% to 136.4° 96.6
**Refinement:**	Shift 0.002	Max Peak 2.0	Min Peak -0.8	GooF 1.049

A light green plate-shaped crystal with dimensions 0.16 × 0.13 × 0.07 mm^3 was mounted. Data were collected using a Rigaku XtaLAB Synergy, Dualflex, HyPix diffractometer operating at T = 100(1) K.

Data were measured using w scans with Cu K_a radiation. The diffraction pattern was indexed and the total number of runs and images was based on the strategy calculation from the program CrysAlis^{Pro} system (CCD 40.84a 64-bit (release 31-07-2020)). The maximum resolution achieved was Q = 68.187° (0.83 Å).

The unit cell was refined using CrysAlis^{Pro} on 8594 reflections, 28% of the observed reflections.

Data reduction, scaling and absorption corrections were performed using CrysAlisPro (Rigaku, V1.171.40.84a, 2020). The final completeness is 98.41 % out to 65.07° in *Q*. A numerical absorption correction based on gaussian integration over a multifaceted crystal model was performed using CrysAlis^{Pro} 1.171.44.57a (Rigaku Oxford Diffraction, 2024). An empirical absorption correction using spherical harmonics, implemented in SCALE3 ABSPACK scaling algorithm was also applied The absorption coefficient *m* of this material is 4.511 mm^{-1} at this wavelength (*l* = 1.54184Å) and the minimum and maximum transmissions are 0.609 and 0.975.

The structure was solved and the space group $P2_1/n$ (# 14) determined by the ShelXT 2018/2[5] structure solution program using using dual methods and refined by full matrix least squares minimisation on $\boldsymbol{F}^2$ using version of olex2.refine 1.3-dev.[7] All non-hydrogen atoms were refined anisotropically. Hydrogen atom positions were calculated geometrically and refined using the riding model.

_refine_special_details: Refined as a 2-component twin.

There is a single formula unit in the asymmetric unit, (Z′ = 1, Z = 4) (one Ru-complex and two toluene molecules), consistent with the empirical formula $C_{52}H_{58}Cl_2N_2RuS$.

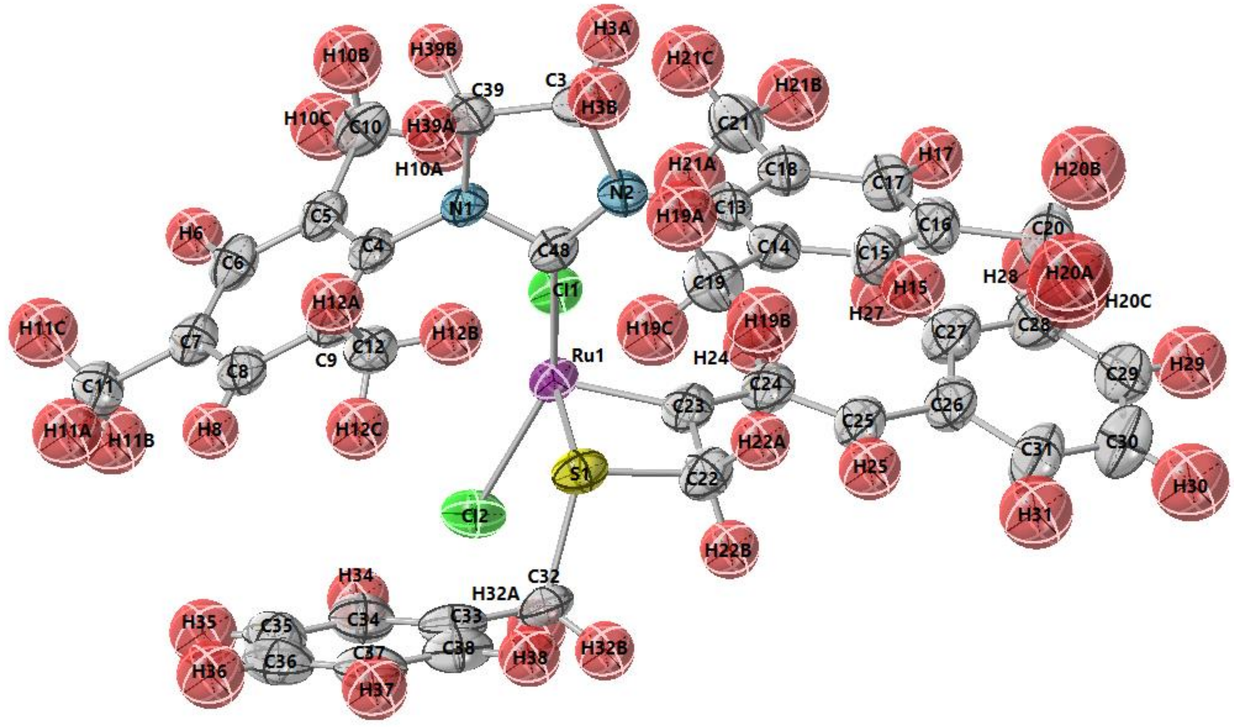

Figure S21. Ellipsoid model of complex **11** with 50% probability.

Table S2. Crystal data of complex **11**.

Formula	$C_{52}H_{58}Cl_2N_2RuS$
$D_{calc.}$/ g cm^{-3}	1.318
m/mm^{-1}	4.511
Formula Weight	915.03
Color	light green
Shape	plate
Size/mm^3	0.16×0.13×0.07
T/K	100(1)
Crystal System	monoclinic
Space Group	$P2_1/n$
a/Å	8.26600(10)
b/Å	39.1660(9)
c/Å	14.2459(3)
a/°	90
b/°	90.895(2)
g/°	90
V/Å^3	4611.49(15)
Z	4
Z'	1
Wavelength/Å	1.54184
Radiation type	Cu K_a
Q_{min}/°	2.26
Q_{max}/°	65.07
Index range h	-9 ≥ h ≥ 9
Index range k	-46 ≥ k ≥ 35
Index range l	-17 ≥ l ≥ 16
Measured Refl's.	30534
Indep't Refl's	8050
Refl's I≥2*s*(I)	6638
R_{int}	0.0638
Parameters	598
Restraints	1049
Largest Peak/eÅ^3	2.025
Deepest Hole/eÅ^3	-0.818
GooF	1.049
R_1 (I≥2*s*(I) / all)	0.0698 / 0.0812
wR_2 (I≥2*s*(I) / all)	0.1857 / 0.1937

Table S3: Fractional Atomic Coordinates (×10^4) and Equivalent Isotropic Displacement Parameters (Å^2×10^3) for **11**. U_{eq} is defined as 1/3 of the trace of the orthogonalised U_{ij}.

**Atom**	**x**	**y**	**z**	U_{eq}
Ru1	5672.7(5)	3377.4(2)	4825.4(3)	40.2(2)
Cl1	6842.0(15)	3694.5(3)	3569.0(10)	46.7(3)
Cl2	7894.3(17)	2994.2(4)	4751.0(11)	56.8(4)
S1	4670.0(16)	3138.9(4)	6219.9(10)	48.8(4)
N1	2685(5)	3367.7(11)	3742(3)	43.1(10)
N2	2428(5)	3787.8(12)	4719(4)	46.2(11)
C3	893(8)	3795.9(18)	4177(5)	63.2(18)
C4	3338(6)	3058.2(14)	3345(4)	43.8(12)
C5	4051(7)	3072.6(16)	2455(4)	48.8(13)
C6	4665(7)	2769.2(17)	2100(4)	52.7(14)
C7	4632(6)	2465.8(16)	2583(4)	50.3(14)
C8	3887(7)	2457.4(16)	3448(4)	50.6(14)
C9	3186(6)	2753.5(15)	3833(4)	44.0(12)
C10	4064(9)	3396.2(17)	1878(5)	59.7(16)
C11	5351(8)	2142.9(18)	2180(5)	61.7(17)
C12	2163(7)	2718.1(16)	4700(4)	49.7(13)
C13	2727(7)	4052.9(14)	5396(5)	48.9(14)
C14	2119(7)	4023.7(16)	6282(5)	53.2(14)
C15	2386(9)	4290.5(19)	6911(5)	64.3(17)
C16	3237(9)	4576.5(19)	6676(6)	68.0(19)
C17	3793(8)	4604.2(17)	5773(6)	61.4(17)
C18	3550(7)	4347.8(16)	5101(5)	53.3(14)
C19	1167(9)	3707.7(18)	6580(6)	65.3(17)
C20	3586(12)	4861(2)	7389(7)	91(3)
C21	4162(9)	4388.0(19)	4127(6)	66.4(17)
C22	5565(8)	3527.4(17)	6699(5)	55.4(14)
C23	6073(7)	3684.3(16)	5781(5)	51.4(13)
C24	6917(7)	4004.8(17)	5782(5)	57.1(14)
C25	7196(8)	4178.1(13)	6578(5)	62.7(17)
C26	7986(8)	4519.8(14)	6677(4)	64.5(18)
C27	8324(9)	4723.2(16)	5904(4)	74(2)
C28	9038(10)	5042.0(17)	6029(5)	85(2)
C29	9439(11)	5154(2)	6931(5)	87(3)
C30	9079(12)	4954.4(19)	7708(6)	98(3)
C31	8370(11)	4635.4(19)	7578(4)	87(3)

Atom	x	y	z	U_{eq}
C32	5954(7)	2802.3(17)	6781(4)	55.5(15)
C33	5281(7)	2466.5(18)	6630(3)	59.0(16)
C34	5681(8)	2274.1(15)	5851(5)	61.4(17)
C35	5020(8)	1950.6(15)	5725(5)	73(2)
C36	3946(8)	1820.4(18)	6377(4)	74(2)
C37	3563(8)	2013.7(14)	7163(5)	67.3(19)
C38	4235(7)	2336.1(15)	7291(4)	60.0(17)
C39	1021(6)	3479.6(15)	3545(5)	48.0(13)
C48	3432(6)	3541.3(14)	4440(4)	40.4(11)
C1S_1	1103(14)	649(4)	6389(12)	111(2)
C2S_1	2848(12)	657(2)	6097(7)	91(3)
C3S_1	3366(14)	457(4)	5360(10)	103(3)
C4S_1	4944(15)	478(4)	5053(10)	114(4)
C5S_1	6043(14)	683(4)	5530(11)	125(5)
C6S_1	5530(14)	885(4)	6265(11)	111(2)
C7S_1	3954(14)	865(3)	6566(9)	102(3)
C1S_2	6010(20)	807(6)	6160(20)	111(2)
C2S_2	4301(17)	684(4)	6005(12)	118(6)
C3S_2	3060(20)	817(5)	6532(14)	102(3)
C4S_2	1490(20)	699(7)	6415(18)	111(2)
C5S_2	1152(19)	453(5)	5745(16)	111(2)
C6S_2	2390(20)	319(5)	5216(15)	110(5)
C7S_2	3940(20)	445(6)	5312(16)	103(3)
C1S_3	7670(16)	3732(5)	9240(30)	119(5)
C2S_3	6004(13)	3574(3)	9210(16)	103(3)
C3S_3	5813(14)	3227(3)	9330(20)	97(3)
C4S_3	4297(16)	3078(3)	9266(13)	103(3)
C5S_3	2966(14)	3279(4)	9051(12)	102(4)
C6S_3	3129(15)	3631(4)	9020(20)	113(4)
C7S_3	4644(15)	3778(3)	9081(15)	103(4)
C1S_4	8253(16)	3656(6)	9140(40)	119(5)
C2S_4	6435(14)	3622(4)	9150(20)	103(3)
C3S_4	5724(17)	3304(4)	9220(30)	97(3)
C4S_4	4060(18)	3268(4)	9169(17)	104(5)
C5S_4	3092(16)	3555(5)	9120(30)	113(4)
C6S_4	3796(17)	3876(4)	9087(16)	103(3)
C7S_4	5458(18)	3909(4)	9057(18)	100(5)

Table S4: Anisotropic Displacement Parameters (×10^4) for **11**. The anisotropic displacement factor exponent takes the form: $-2p^2[h^2a^{*2} \times U_{11} + ... +2hka^* \times b^* \times U_{12}]$

**Atom**	U_{11}	U_{22}	U_{33}	U_{23}	U_{13}	U_{12}
Ru1	26.7(3)	54.6(3)	39.3(3)	-2.54(17)	-1.26(15)	2.95(15)
Cl1	34.4(6)	56.9(8)	48.8(7)	2.4(6)	3.9(5)	-1.1(5)
Cl2	36.9(7)	74.4(10)	59.3(9)	11.4(7)	8.8(6)	14.8(6)
S1	36.9(7)	67.0(9)	42.6(7)	3.4(6)	0.4(5)	6.7(6)
N1	28(2)	54(3)	46(3)	-6(2)	-7.9(19)	4.7(17)
N2	28(2)	50(3)	60(3)	-6(2)	-5(2)	5.8(18)
C3	39(3)	72(4)	78(5)	-11(3)	-21(3)	13(3)
C4	28(2)	57(3)	46(3)	-10(2)	-8(2)	4(2)
C5	36(3)	64(4)	46(3)	-8(3)	-6(2)	-2(2)
C6	40(3)	70(4)	48(3)	-12(3)	1(2)	-6(3)
C7	30(3)	68(4)	53(3)	-16(3)	-2(2)	4(2)
C8	41(3)	56(3)	54(4)	-6(3)	-6(3)	1(2)
C9	28(2)	58(3)	46(3)	-9(2)	-4(2)	5(2)
C10	60(4)	71(4)	47(4)	1(3)	0(3)	-6(3)
C11	45(3)	71(4)	69(4)	-19(3)	-2(3)	11(3)
C12	36(3)	64(4)	49(3)	-2(3)	1(2)	7(2)
C13	36(3)	45(3)	65(4)	-8(3)	-6(3)	7(2)
C14	43(3)	58(3)	59(4)	-2(3)	3(3)	7(3)
C15	57(4)	76(4)	60(4)	-15(3)	2(3)	9(3)
C16	59(4)	68(4)	76(5)	-22(4)	-8(4)	14(3)
C17	42(3)	56(4)	86(5)	-14(3)	-3(3)	-1(3)
C18	40(3)	55(3)	65(4)	-5(3)	-4(3)	6(2)
C19	52(4)	69(4)	75(5)	-2(3)	12(3)	2(3)
C20	87(6)	90(6)	98(7)	-41(5)	-9(5)	5(5)
C21	64(4)	63(4)	72(5)	-1(3)	8(3)	1(3)
C22	46(3)	69(4)	51(3)	-5(3)	0(3)	0(3)
C23	30(2)	67(3)	57(3)	-9(2)	-6(2)	6.3(18)
C24	39(3)	70(3)	62(4)	-6(2)	-10(3)	1(2)
C25	57(4)	69(4)	61(4)	-12(3)	-13(3)	6(3)
C26	51(4)	68(4)	74(5)	-16(3)	-14(3)	3(3)
C27	69(5)	82(5)	69(5)	-5(4)	-14(4)	-7(4)
C28	75(5)	82(5)	97(7)	-12(5)	-18(5)	-2(4)
C29	80(6)	73(5)	106(7)	-17(5)	-25(5)	6(4)
C30	109(8)	96(7)	86(7)	-25(5)	-26(6)	-9(5)
C31	84(6)	104(6)	72(5)	-30(5)	-16(4)	-8(5)
C32	44(3)	82(4)	40(3)	-3(3)	-11(2)	20(3)
C33	40(3)	79(4)	58(4)	20(3)	-7(3)	12(3)
C34	46(3)	74(4)	64(4)	11(3)	5(3)	11(3)
C35	64(4)	79(5)	75(5)	1(4)	-8(4)	20(4)
C36	49(4)	74(5)	98(6)	24(4)	-15(4)	3(3)
C37	45(3)	78(5)	78(5)	28(4)	-6(3)	4(3)
C38	46(3)	83(5)	50(4)	20(3)	0(3)	11(3)
C39	28(3)	51(3)	65(4)	0(3)	-11(2)	3(2)
C48	26(2)	51(3)	45(3)	1(2)	0(2)	-5(2)

Atom	U_{11}	U_{22}	U_{33}	U_{23}	U_{13}	U_{12}
C1S_1	145(6)	88(5)	102(5)	13(4)	8(4)	18(4)
C2S_1	142(7)	71(7)	59(6)	14(4)	-9(5)	25(5)
C3S_1	146(8)	87(6)	76(5)	7(4)	-1(6)	36(7)
C4S_1	151(9)	93(10)	97(10)	7(7)	12(7)	29(8)
C5S_1	149(11)	107(11)	119(11)	-3(8)	17(9)	23(8)
C6S_1	145(6)	88(5)	102(5)	13(4)	8(4)	18(4)
C7S_1	145(6)	84(6)	76(6)	2(4)	1(5)	17(5)
C1S_2	145(6)	88(5)	102(5)	13(4)	8(4)	18(4)
C2S_2	141(7)	114(9)	101(9)	-12(7)	8(6)	15(6)
C3S_2	145(6)	84(6)	76(6)	2(4)	1(5)	17(5)
C4S_2	145(6)	88(5)	102(5)	13(4)	8(4)	18(4)
C5S_2	145(6)	88(5)	102(5)	13(4)	8(4)	18(4)
C6S_2	147(9)	96(14)	85(13)	15(9)	-5(8)	30(7)
C7S_2	146(8)	87(6)	76(5)	7(4)	-1(6)	36(7)
C1S_3	115(6)	132(11)	107(11)	-24(10)	-27(13)	22(7)
C2S_3	112(6)	127(6)	72(6)	-15(6)	-15(7)	29(4)
C3S_3	107(5)	125(6)	60(8)	-9(7)	-1(5)	35(5)
C4S_3	108(5)	142(8)	60(7)	-1(8)	5(6)	30(5)
C5S_3	100(8)	144(8)	62(9)	4(9)	5(8)	23(7)
C6S_3	104(5)	142(7)	93(9)	21(8)	5(6)	33(5)
C7S_3	111(6)	127(9)	71(9)	-2(9)	1(9)	32(5)
C1S_4	115(6)	132(11)	107(11)	-24(10)	-27(13)	22(7)
C2S_4	112(6)	127(6)	72(6)	-15(6)	-15(7)	29(4)
C3S_4	107(5)	125(6)	60(8)	-9(7)	-1(5)	35(5)
C4S_4	107(6)	142(10)	62(11)	14(11)	5(9)	33(7)
C5S_4	104(5)	142(7)	93(9)	21(8)	5(6)	33(5)
C6S_4	108(5)	142(8)	60(7)	-1(8)	5(6)	30(5)
C7S_4	111(6)	119(9)	70(12)	-21(10)	0(10)	24(6)

Table S5: Bond Lengths in Å for **11**.

Atom	Atom	Length/Å
Ru1	Cl1	2.3946(14)
Ru1	Cl2	2.3752(14)
Ru1	S1	2.3574(15)
Ru1	C23	1.842(6)
Ru1	C48	2.028(5)
S1	C22	1.820(7)
S1	C32	1.865(6)
N1	C4	1.446(7)
N1	C39	1.467(6)
N1	C48	1.346(7)
N2	C3	1.475(7)
N2	C13	1.436(7)
N2	C48	1.338(7)
C3	H3A	1.0980

Atom	Atom	Length/Å
C3	H3B	1.0980
C3	C39	1.536(9)
C4	C5	1.408(8)
C4	C9	1.388(8)
C5	C6	1.391(9)
C5	C10	1.510(9)
C6	H6	1.0800
C6	C7	1.374(9)
C7	C8	1.386(9)
C7	C11	1.514(8)
C8	H8	1.0800
C8	C9	1.412(8)
C9	C12	1.514(8)
C10	H10A	1.0981
C10	H10B	1.0981
C10	H10C	1.0981
C11	H11A	1.0981
C11	H11B	1.0981
C11	H11C	1.0981
C12	H12A	1.0981
C12	H12B	1.0981
C12	H12C	1.0981
C13	C14	1.370(9)
C13	C18	1.408(9)
C14	C15	1.392(9)
C14	C19	1.531(9)
C15	H15	1.0800
C15	C16	1.367(11)
C16	C17	1.377(11)
C16	C20	1.531(10)
C17	H17	1.0800
C17	C18	1.400(9)
C18	C21	1.492(10)
C19	H19A	1.0981
C19	H19B	1.0981
C19	H19C	1.0981
C20	H20A	1.0801
C20	H20B	1.0801
C20	H20C	1.0801
C21	H21A	1.0981
C21	H21B	1.0981
C21	H21C	1.0981
C22	H22A	1.0980
C22	H22B	1.0980
C22	C23	1.510(9)
C23	C24	1.436(9)

Atom	Atom	Length/Å
C24	H24	1.0780
C24	C25	1.338(9)
C25	H25	1.0780
C25	C26	1.495(2)
C26	C27	1.392(2)
C26	C31	1.393(2)
C27	H27	1.0800
C27	C28	1.3913(17)
C28	H28	1.0800
C28	C29	1.393(2)
C29	H29	1.0800
C29	C30	1.392(2)
C30	H30	1.0800
C30	C31	1.3913(17)
C31	H31	1.0800
C32	H32A	1.0980
C32	H32B	1.0980
C32	C33	1.443(10)
C33	C34	1.385(6)
C33	C38	1.386(6)
C34	H34	1.0800
C34	C35	1.390(2)
C35	H35	1.0800
C35	C36	1.392(2)
C36	H36	1.0800
C36	C37	1.392(2)
C37	H37	1.0800
C37	C38	1.390(2)
C38	H38	1.0800
C39	H39A	1.0980
C39	H39B	1.0980
C1S_1	H1SA_1	1.0981
C1S_1	H1SB_1	1.0981
C1S_1	H1SC_1	1.0981
C1S_1	C2S_1	1.508(8)
C2S_1	C3S_1	1.382(5)
C2S_1	C7S_1	1.388(6)
C3S_1	H3S_1	1.0780
C3S_1	C4S_1	1.384(6)
C4S_1	H4S_1	1.0780
C4S_1	C5S_1	1.383(6)
C5S_1	H5S_1	1.0780
C5S_1	C6S_1	1.385(6)
C6S_1	H6S_1	1.0780
C6S_1	C7S_1	1.380(6)
C7S_1	H7S_1	1.0780

Atom	Atom	Length/Å
C1S_2	H1SA_2	1.0981
C1S_2	H1SB_2	1.0981
C1S_2	H1SC_2	1.0981
C1S_2	C2S_2	1.508(8)
C2S_2	C3S_2	1.382(6)
C2S_2	C7S_2	1.388(6)
C3S_2	H3S_2	1.0780
C3S_2	C4S_2	1.384(6)
C4S_2	H4S_2	1.0780
C4S_2	C5S_2	1.383(6)
C5S_2	H5S_2	1.0780
C5S_2	C6S_2	1.384(6)
C6S_2	H6S_2	1.0780
C6S_2	C7S_2	1.381(6)
C7S_2	H7S_2	1.0780
C1S_3	H1SA_3	1.0981
C1S_3	H1SB_3	1.0981
C1S_3	H1SC_3	1.0981
C1S_3	C2S_3	1.508(8)
C2S_3	C3S_3	1.382(5)
C2S_3	C7S_3	1.388(6)
C3S_3	H3S_3	1.0780
C3S_3	C4S_3	1.384(6)
C4S_3	H4S_3	1.0780
C4S_3	C5S_3	1.383(6)
C5S_3	H5S_3	1.0780
C5S_3	C6S_3	1.385(6)
C6S_3	H6S_3	1.0780
C6S_3	C7S_3	1.381(6)
C7S_3	H7S_3	1.0780
C1S_4	H1SA_4	1.0981
C1S_4	H1SB_4	1.0981
C1S_4	H1SC_4	1.0981
C1S_4	C2S_4	1.508(8)
C2S_4	C3S_4	1.382(6)
C2S_4	C7S_4	1.388(6)
C3S_4	H3S_4	1.0780
C3S_4	C4S_4	1.384(6)
C4S_4	H4S_4	1.0780
C4S_4	C5S_4	1.383(6)
C5S_4	H5S_4	1.0780
C5S_4	C6S_4	1.384(6)
C6S_4	H6S_4	1.0780
C6S_4	C7S_4	1.381(6)
C7S_4	H7S_4	1.0780

Table S6: Bond Angles in ° for **11**.

Atom	Atom	Atom	Angle/°
Cl2	Ru1	Cl1	88.45(5)
S1	Ru1	Cl1	170.64(5)
S1	Ru1	Cl2	93.99(5)
C23	Ru1	Cl1	98.3(2)
C23	Ru1	Cl2	108.37(18)
C23	Ru1	S1	72.4(2)
C23	Ru1	C48	98.4(2)
C48	Ru1	Cl1	90.62(15)
C48	Ru1	Cl2	153.05(16)
C48	Ru1	S1	91.27(15)
C22	S1	Ru1	80.7(2)
C22	S1	C32	101.8(3)
C32	S1	Ru1	115.8(2)
C4	N1	C39	122.0(4)
C48	N1	C4	122.9(4)
C48	N1	C39	114.1(5)
C13	N2	C3	118.3(4)
C48	N2	C3	113.1(5)
C48	N2	C13	128.4(4)
N2	C3	H3A	111.2
N2	C3	H3B	111.2
N2	C3	C39	102.9(5)
H3A	C3	H3B	109.1
C39	C3	H3A	111.2
C39	C3	H3B	111.2
C5	C4	N1	118.9(5)
C9	C4	N1	119.2(5)
C9	C4	C5	121.9(5)
C4	C5	C10	122.0(5)
C6	C5	C4	117.0(6)
C6	C5	C10	120.9(6)
C5	C6	H6	118.4
C7	C6	C5	123.1(6)
C7	C6	H6	118.4
C6	C7	C8	118.6(6)
C6	C7	C11	121.4(6)
C8	C7	C11	120.0(6)
C7	C8	H8	119.4
C7	C8	C9	121.1(6)
C9	C8	H8	119.4
C4	C9	C8	118.1(5)
C4	C9	C12	122.9(5)
C8	C9	C12	118.7(5)
C5	C10	H10A	109.5
C5	C10	H10B	109.5

Atom	Atom	Atom	Angle/°
C5	C10	H10C	109.5
H10A	C10	H10B	109.5
H10A	C10	H10C	109.5
H10B	C10	H10C	109.5
C7	C11	H11A	109.5
C7	C11	H11B	109.5
C7	C11	H11C	109.5
H11A	C11	H11B	109.5
H11A	C11	H11C	109.5
H11B	C11	H11C	109.5
C9	C12	H12A	109.5
C9	C12	H12B	109.5
C9	C12	H12C	109.5
H12A	C12	H12B	109.5
H12A	C12	H12C	109.5
H12B	C12	H12C	109.5
C14	C13	N2	119.8(5)
C14	C13	C18	122.0(6)
C18	C13	N2	118.1(6)
C13	C14	C15	118.3(6)
C13	C14	C19	121.4(6)
C15	C14	C19	120.4(6)
C14	C15	H15	118.8
C16	C15	C14	122.3(7)
C16	C15	H15	118.8
C15	C16	C17	118.3(6)
C15	C16	C20	121.7(8)
C17	C16	C20	120.0(8)
C16	C17	H17	118.8
C16	C17	C18	122.4(7)
C18	C17	H17	118.8
C13	C18	C21	122.5(6)
C17	C18	C13	116.6(6)
C17	C18	C21	120.9(6)
C14	C19	H19A	109.5
C14	C19	H19B	109.5
C14	C19	H19C	109.5
H19A	C19	H19B	109.5
H19A	C19	H19C	109.5
H19B	C19	H19C	109.5
C16	C20	H20A	109.5
C16	C20	H20B	109.5
C16	C20	H20C	109.5
H20A	C20	H20B	109.5
H20A	C20	H20C	109.5
H20B	C20	H20C	109.5
C18	C21	H21A	109.5

Atom	Atom	Atom	Angle/°
C18	C21	H21B	109.5
C18	C21	H21C	109.5
H21A	C21	H21B	109.5
H21A	C21	H21C	109.5
H21B	C21	H21C	109.5
S1	C22	H22A	112.2
S1	C22	H22B	112.2
H22A	C22	H22B	109.8
C23	C22	S1	97.6(4)
C23	C22	H22A	112.2
C23	C22	H22B	112.2
C22	C23	Ru1	109.0(4)
C24	C23	Ru1	130.7(5)
C24	C23	C22	119.8(6)
C23	C24	H24	119.3
C25	C24	C23	121.4(7)
C25	C24	H24	119.3
C24	C25	H25	116.4
C24	C25	C26	127.1(6)
C26	C25	H25	116.4
C27	C26	C25	122.0(5)
C27	C26	C31	119.9(6)
C31	C26	C25	118.1(5)
C26	C27	H27	119.9
C28	C27	C26	120.1(6)
C28	C27	H27	119.9
C27	C28	H28	120.1
C27	C28	C29	119.8(7)
C29	C28	H28	120.1
C28	C29	H29	119.8
C30	C29	C28	120.3(8)
C30	C29	H29	119.8
C29	C30	H30	120.2
C31	C30	C29	119.6(8)
C31	C30	H30	120.2
C26	C31	H31	119.9
C30	C31	C26	120.3(7)
C30	C31	H31	119.9
S1	C32	H32A	109.3
S1	C32	H32B	109.3
H32A	C32	H32B	108.0
C33	C32	S1	111.4(4)
C33	C32	H32A	109.3
C33	C32	H32B	109.3
C34	C33	C32	121.3(5)
C34	C33	C38	120.2(6)
C38	C33	C32	118.5(5)

Atom	Atom	Atom	Angle/°
C33	C34	H34	119.9
C33	C34	C35	120.1(6)
C35	C34	H34	119.9
C34	C35	H35	120.0
C34	C35	C36	120.0(7)
C36	C35	H35	120.0
C35	C36	H36	120.2
C37	C36	C35	119.5(7)
C37	C36	H36	120.2
C36	C37	H37	119.9
C38	C37	C36	120.3(6)
C38	C37	H37	119.9
C33	C38	C37	119.9(6)
C33	C38	H38	120.1
C37	C38	H38	120.1
N1	C39	C3	101.7(4)
N1	C39	H39A	111.4
N1	C39	H39B	111.4
C3	C39	H39A	111.4
C3	C39	H39B	111.4
H39A	C39	H39B	109.3
N1	C48	Ru1	116.5(4)
N2	C48	Ru1	135.7(4)
N2	C48	N1	107.7(4)
H1SA_1	C1S_1	H1SB_1	109.5
H1SA_1	C1S_1	H1SC_1	109.5
H1SB_1	C1S_1	H1SC_1	109.5
C2S_1	C1S_1	H1SA_1	109.5
C2S_1	C1S_1	H1SB_1	109.5
C2S_1	C1S_1	H1SC_1	109.5
C3S_1	C2S_1	C1S_1	120.4(6)
C3S_1	C2S_1	C7S_1	119.3(6)
C7S_1	C2S_1	C1S_1	120.3(6)
C2S_1	C3S_1	H3S_1	119.6
C2S_1	C3S_1	C4S_1	120.8(6)
C4S_1	C3S_1	H3S_1	119.6
C3S_1	C4S_1	H4S_1	120.2
C5S_1	C4S_1	C3S_1	119.6(6)
C5S_1	C4S_1	H4S_1	120.2
C4S_1	C5S_1	H5S_1	120.1
C4S_1	C5S_1	C6S_1	119.8(6)
C6S_1	C5S_1	H5S_1	120.1
C5S_1	C6S_1	H6S_1	119.9
C7S_1	C6S_1	C5S_1	120.3(6)
C7S_1	C6S_1	H6S_1	119.9
C2S_1	C7S_1	H7S_1	120.0
C6S_1	C7S_1	C2S_1	120.1(6)

Atom	Atom	Atom	Angle/°
C6S_1	C7S_1	H7S_1	120.0
H1SA_2	C1S_2	H1SB_2	109.5
H1SA_2	C1S_2	H1SC_2	109.5
H1SB_2	C1S_2	H1SC_2	109.5
C2S_2	C1S_2	H1SA_2	109.5
C2S_2	C1S_2	H1SB_2	109.5
C2S_2	C1S_2	H1SC_2	109.5
C3S_2	C2S_2	C1S_2	120.4(6)
C3S_2	C2S_2	C7S_2	119.2(6)
C7S_2	C2S_2	C1S_2	120.4(6)
C2S_2	C3S_2	H3S_2	119.6
C2S_2	C3S_2	C4S_2	120.8(6)
C4S_2	C3S_2	H3S_2	119.6
C3S_2	C4S_2	H4S_2	120.2
C5S_2	C4S_2	C3S_2	119.6(6)
C5S_2	C4S_2	H4S_2	120.2
C4S_2	C5S_2	H5S_2	120.1
C4S_2	C5S_2	C6S_2	119.8(7)
C6S_2	C5S_2	H5S_2	120.1
C5S_2	C6S_2	H6S_2	119.9
C7S_2	C6S_2	C5S_2	120.2(7)
C7S_2	C6S_2	H6S_2	119.9
C2S_2	C7S_2	H7S_2	120.0
C6S_2	C7S_2	C2S_2	120.0(6)
C6S_2	C7S_2	H7S_2	120.0
H1SA_3	C1S_3	H1SB_3	109.5
H1SA_3	C1S_3	H1SC_3	109.5
H1SB_3	C1S_3	H1SC_3	109.5
C2S_3	C1S_3	H1SA_3	109.5
C2S_3	C1S_3	H1SB_3	109.5
C2S_3	C1S_3	H1SC_3	109.5
C3S_3	C2S_3	C1S_3	120.4(6)
C3S_3	C2S_3	C7S_3	119.2(6)
C7S_3	C2S_3	C1S_3	120.4(6)
C2S_3	C3S_3	H3S_3	119.6
C2S_3	C3S_3	C4S_3	120.8(6)
C4S_3	C3S_3	H3S_3	119.6
C3S_3	C4S_3	H4S_3	120.3
C5S_3	C4S_3	C3S_3	119.4(6)
C5S_3	C4S_3	H4S_3	120.3
C4S_3	C5S_3	H5S_3	120.2
C4S_3	C5S_3	C6S_3	119.7(7)
C6S_3	C5S_3	H5S_3	120.2
C5S_3	C6S_3	H6S_3	119.9
C7S_3	C6S_3	C5S_3	120.2(6)
C7S_3	C6S_3	H6S_3	119.9
C2S_3	C7S_3	H7S_3	120.0

Atom	Atom	Atom	Angle/°
C6S_3	C7S_3	C2S_3	120.0(6)
C6S_3	C7S_3	H7S_3	120.0
H1SA_4	C1S_4	H1SB_4	109.5
H1SA_4	C1S_4	H1SC_4	109.5
H1SB_4	C1S_4	H1SC_4	109.5
C2S_4	C1S_4	H1SA_4	109.5
C2S_4	C1S_4	H1SB_4	109.5
C2S_4	C1S_4	H1SC_4	109.5
C3S_4	C2S_4	C1S_4	120.4(6)
C3S_4	C2S_4	C7S_4	119.2(6)
C7S_4	C2S_4	C1S_4	120.4(6)
C2S_4	C3S_4	H3S_4	119.6
C2S_4	C3S_4	C4S_4	120.8(6)
C4S_4	C3S_4	H3S_4	119.6
C3S_4	C4S_4	H4S_4	120.2
C5S_4	C4S_4	C3S_4	119.5(6)
C5S_4	C4S_4	H4S_4	120.2
C4S_4	C5S_4	H5S_4	120.1
C4S_4	C5S_4	C6S_4	119.8(7)
C6S_4	C5S_4	H5S_4	120.1
C5S_4	C6S_4	H6S_4	119.9
C7S_4	C6S_4	C5S_4	120.3(6)
C7S_4	C6S_4	H6S_4	119.9
C2S_4	C7S_4	H7S_4	120.0
C6S_4	C7S_4	C2S_4	120.0(6)
C6S_4	C7S_4	H7S_4	120.0

Table S7: Torsion Angles in ° for **11**.

Atom	Atom	Atom	Atom	Angle/°
Ru1	S1	C22	C23	4.2(3)
Ru1	S1	C32	C33	-105.0(4)
Ru1	C23	C24	C25	-174.3(5)
Cl1	Ru1	C23	C22	-175.0(4)
Cl1	Ru1	C23	C24	-3.4(6)
Cl2	Ru1	C23	C22	-83.9(4)
Cl2	Ru1	C23	C24	87.7(6)
S1	Ru1	C23	C22	4.5(3)
S1	Ru1	C23	C24	176.0(6)
S1	C22	C23	Ru1	-5.6(4)
S1	C22	C23	C24	-178.2(5)
S1	C32	C33	C34	89.5(6)
S1	C32	C33	C38	-91.4(6)
N1	C4	C5	C6	180.0(5)
N1	C4	C5	C10	-3.8(8)
N1	C4	C9	C8	-177.6(5)

Atom	Atom	Atom	Atom	Angle/°
N1	C4	C9	C12	8.8(8)
N2	C3	C39	N1	-6.8(7)
N2	C13	C14	C15	-177.9(6)
N2	C13	C14	C19	2.0(8)
N2	C13	C18	C17	178.8(5)
N2	C13	C18	C21	-2.0(8)
C3	N2	C13	C14	84.3(7)
C3	N2	C13	C18	-91.4(7)
C3	N2	C48	Ru1	179.8(5)
C3	N2	C48	N1	-1.4(7)
C4	N1	C39	C3	175.8(6)
C4	N1	C48	Ru1	6.5(7)
C4	N1	C48	N2	-172.6(5)
C4	C5	C6	C7	-1.4(9)
C5	C4	C9	C8	5.1(8)
C5	C4	C9	C12	-168.5(5)
C5	C6	C7	C8	3.0(9)
C5	C6	C7	C11	-178.5(5)
C6	C7	C8	C9	-0.5(8)
C7	C8	C9	C4	-3.4(8)
C7	C8	C9	C12	170.5(5)
C9	C4	C5	C6	-2.8(8)
C9	C4	C5	C10	173.5(5)
C10	C5	C6	C7	-177.8(6)
C11	C7	C8	C9	-179.0(5)
C13	N2	C3	C39	180.0(5)
C13	N2	C48	Ru1	6.0(10)
C13	N2	C48	N1	-175.2(6)
C13	C14	C15	C16	-0.5(10)
C14	C13	C18	C17	3.2(9)
C14	C13	C18	C21	-177.6(6)
C14	C15	C16	C17	2.4(11)
C14	C15	C16	C20	-177.2(7)
C15	C16	C17	C18	-1.5(10)
C16	C17	C18	C13	-1.2(9)
C16	C17	C18	C21	179.6(7)
C18	C13	C14	C15	-2.4(9)
C18	C13	C14	C19	177.5(6)
C19	C14	C15	C16	179.6(7)
C20	C16	C17	C18	178.1(7)
C22	S1	C32	C33	169.7(4)
C22	C23	C24	C25	-3.5(9)
C23	C24	C25	C26	-177.0(6)
C24	C25	C26	C27	9.7(11)
C24	C25	C26	C31	-171.1(8)
C25	C26	C27	C28	179.0(7)
C25	C26	C31	C30	-178.8(8)

Atom	Atom	Atom	Atom	Angle/°
C26	C27	C28	C29	1.1(13)
C27	C26	C31	C30	0.4(13)
C27	C28	C29	C30	-2.2(14)
C28	C29	C30	C31	2.4(15)
C29	C30	C31	C26	-1.5(15)
C31	C26	C27	C28	-0.2(12)
C32	S1	C22	C23	118.8(4)
C32	C33	C34	C35	179.8(6)
C32	C33	C38	C37	179.8(5)
C33	C34	C35	C36	0.4(10)
C34	C33	C38	C37	-1.2(9)
C34	C35	C36	C37	-1.1(10)
C35	C36	C37	C38	0.6(10)
C36	C37	C38	C33	0.5(9)
C38	C33	C34	C35	0.7(9)
C39	N1	C4	C5	87.2(7)
C39	N1	C4	C9	-90.2(7)
C39	N1	C48	Ru1	175.3(4)
C39	N1	C48	N2	-3.8(7)
C48	Ru1	C23	C22	93.1(4)
C48	Ru1	C23	C24	-95.3(6)
C48	N1	C4	C5	-104.9(6)
C48	N1	C4	C9	77.8(7)
C48	N1	C39	C3	6.9(7)
C48	N2	C3	C39	5.5(7)
C48	N2	C13	C14	-102.2(7)
C48	N2	C13	C18	82.2(8)
C1S_1	C2S_1	C3S_1	C4S_1	176.3(13)
C1S_1	C2S_1	C7S_1	C6S_1	-176.6(13)
C2S_1	C3S_1	C4S_1	C5S_1	4(2)
C3S_1	C2S_1	C7S_1	C6S_1	4(2)
C3S_1	C4S_1	C5S_1	C6S_1	-5(3)
C4S_1	C5S_1	C6S_1	C7S_1	5(3)
C5S_1	C6S_1	C7S_1	C2S_1	-4(3)
C7S_1	C2S_1	C3S_1	C4S_1	-4(2)
C1S_2	C2S_2	C3S_2	C4S_2	-178.4(19)
C1S_2	C2S_2	C7S_2	C6S_2	176.1(19)
C2S_2	C3S_2	C4S_2	C5S_2	-2(4)
C3S_2	C2S_2	C7S_2	C6S_2	-6(3)
C3S_2	C4S_2	C5S_2	C6S_2	2(4)
C4S_2	C5S_2	C6S_2	C7S_2	-4(4)
C5S_2	C6S_2	C7S_2	C2S_2	6(4)
C7S_2	C2S_2	C3S_2	C4S_2	4(3)
C1S_3	C2S_3	C3S_3	C4S_3	-177.3(16)
C1S_3	C2S_3	C7S_3	C6S_3	177.8(18)
C2S_3	C3S_3	C4S_3	C5S_3	2(3)
C3S_3	C2S_3	C7S_3	C6S_3	-4(3)

Atom	Atom	Atom	Atom	Angle/°
C3S_3	C4S_3	C5S_3	C6S_3	-8(3)
C4S_3	C5S_3	C6S_3	C7S_3	9(3)
C5S_3	C6S_3	C7S_3	C2S_3	-3(4)
C7S_3	C2S_3	C3S_3	C4S_3	4(2)
C1S_4	C2S_4	C3S_4	C4S_4	-175.9(19)
C1S_4	C2S_4	C7S_4	C6S_4	-178.5(17)
C2S_4	C3S_4	C4S_4	C5S_4	-5(4)
C3S_4	C2S_4	C7S_4	C6S_4	3(3)
C3S_4	C4S_4	C5S_4	C6S_4	2(5)
C4S_4	C5S_4	C6S_4	C7S_4	3(5)
C5S_4	C6S_4	C7S_4	C2S_4	-6(4)
C7S_4	C2S_4	C3S_4	C4S_4	3(3)

Table S8: Hydrogen Fractional Atomic Coordinates (×10^4) and Equivalent Isotropic Displacement Parameters (Å^2×10^3) for **11**. U_{eq} is defined as 1/3 of the trace of the orthogonalised U_{ij}.

Atom	x	y	z	U_{eq}
H3A	789.21	4030.37	3758.19	76
H3B	-151.42	3777.26	4641.4	76
H6	5192.07	2772.13	1410.81	63
H8	3843.12	2220.25	3833.2	61
H10A	4436.83	3611.55	2325.21	90
H10B	2845.51	3444.24	1589.35	90
H10C	4918.27	3368.1	1300.79	90
H11A	4896.05	1920.65	2564.13	93
H11B	6675.37	2152.95	2247.26	93
H11C	5003	2121.03	1435.03	93
H12A	928.72	2643.13	4492.54	75
H12B	2129.52	2963.34	5072.67	75
H12C	2690.68	2522.18	5163.4	75
H15	1903.67	4270.78	7608.73	77
H17	4441.28	4832.09	5576.38	74
H19A	-8.59	3703.74	6209.82	98
H19B	974.48	3715.81	7340.34	98
H19C	1851.08	3476.79	6402.8	98
H20A	3069.16	4793.45	8055.72	137
H20B	3056.05	5096.64	7140.17	137
H20C	4877.6	4892.31	7473.62	137
H21A	5117.22	4199.26	4002.68	100
H21B	4653.53	4646.27	4039.73	100
H21C	3163.64	4347.93	3620.64	100
H22A	4677.27	3685.47	7065.06	67
H22B	6602.1	3474.29	7167.73	67
H24	7335.13	4107.96	5127.47	68
H25	6807.24	4058.24	7216.79	75
H27	8031.13	4633.53	5204.7	88
H28	9280.24	5201.79	5430.03	102

Atom	x	y	z	U_{eq}
H29	10031.48	5397.83	7027.64	104
H30	9349.83	5046.3	8408.02	117
H31	8114.97	4476.81	8178.23	104
H32A	7174.41	2812.67	6488.29	67
H32B	6057.77	2851.84	7538.19	67
H34	6507.96	2375.47	5342.32	74
H35	5339.38	1800.37	5119.39	87
H36	3414.05	1571.03	6274.62	89
H37	2739.94	1912.92	7674.84	81
H38	3942.95	2484.54	7904.26	72
H39A	140.26	3283.1	3739.91	58
H39B	844.18	3545.87	2801.94	58
H1SA_1	502.88	886.61	6171.11	167
H1SB_1	1044.07	622.39	7154.85	167
H1SC_1	489.7	430.81	6052.77	167
H3S_1	2531.7	283.39	5020.68	123
H4S_1	5313.06	334.51	4446.84	136
H5S_1	7295.33	684.75	5330.52	150
H6S_1	6365.42	1058.76	6603.04	134
H7S_1	3581.32	1010.74	7167.06	122
H1SA_2	6307.29	996.68	5618.13	167
H1SB_2	6849.32	589.54	6105.35	167
H1SC_2	6134.81	923.11	6854.9	167
H3S_2	3317	1014.01	7040.13	122
H4S_2	537.07	799.55	6843.35	134
H5S_2	-73.56	365.64	5634.83	134
H6S_2	2140.71	114.45	4726.88	131
H7S_2	4877.24	358.34	4849.67	123
H1SA_3	7971.44	3809.81	9961.3	178
H1SB_3	7690.75	3956.27	8777.33	178
H1SC_3	8559.94	3544.12	9000.06	178
H3S_3	6857.08	3069.54	9477.22	117
H4S_3	4154.21	2807.62	9382.78	124
H5S_3	1807.19	3162.31	8906.13	122
H6S_3	2069.3	3789.73	8956.41	136
H7S_3	4771.38	4051.29	9026.12	124
H1SA_4	8747.9	3603.03	9842.62	178
H1SB_4	8578.09	3917.22	8930.84	178
H1SC_4	8754.77	3473.91	8635.04	178
H3S_4	6474.53	3081.39	9320.31	117
H4S_4	3520.89	3017.06	9169.05	124
H5S_4	1793.28	3530.3	9103.69	136
H6S_4	3044.16	4100.76	9085.31	124
H7S_4	5995.72	4156.89	8964.06	120

Table S9: Atomic Occupancies for all atoms that are not fully occupied in **11**.

Atom	Occupancy
C1S_1	0.632(10)
H1SA_1	0.632(10)
H1SB_1	0.632(10)
H1SC_1	0.632(10)
C2S_1	0.632(10)
C3S_1	0.632(10)
H3S_1	0.632(10)
C4S_1	0.632(10)
H4S_1	0.632(10)
C5S_1	0.632(10)
H5S_1	0.632(10)
C6S_1	0.632(10)
H6S_1	0.632(10)
C7S_1	0.632(10)
H7S_1	0.632(10)
C1S_2	0.368(10)
H1SA_2	0.368(10)
H1SB_2	0.368(10)
H1SC_2	0.368(10)
C2S_2	0.368(10)
C3S_2	0.368(10)
H3S_2	0.368(10)
C4S_2	0.368(10)
H4S_2	0.368(10)
C5S_2	0.368(10)
H5S_2	0.368(10)
C6S_2	0.368(10)
H6S_2	0.368(10)
C7S_2	0.368(10)
H7S_2	0.368(10)
C1S_3	0.566(12)
H1SA_3	0.566(12)
H1SB_3	0.566(12)
H1SC_3	0.566(12)
C2S_3	0.566(12)
C3S_3	0.566(12)
H3S_3	0.566(12)
C4S_3	0.566(12)
H4S_3	0.566(12)
C5S_3	0.566(12)
H5S_3	0.566(12)

Atom	Occupancy
C6S_3	0.566(12)
H6S_3	0.566(12)
C7S_3	0.566(12)
H7S_3	0.566(12)
C1S_4	0.434(12)
H1SA_4	0.434(12)
H1SB_4	0.434(12)
H1SC_4	0.434(12)
C2S_4	0.434(12)
C3S_4	0.434(12)
H3S_4	0.434(12)
C4S_4	0.434(12)
H4S_4	0.434(12)
C5S_4	0.434(12)
H5S_4	0.434(12)
C6S_4	0.434(12)
H6S_4	0.434(12)
C7S_4	0.434(12)
H7S_4	0.434(12)

a)

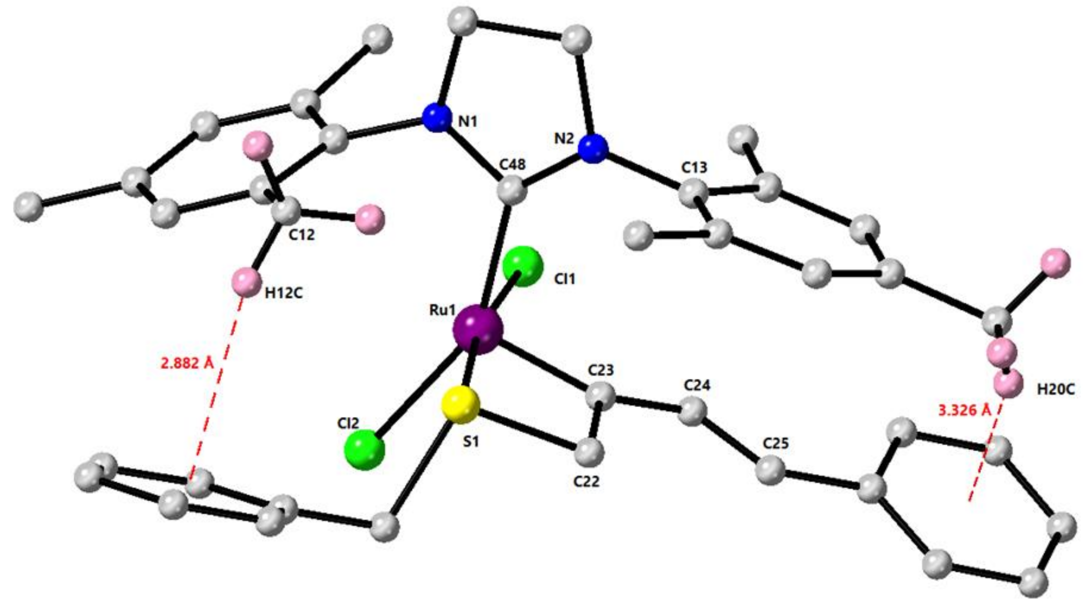

b)

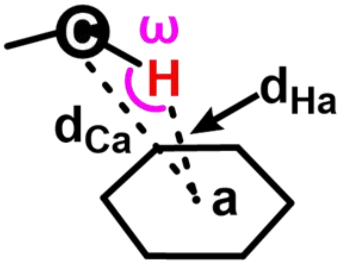

	d_{Ha}(Å)	ω (°)	d_{Ca} (Å)
H12c	2.88	165.7	3.96
H20c	3.33	154.8	4.33

Figure S22. a) Ball and stick model of **11** showing distances between mesityl methyl hydrogens H10c and H20b and the phenyl rings b) Table of representative angles and distances associated with H-π interactions.

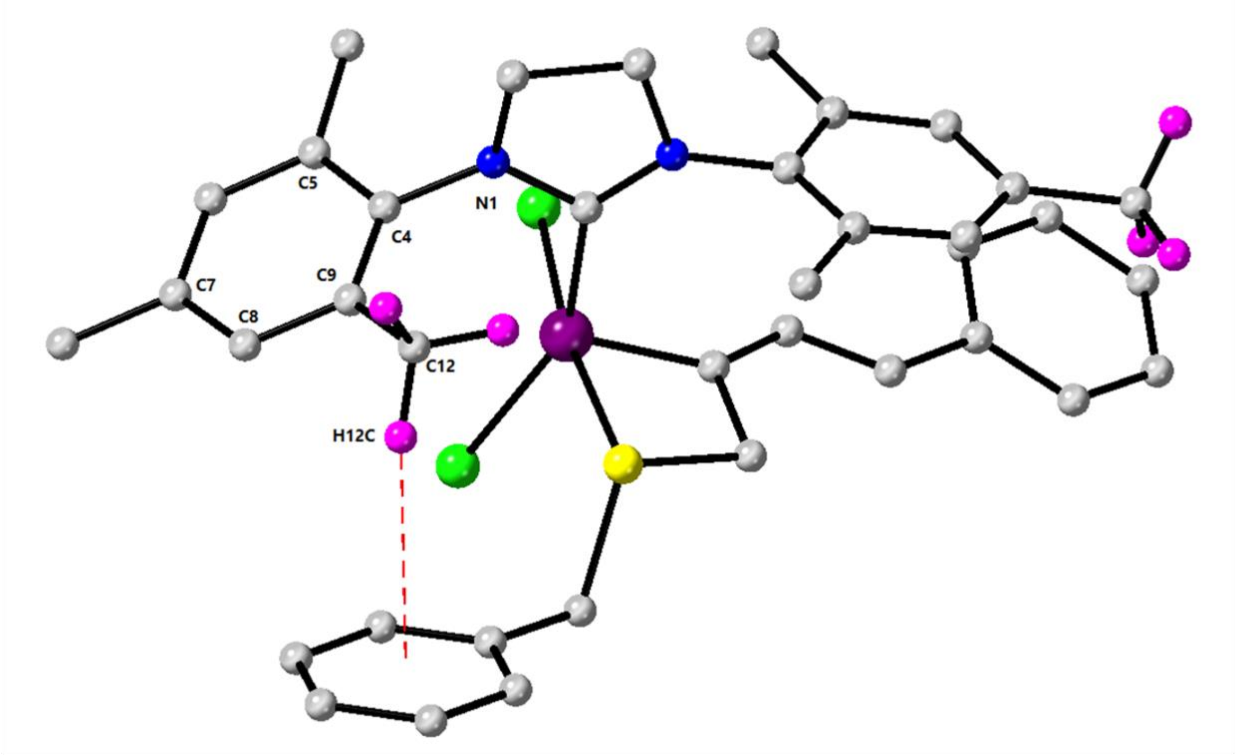

Atoms	Torsional angle (°)
N1-C4-C5-C10	8.8
C9-C4-C5-C10	-168.5
C7-C6-C5-C10	170.4

Figure S23. Ball and stick model of **11** and selected torsional angles of the mesityl methyl (C12).

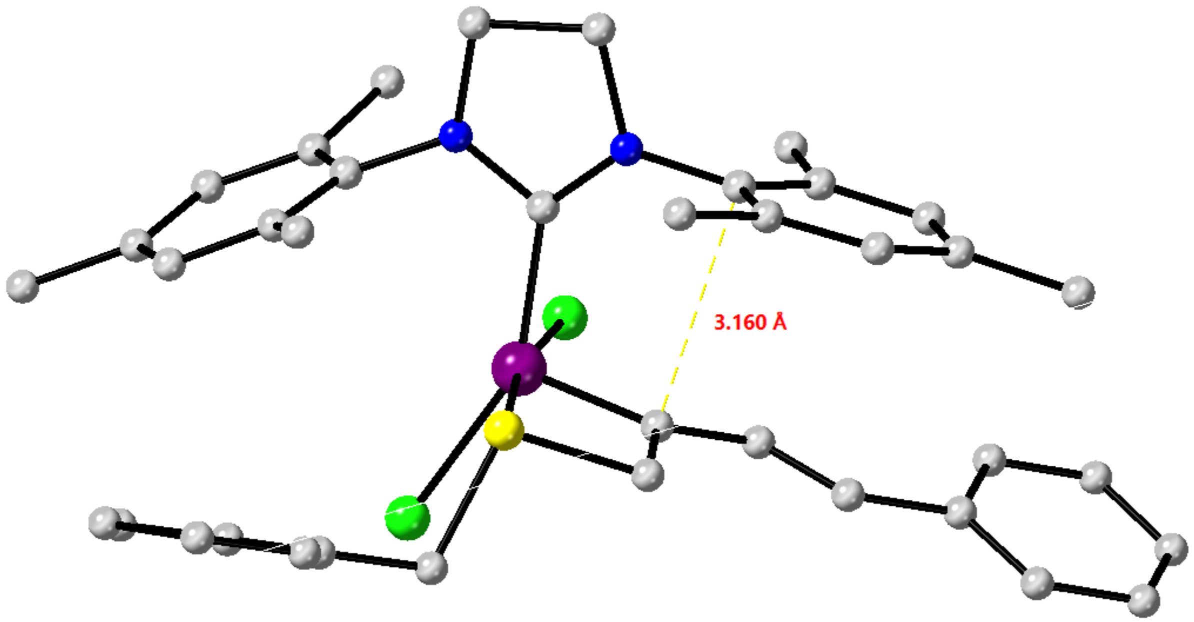

Figure S24. Ball and stick model of **11** showing the distance between C*ipso* (C13) of the mesityl and alkylidene carbon (C23).

6. UV-Vis Spectrophotometry Data of Complexes 10 and 11

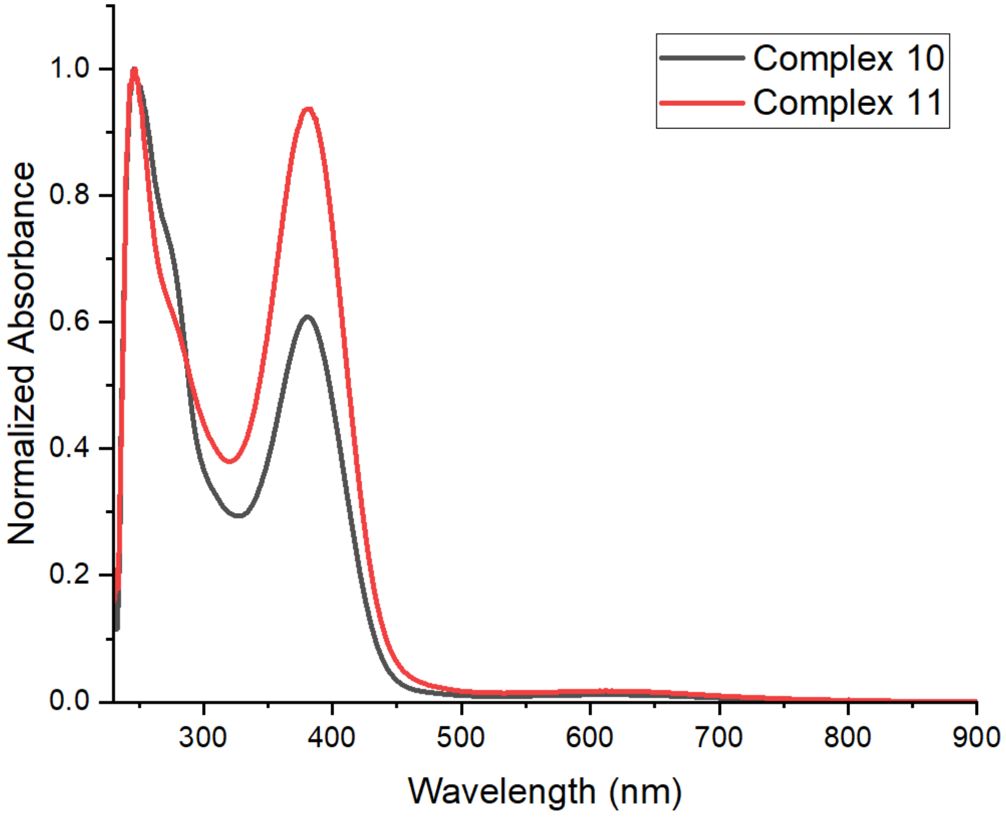

Figure S25. UV-Vis spectra of complexes **10** and **11**.

7. General NMR and GPC Data of Intermediates and Metathesis Reactions

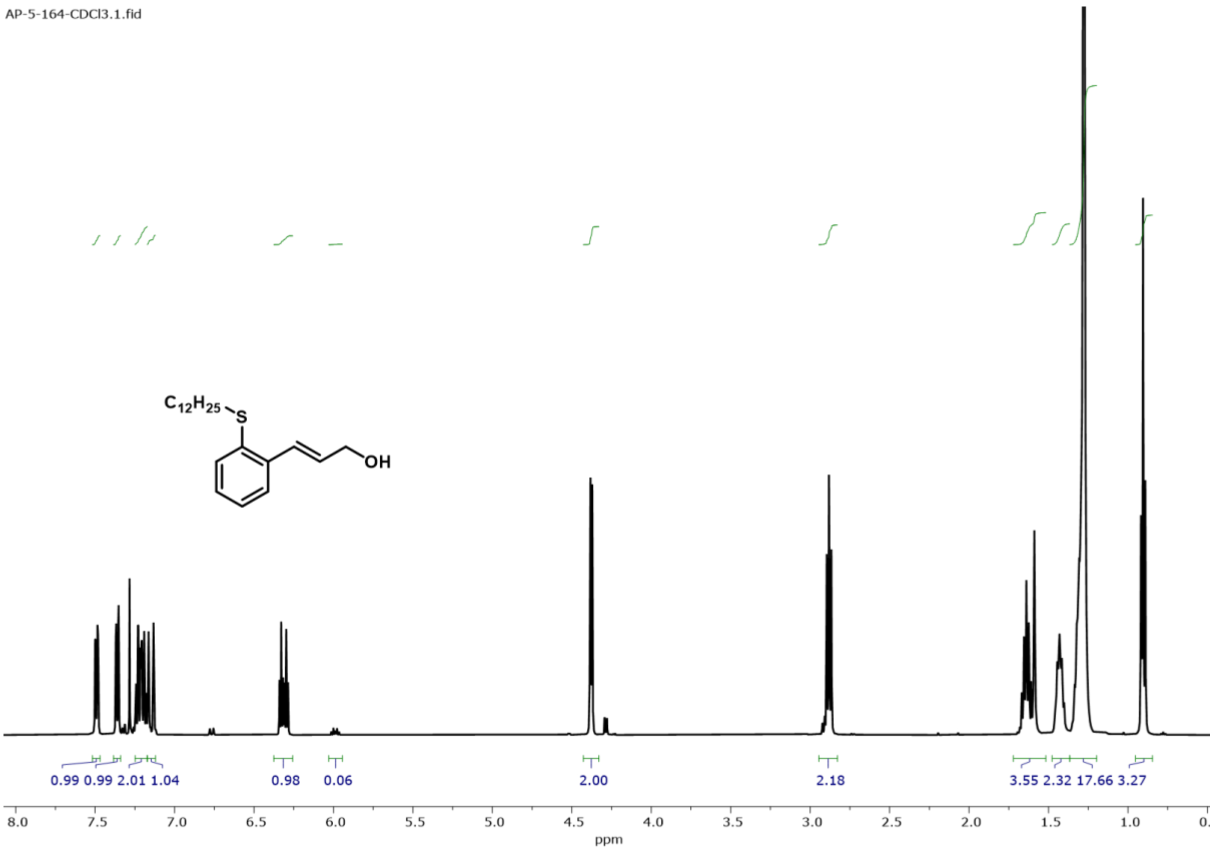

Figure S26. ^{1}H NMR of **S2** in $CDCl_3$.

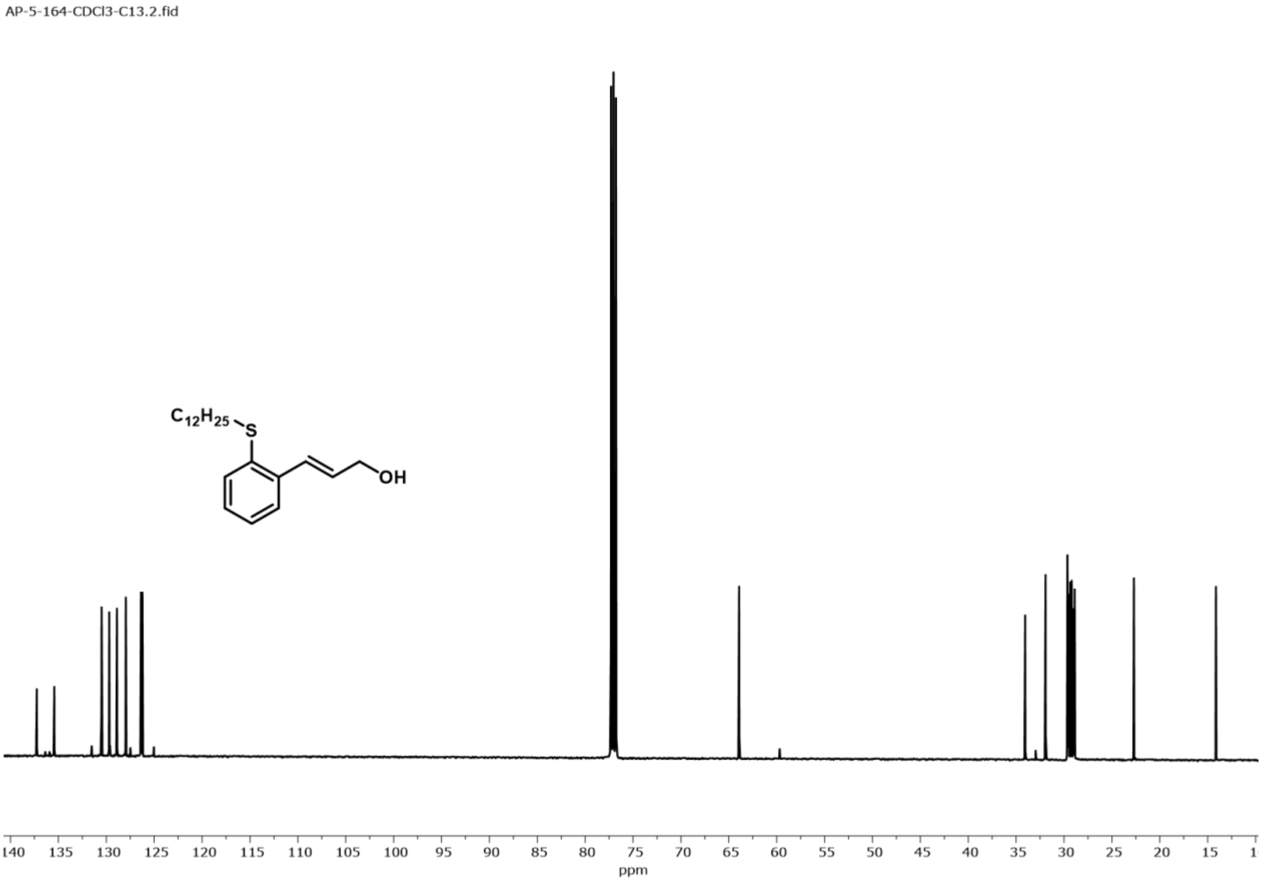

Figure S27. ^{13}C NMR of **S2** in $CDCl_3$.

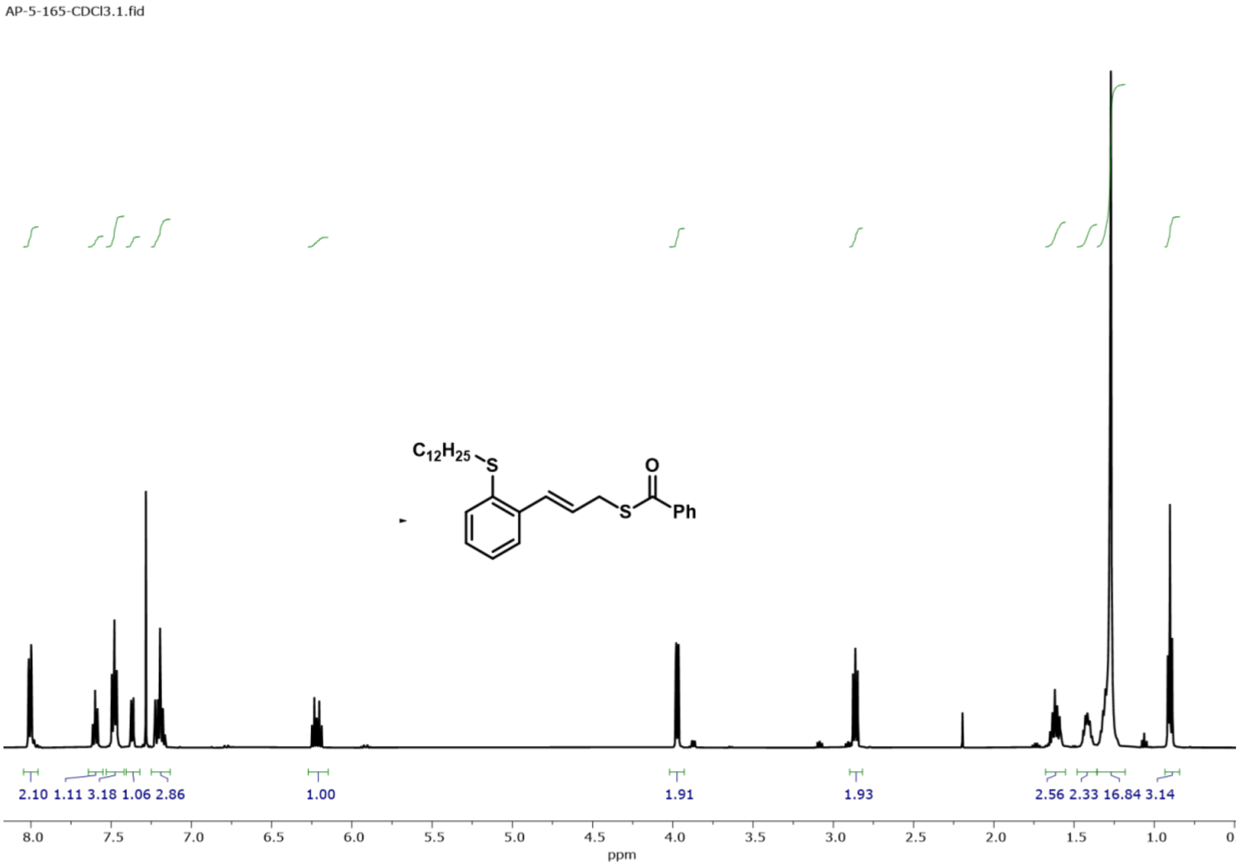

Figure S28. ^{1}H NMR of **S3** in $CDCl_3$.

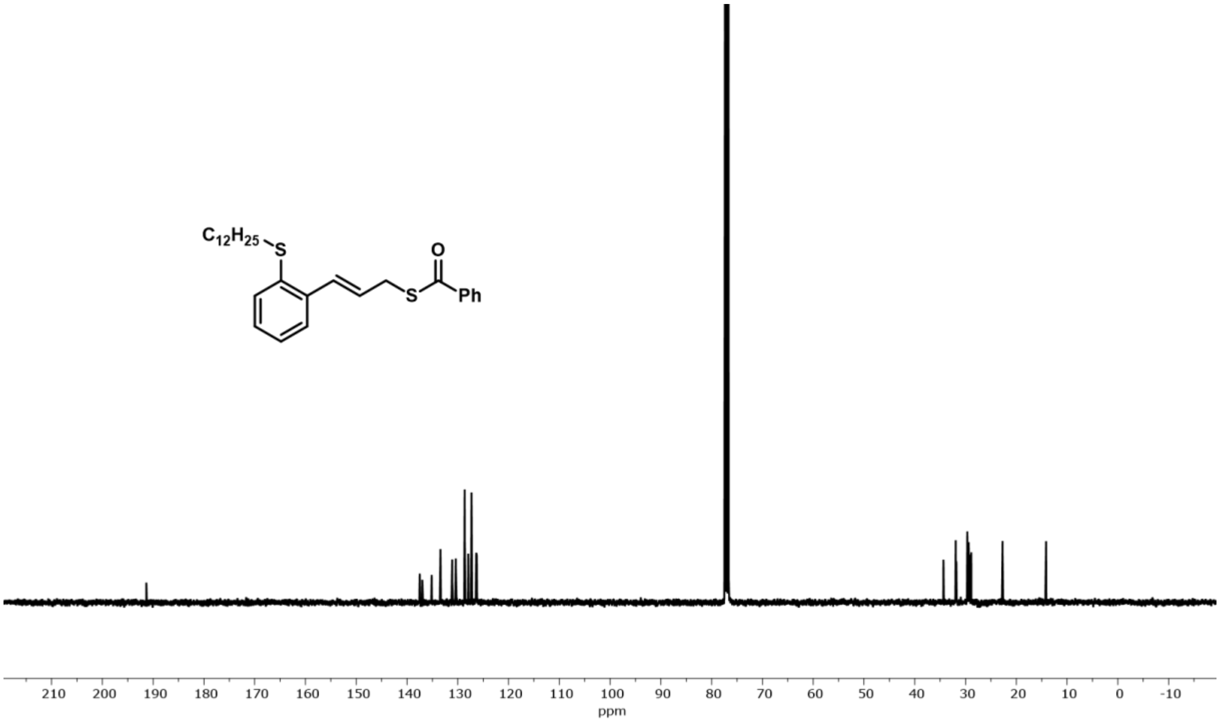

Figure S29. ^{13}C NMR of **S3** in $CDCl_3$.

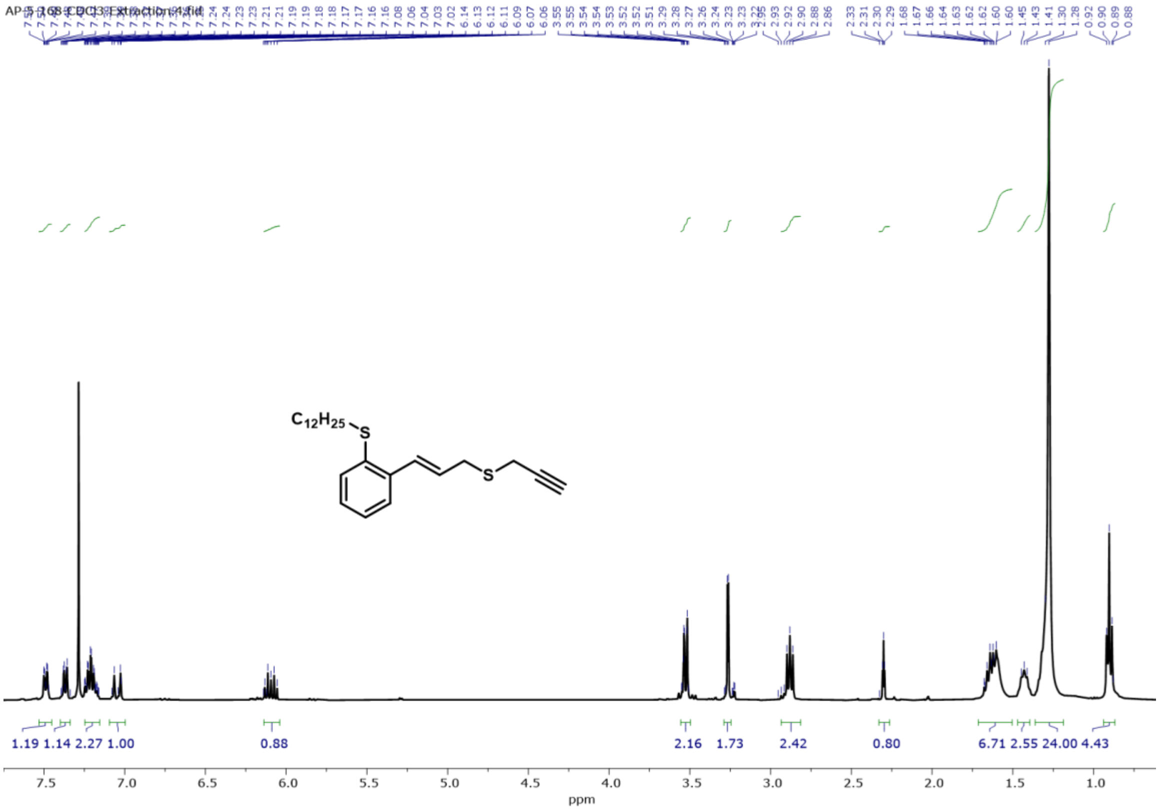

Figure S30. ^{1}H NMR of **12** in $CDCl_3$.

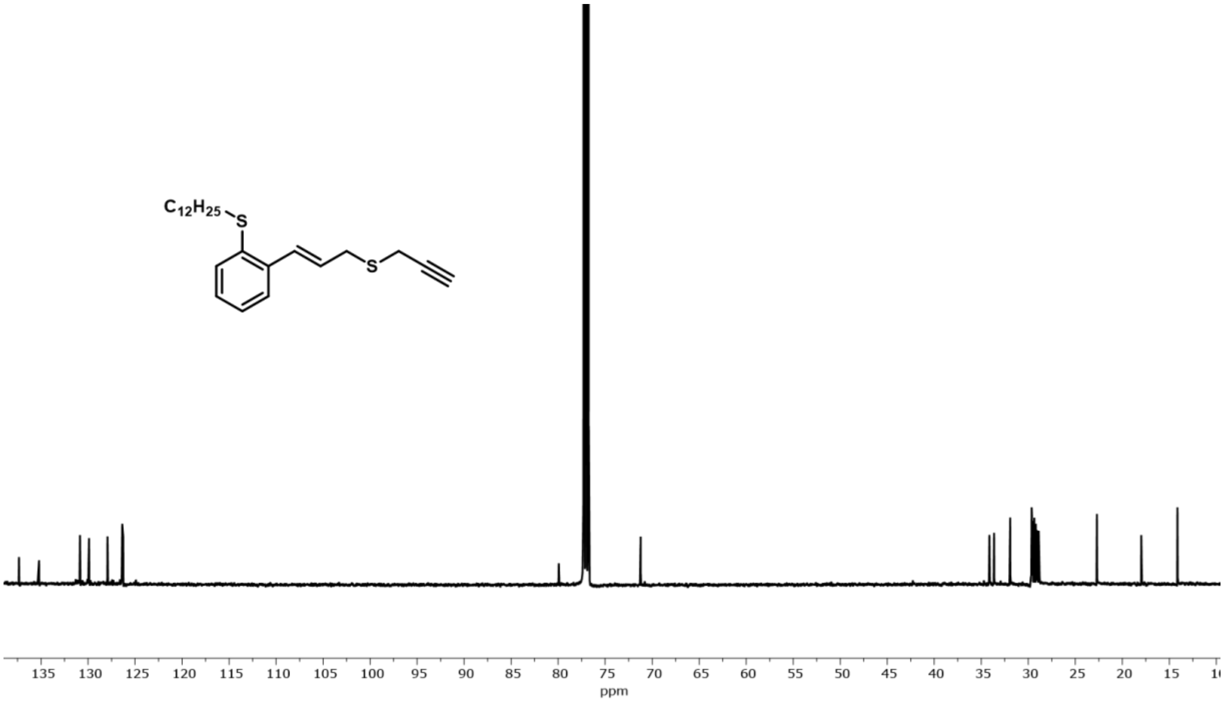

Figure S31. ^{13}C NMR of **12** in $CDCl_3$.

^{1}H NMRs of 17 Ring-Opening Metathesis Polymerizations

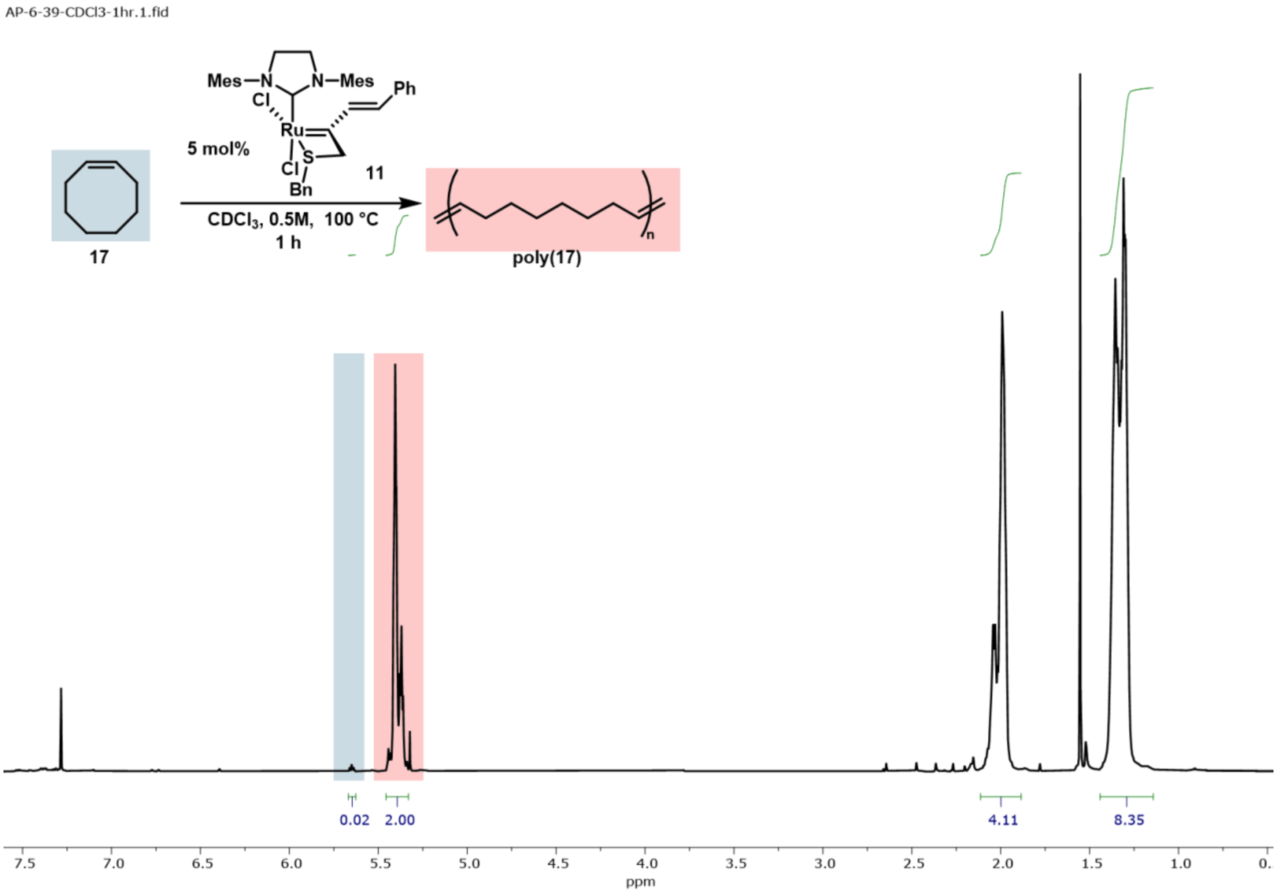

Figure S32. ^{1}H NMR of the ROMP of **17** in $CDCl_3$ at 100 °C

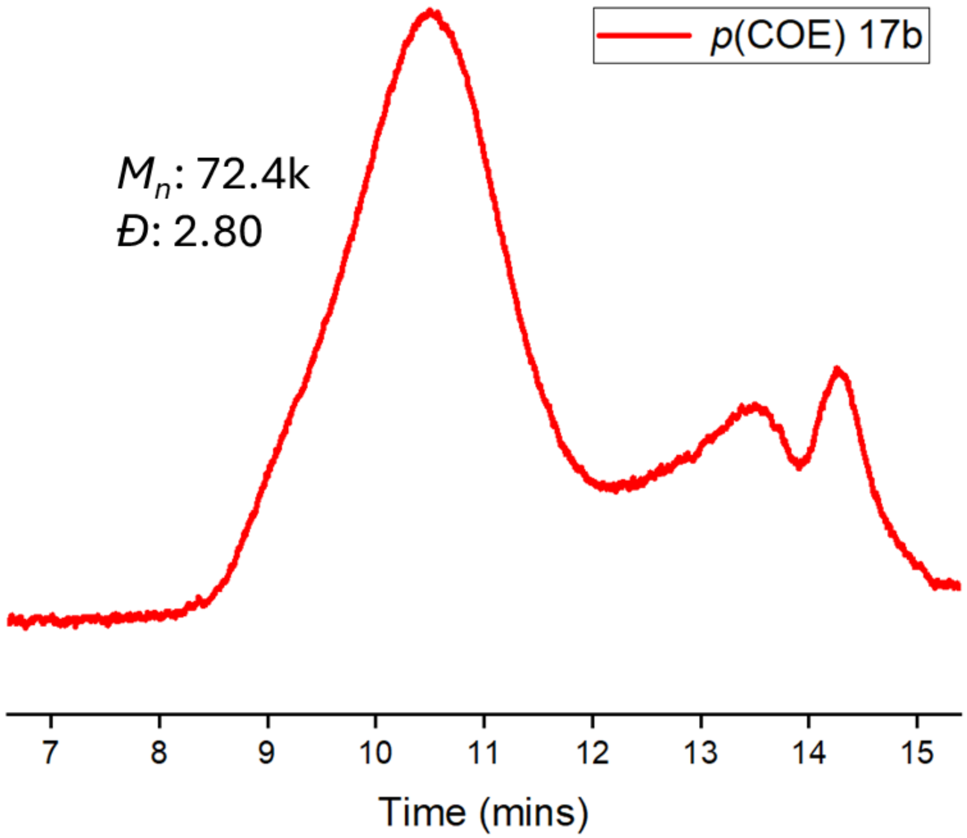

Figure S33. SEC trace of the ROMP of **17** in $CDCl_3$ at 100 °C

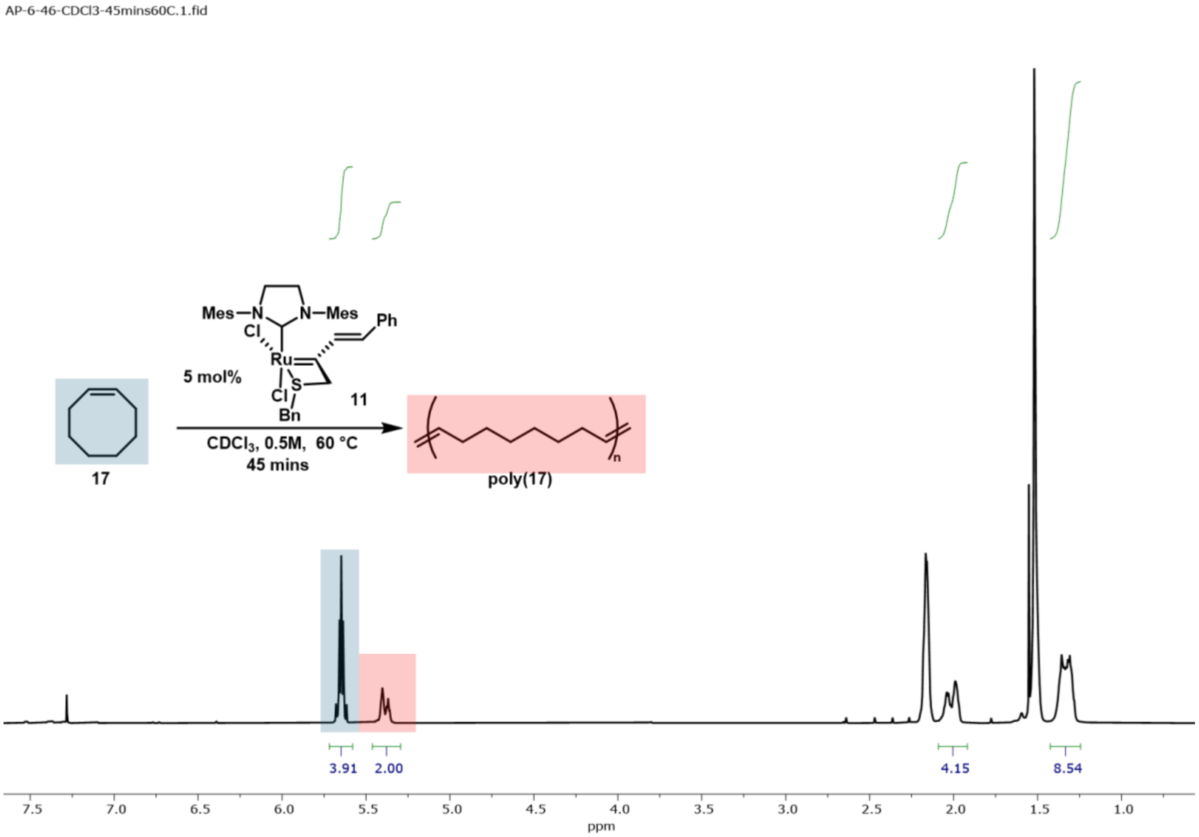

Figure S34. ^{1}H NMR of the ROMP of **17** in $CDCl_3$ at 60 °C for 45 mins.

AP-6-47-Tol-45mins60C.2.fid

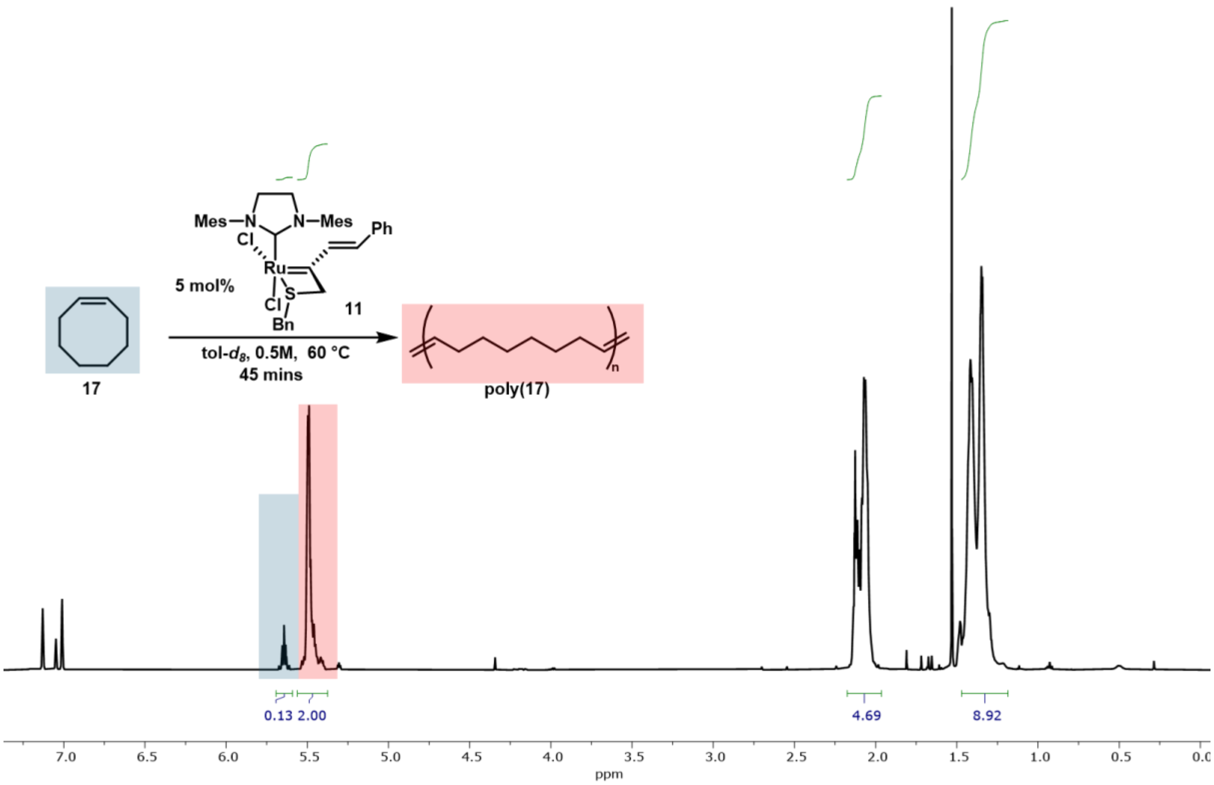

Figure S35. ^{1}H NMR of the ROMP of **17** in tol-d_8 at 60 °C for 45 mins.

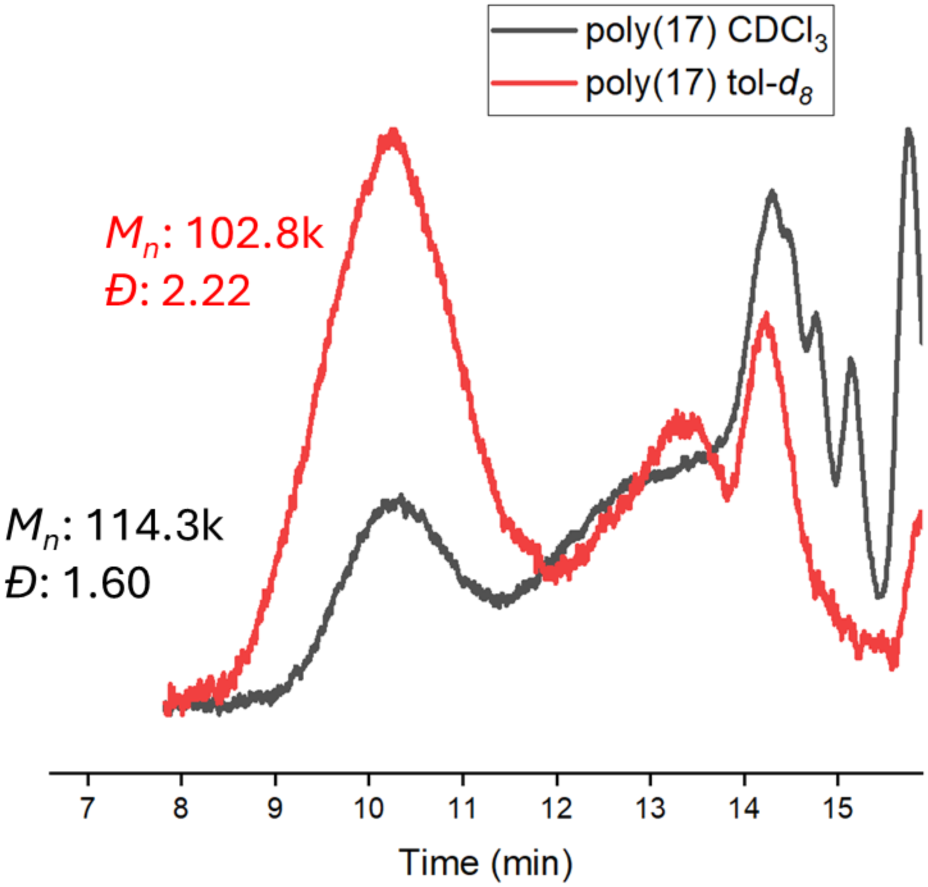

Figure S36. SEC trace of the ROMP of **17** in $CDCl_3$ and tol-d_8 at 60 °C for 45 mins.

^{1}H NMRs of 18a Ring-Closing Metathesis Reactions

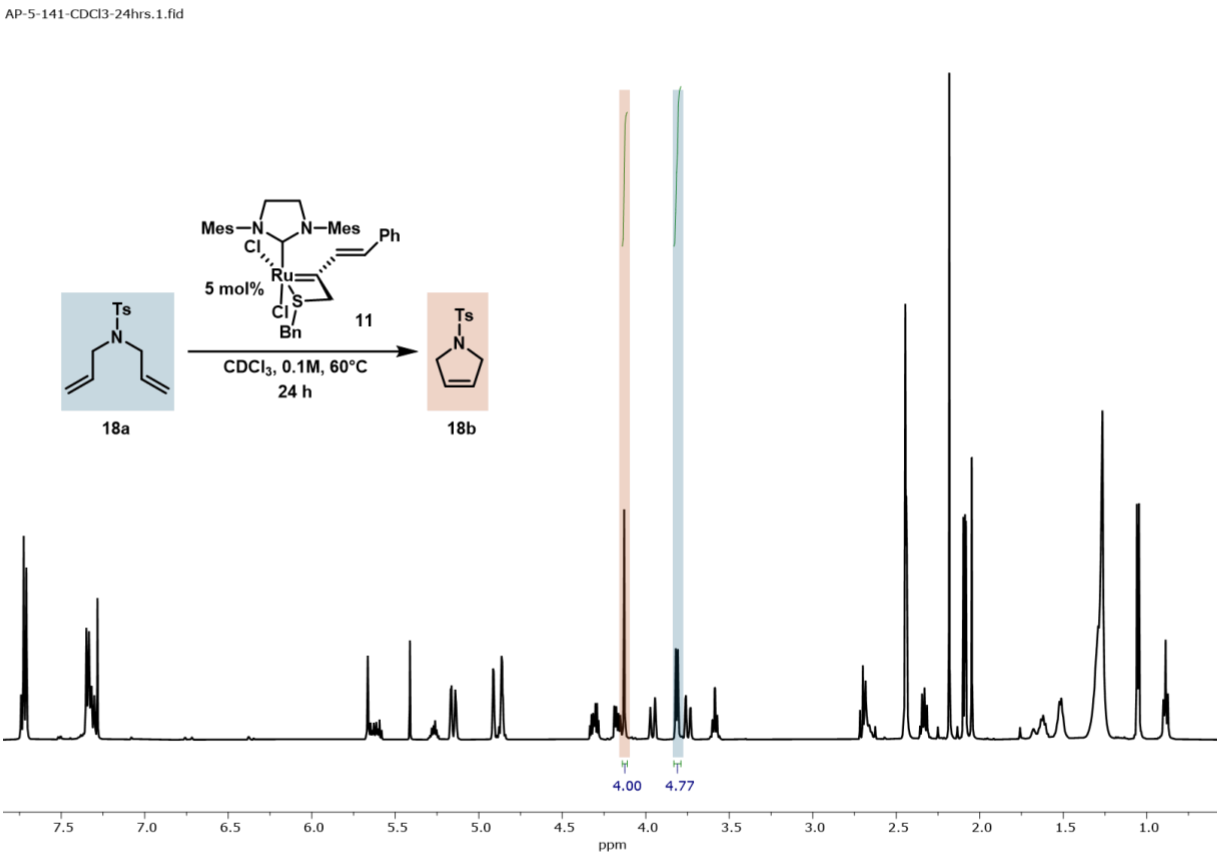

Figure S37. ^{1}H NMR of the RCM of **18a** in $CDCl_3$ at 60 °C for 24 h.

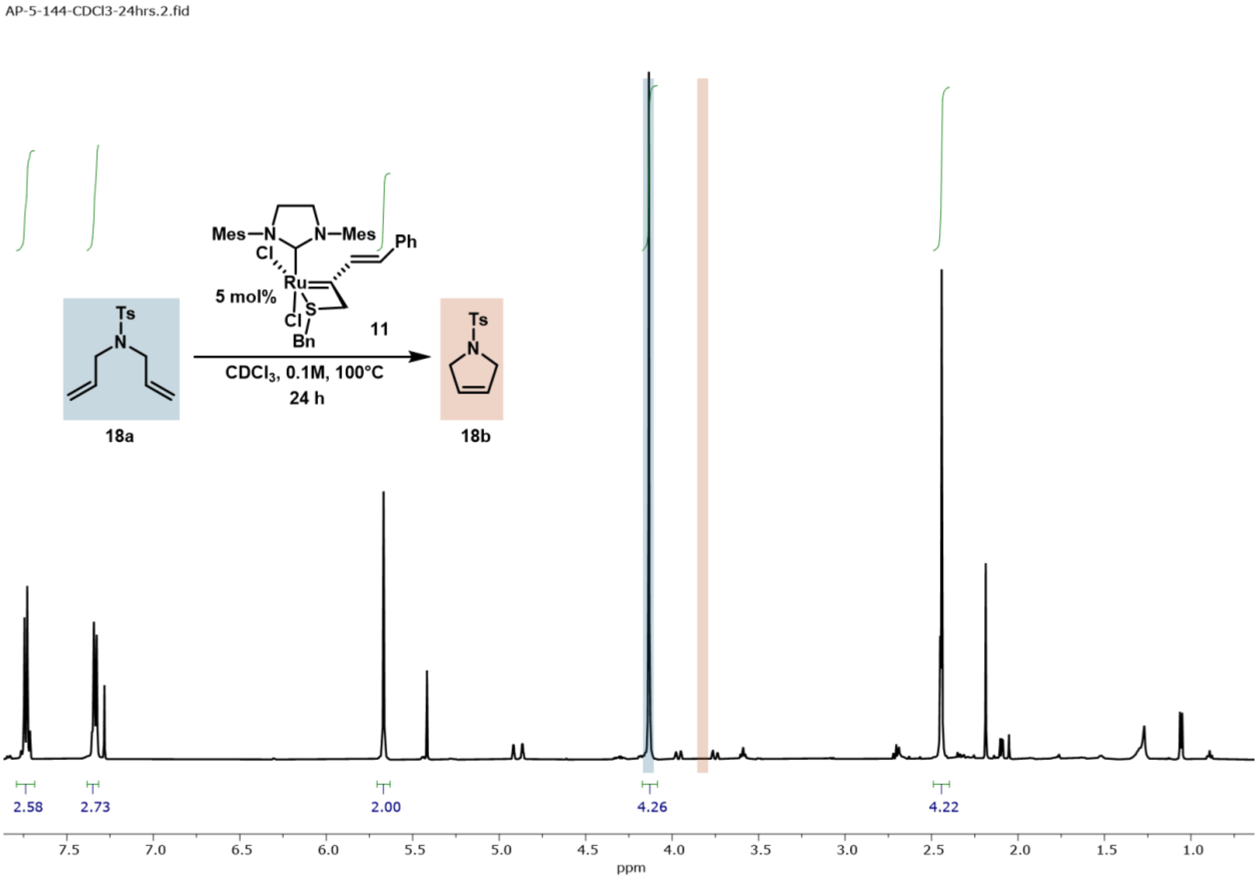

Figure S38. ^{1}H NMR of the RCM of **18a** in $CDCl_3$ at 100 °C for 24 h.

AP-5-154-CDCl3-1hr.2.fid

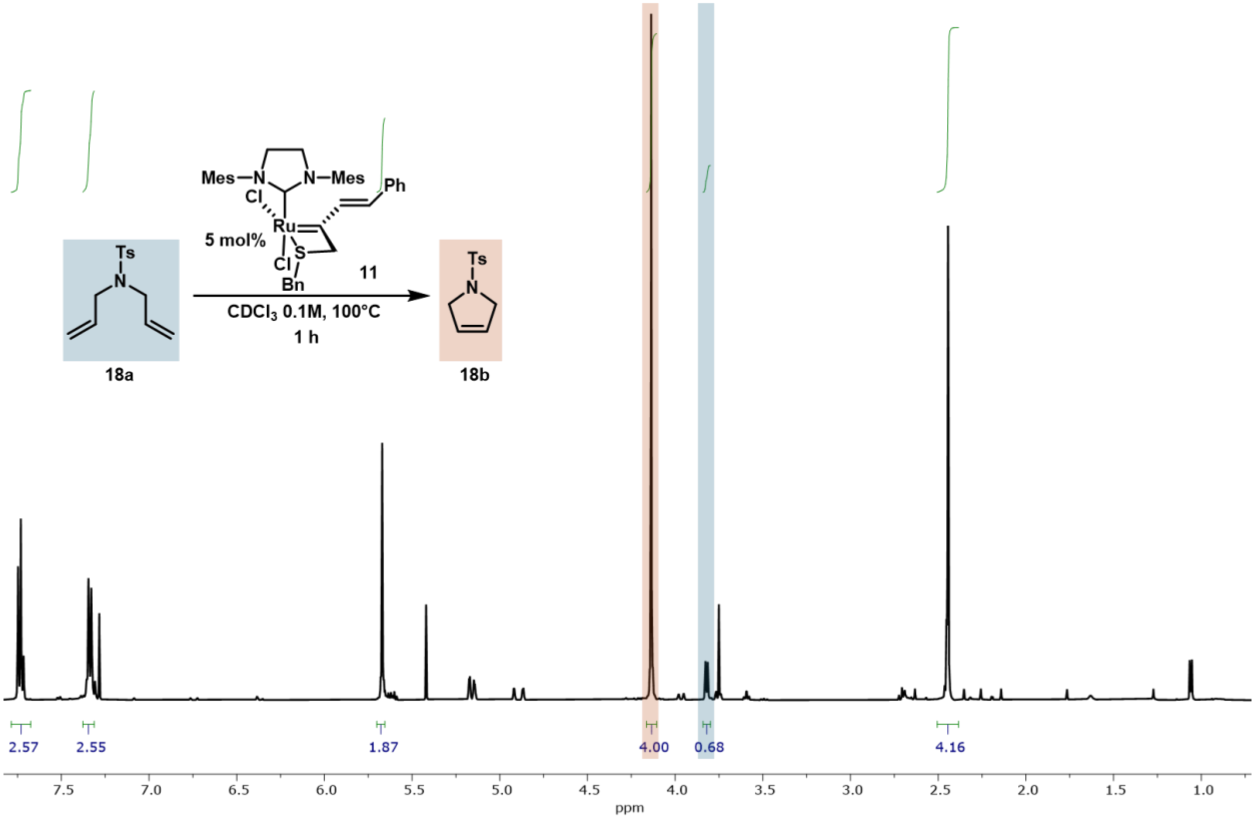

Figure S39. ^{1}H NMR of the RCM of **18a** in $CDCl_3$ at 100 °C for 1 h.

AP-5-163-CDCl3-300nm-1hr.1.fid

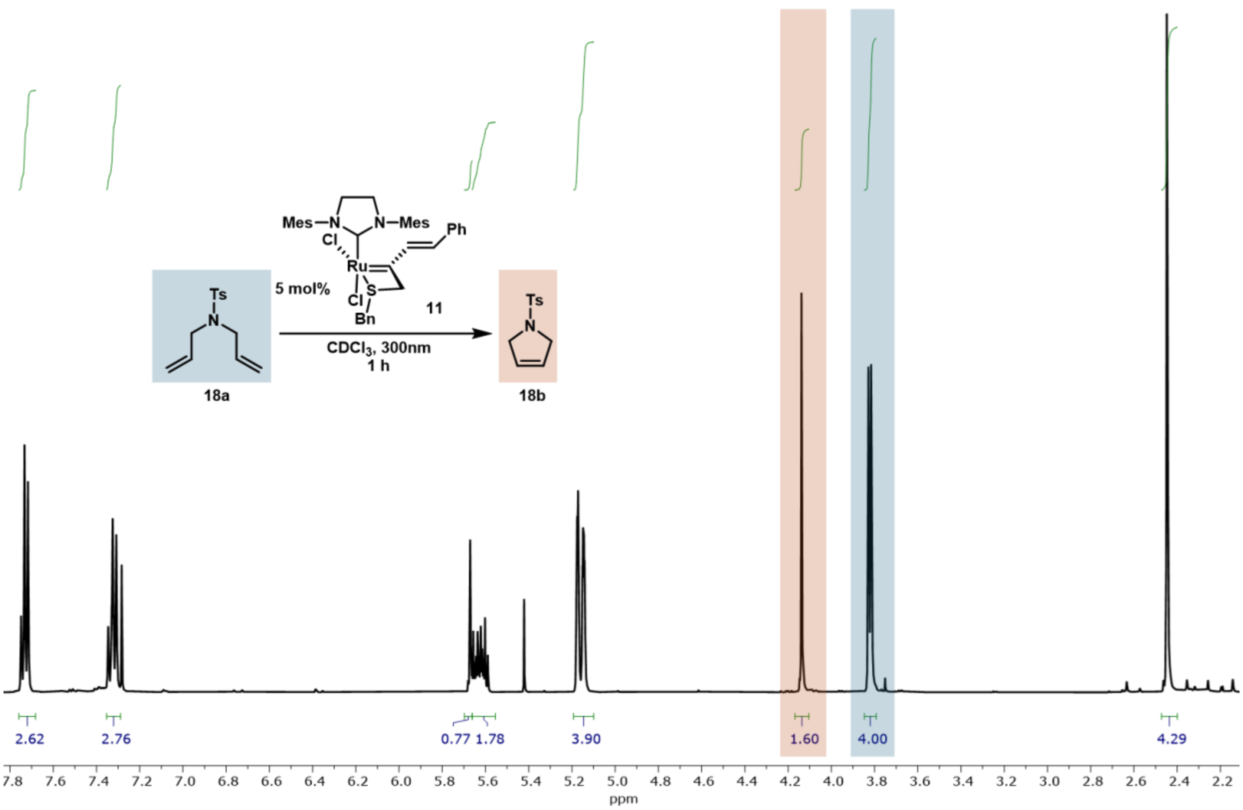

Figure S40. ^{1}H NMR of the RCM of **18a** in $CDCl_3$ for 1 h under 300 nm light.

AP-6-36-CDCl3-1hr.2.fid

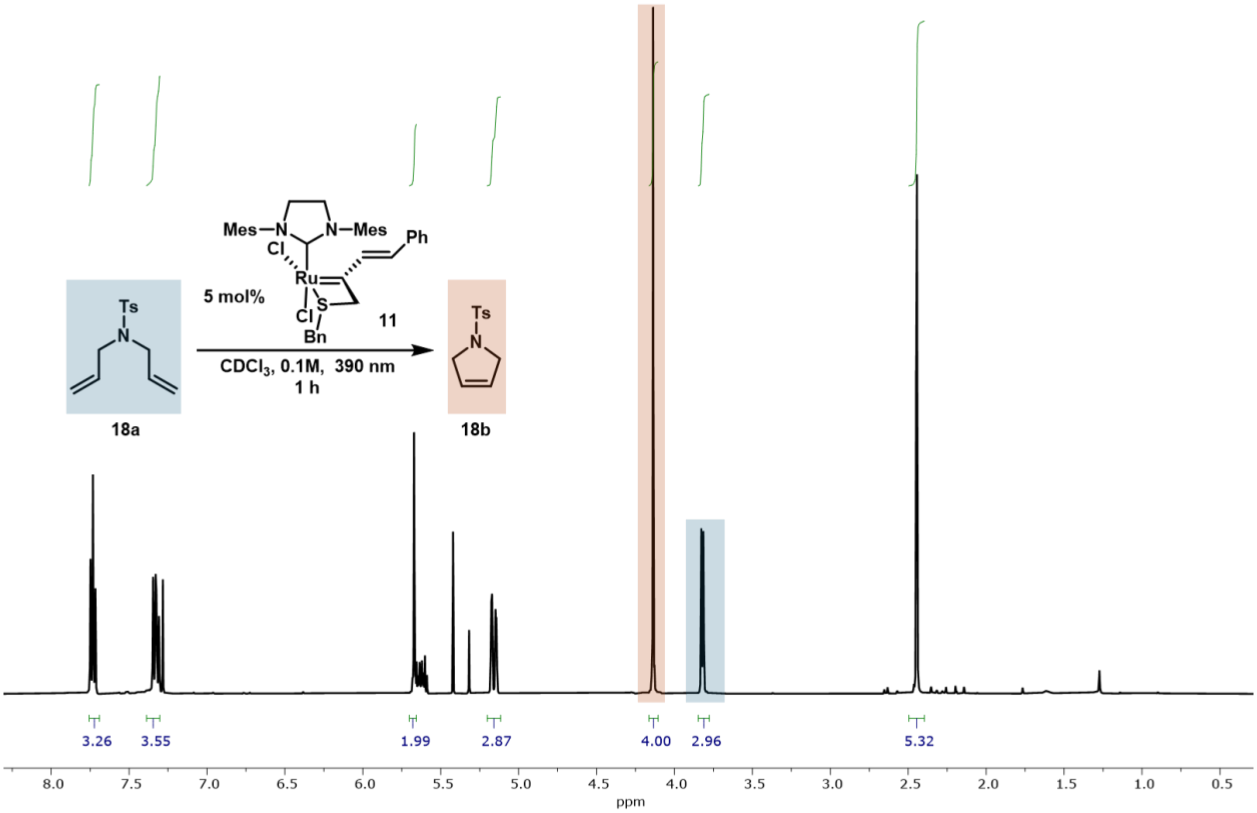

Figure S41. ^{1}H NMR of the RCM of **18a** in $CDCl_3$ for 1 h under 390 nm light.

AP-5-153-CDCl3-1hr.1.fid

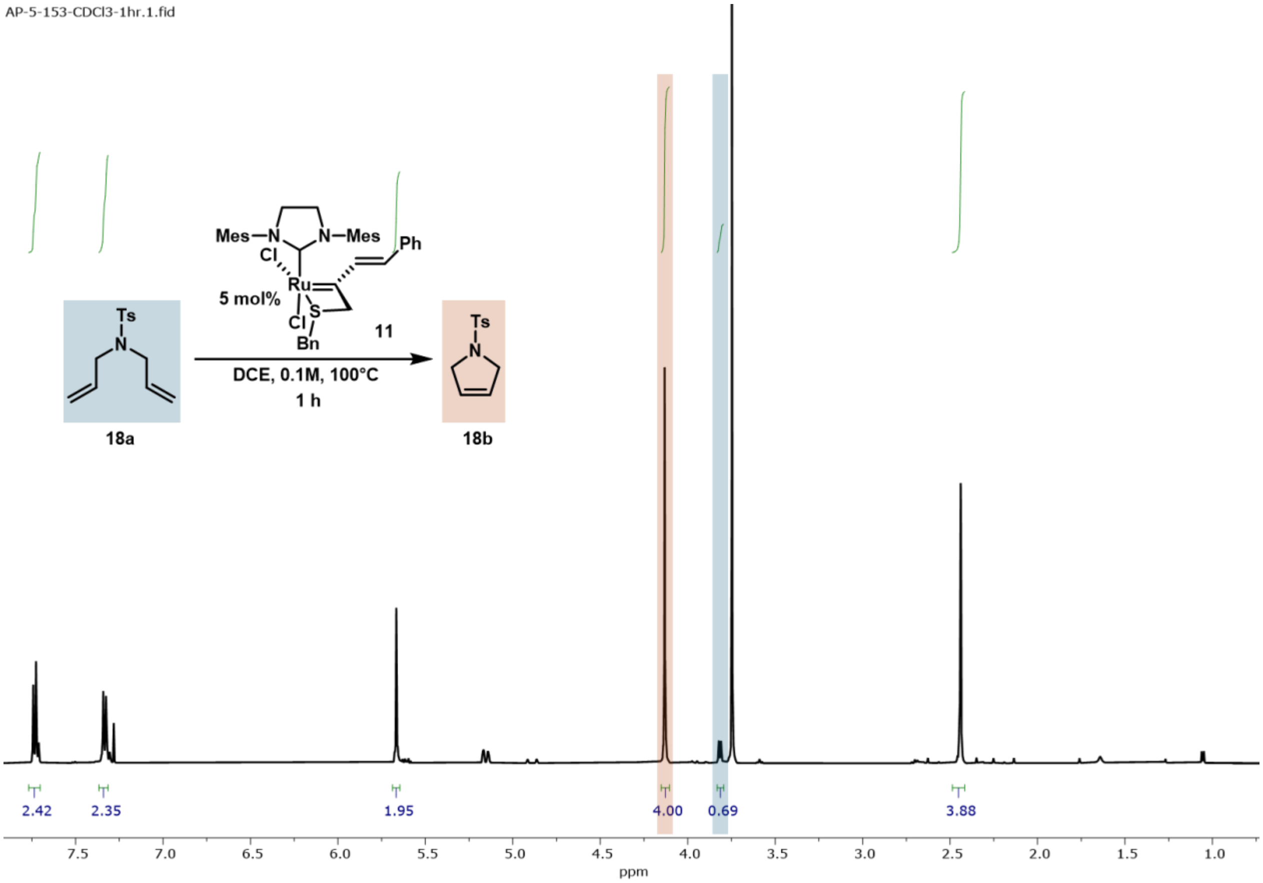

Figure S42. ^{1}H NMR of the RCM of **18a** in DCE at 100 °C for 1 h.

AP-5-161-Tol-100C-20mins.1.fid

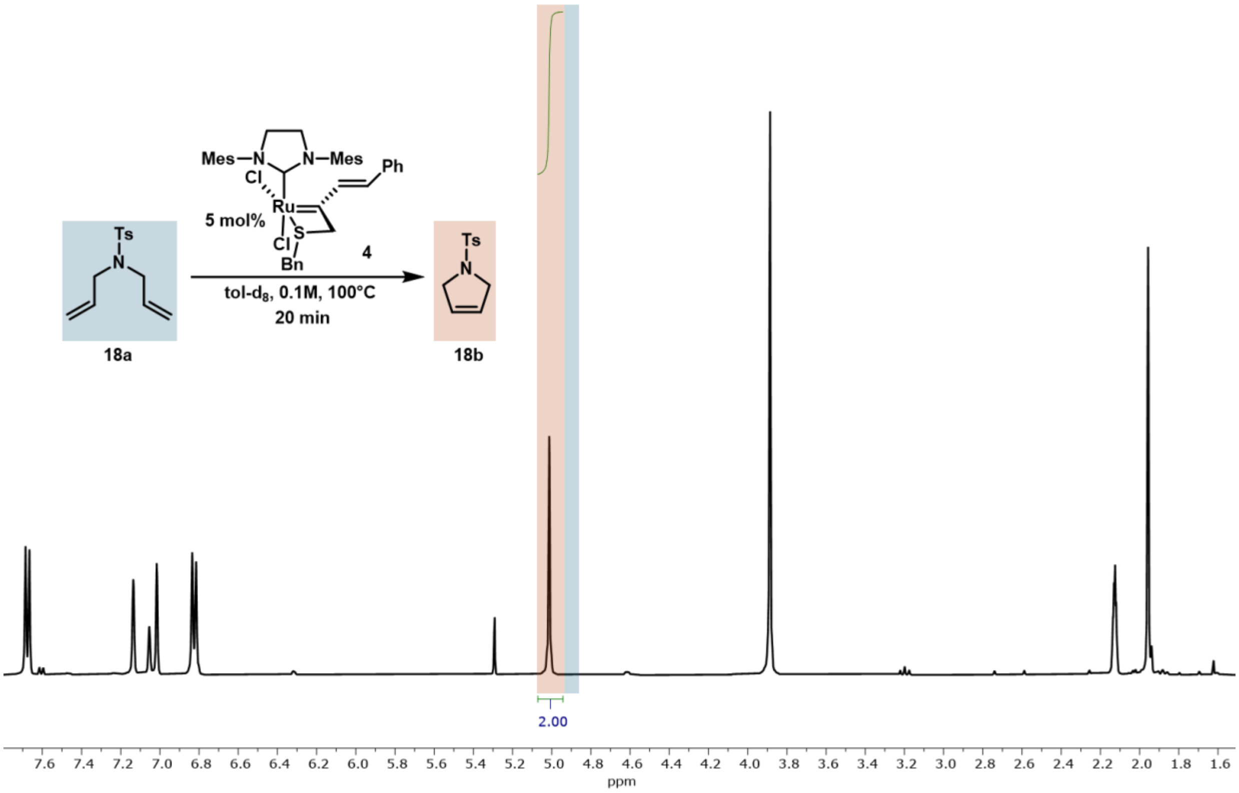

Figure S43. ^{1}H NMR of the RCM of **18a** in tol-d_8 at 100 °C for 20 mins.

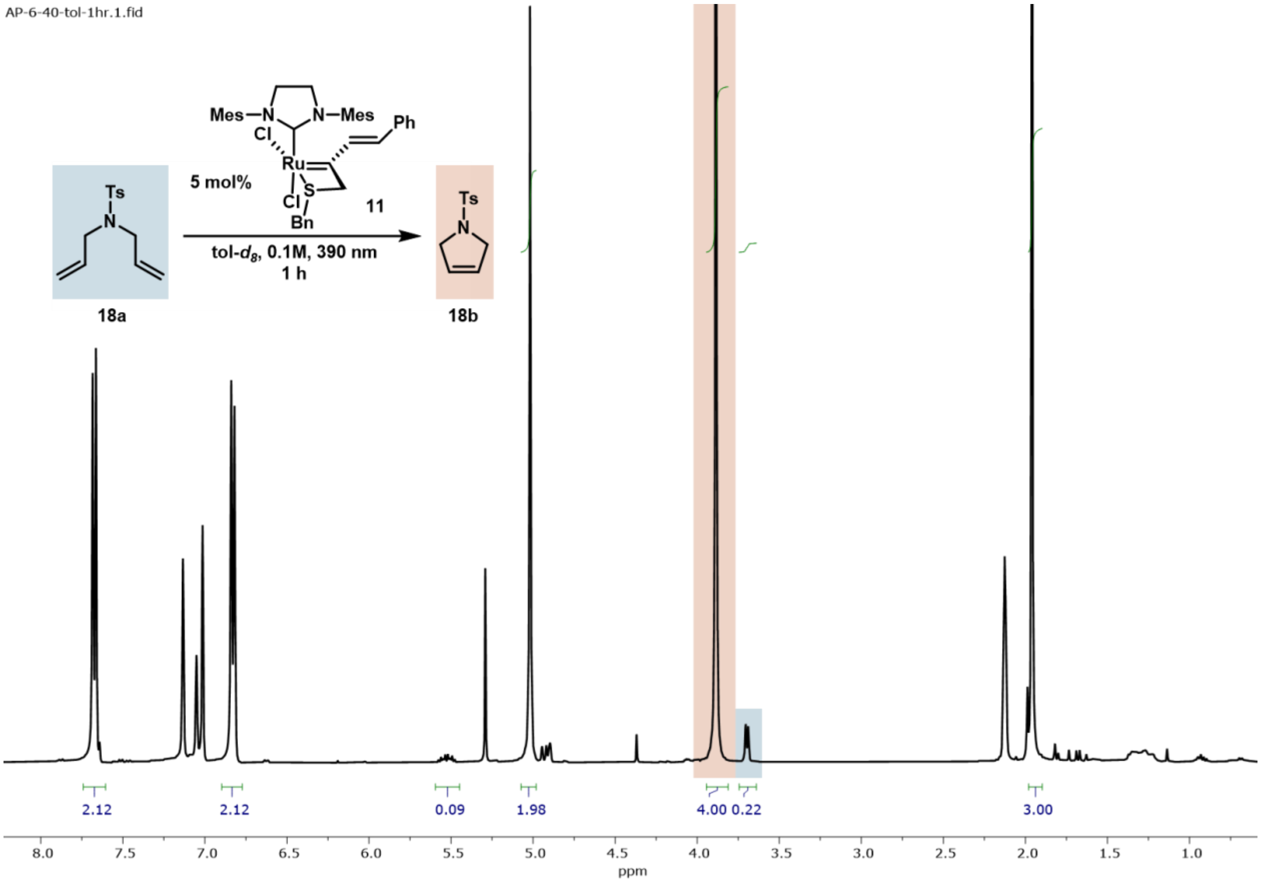

Figure S44. ^{1}H NMR of the RCM of **18a** in tol-d_8 for 1 h under 390 nm light.

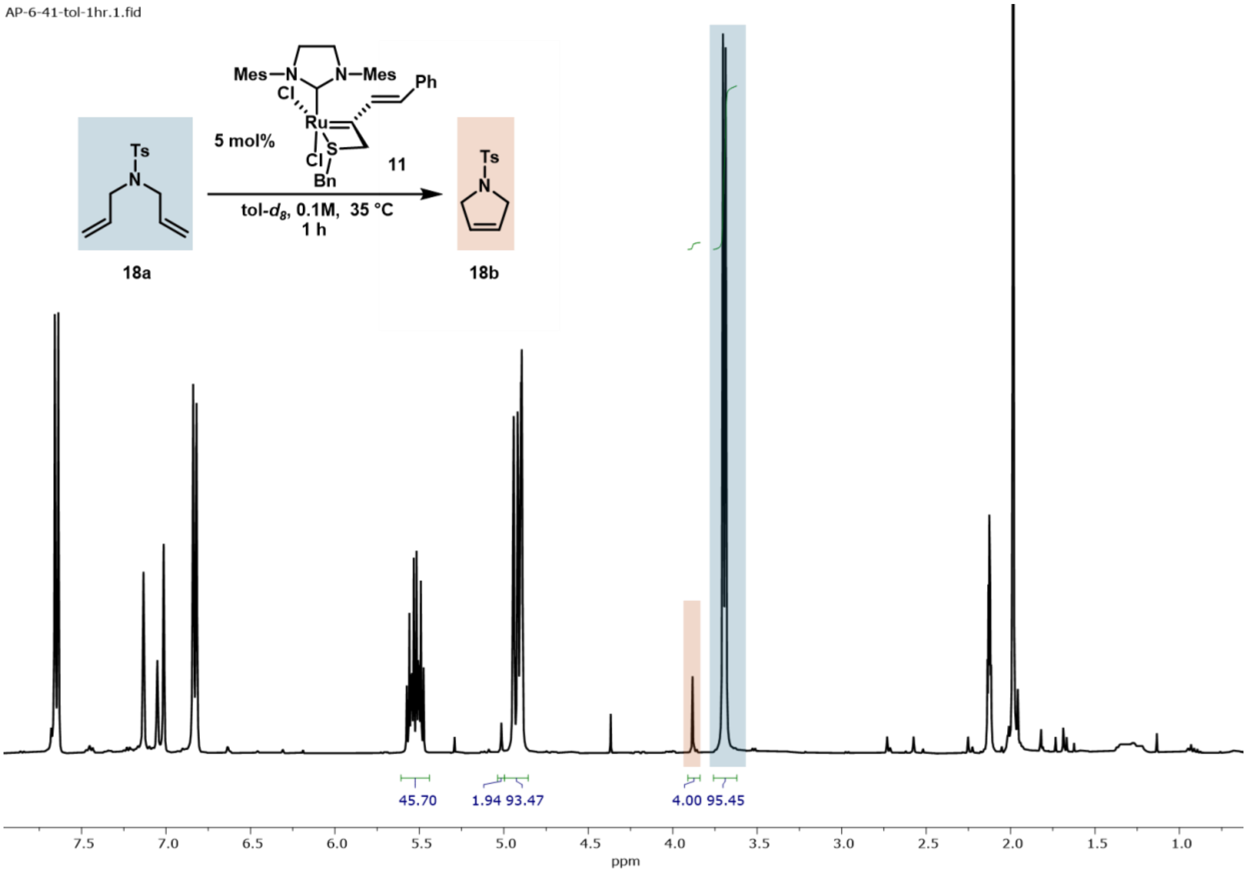

Figure S45. ^{1}H NMR of the RCM of **18a** in tol-d_8 at 35 °C for 1 h.

^{1}H NMRs of 19a Ring-Closing Metathesis Reactions

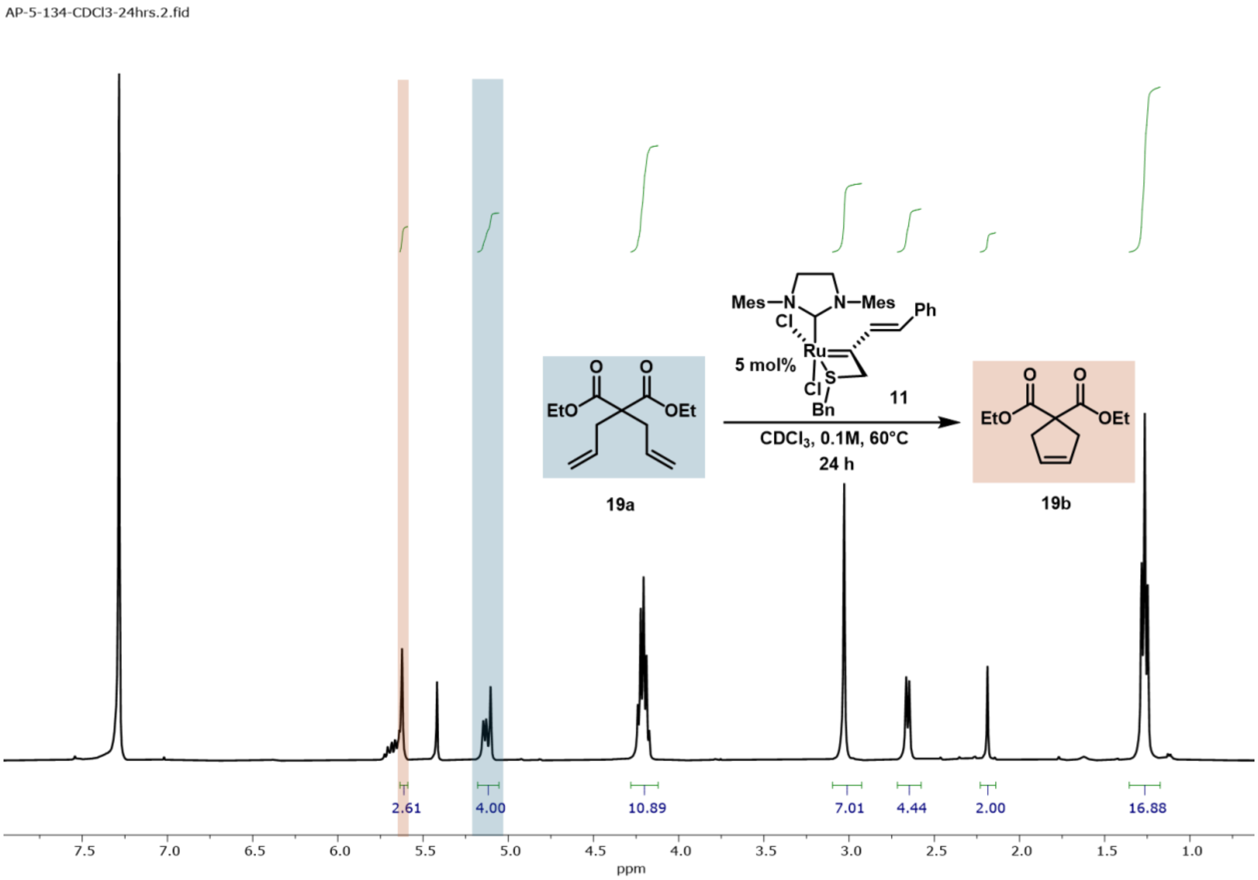

Figure S46. ^{1}H NMR of the RCM of **19a** in $CDCl_3$ at 60 °C for 24 h.

AP-5-146-CDCl3-24hrs.2.fid

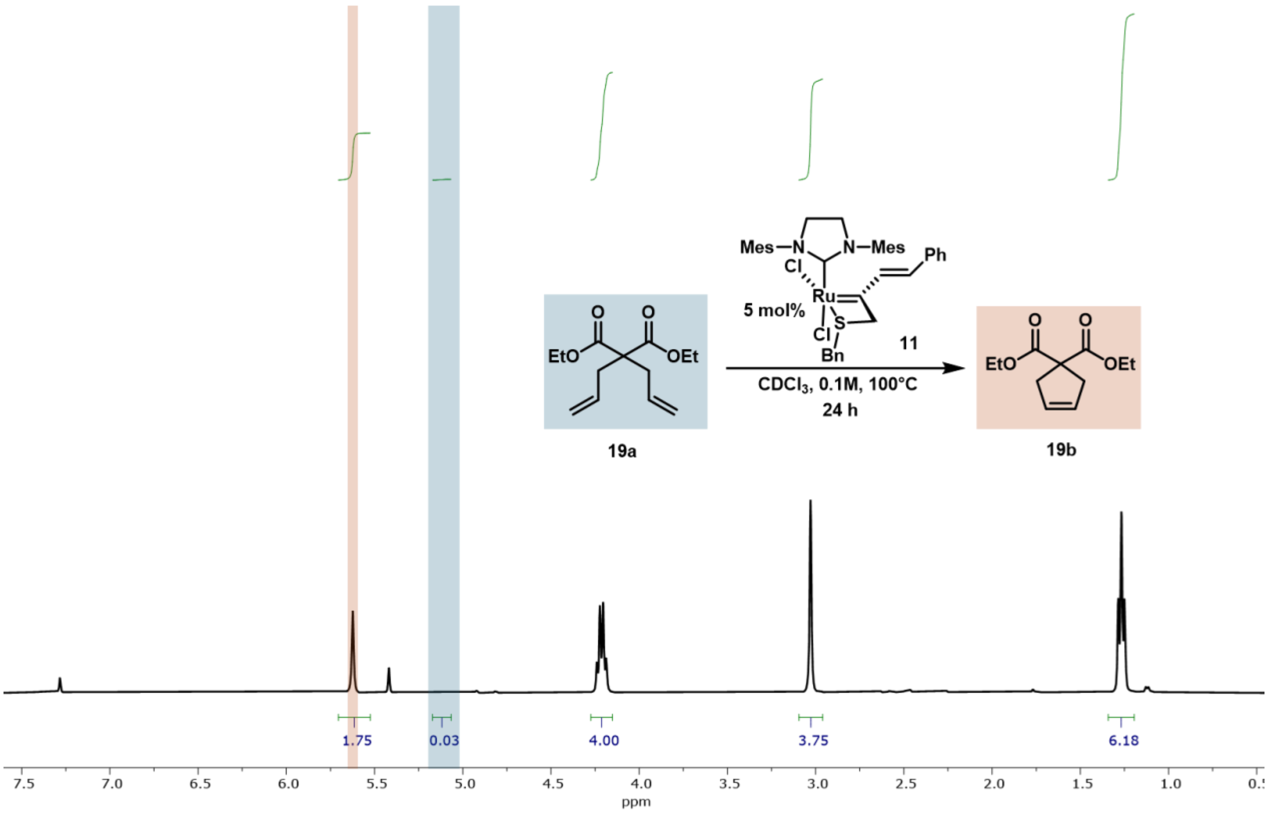

Figure S47. ^{1}H NMR of the RCM of **19a** in $CDCl_3$ at 100 °C for 24 h.

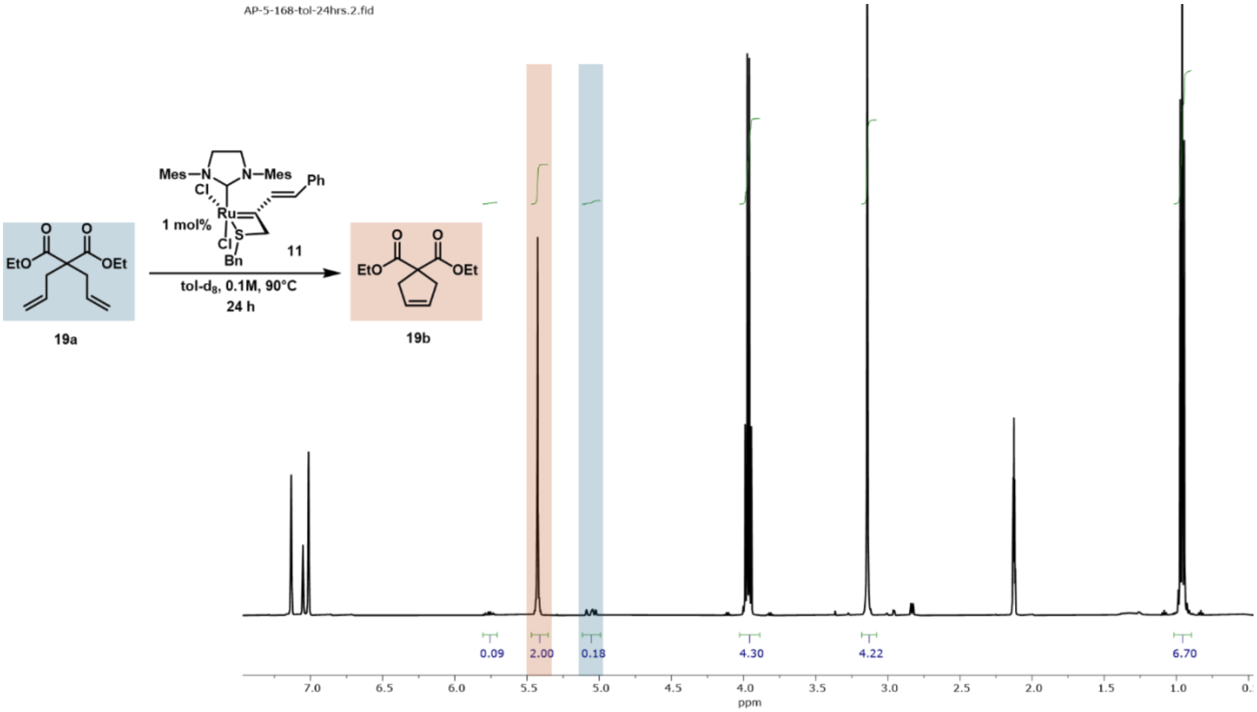

Figure S48. ^{1}H NMR of the RCM of **19a** in tol-d_8 at 90 °C for 24 h.

^{1}H NMRs of 20a Cross Metathesis Reactions

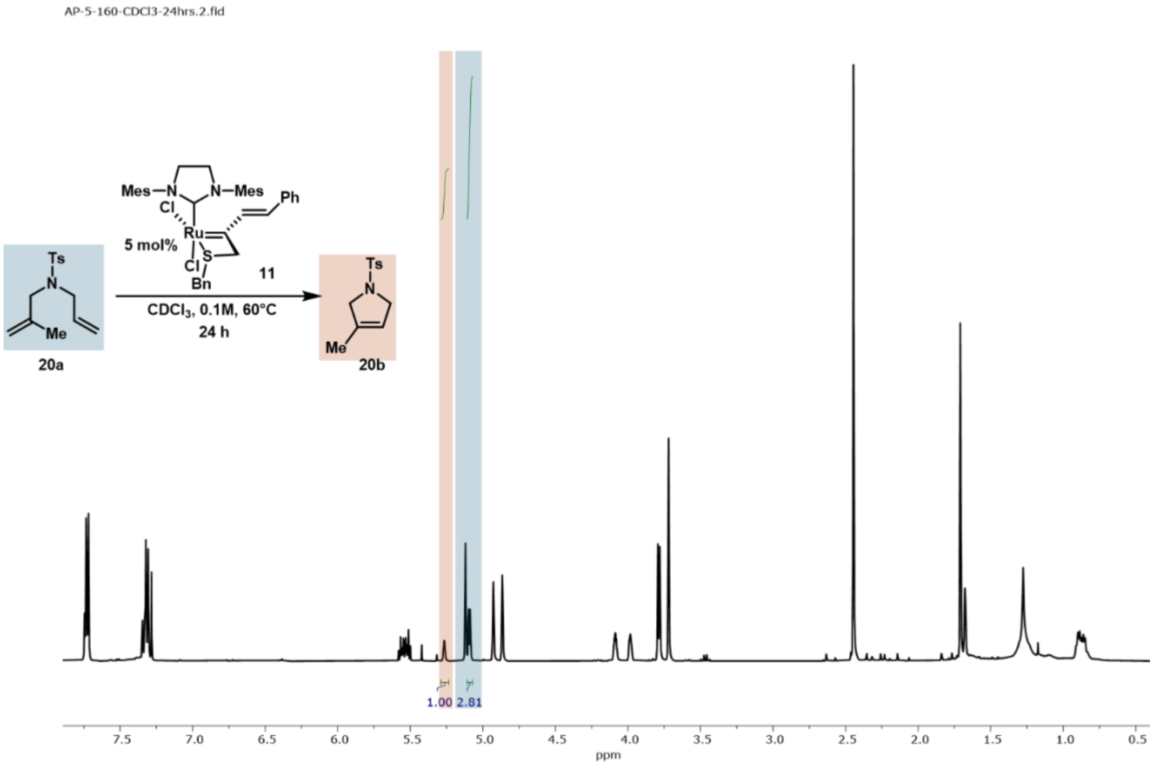

Figure S49. ^{1}H NMR of the RCM of **20a** in $CDCl_3$ at 60 °C for 24 h.

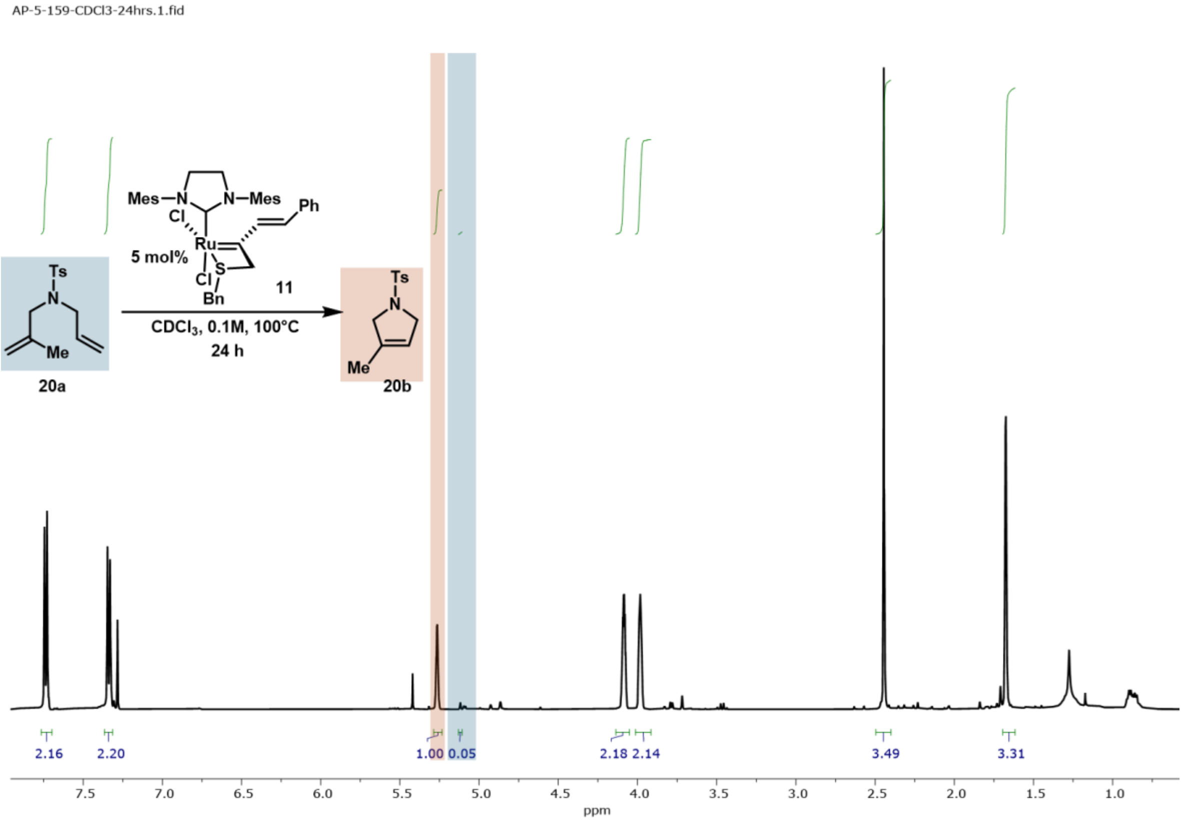

Figure S50. ^{1}H NMR of the RCM of **20a** in $CDCl_3$ at 100 °C for 24 h.

^{1}H NMRs of 21a Ring-Closing Metathesis Reactions

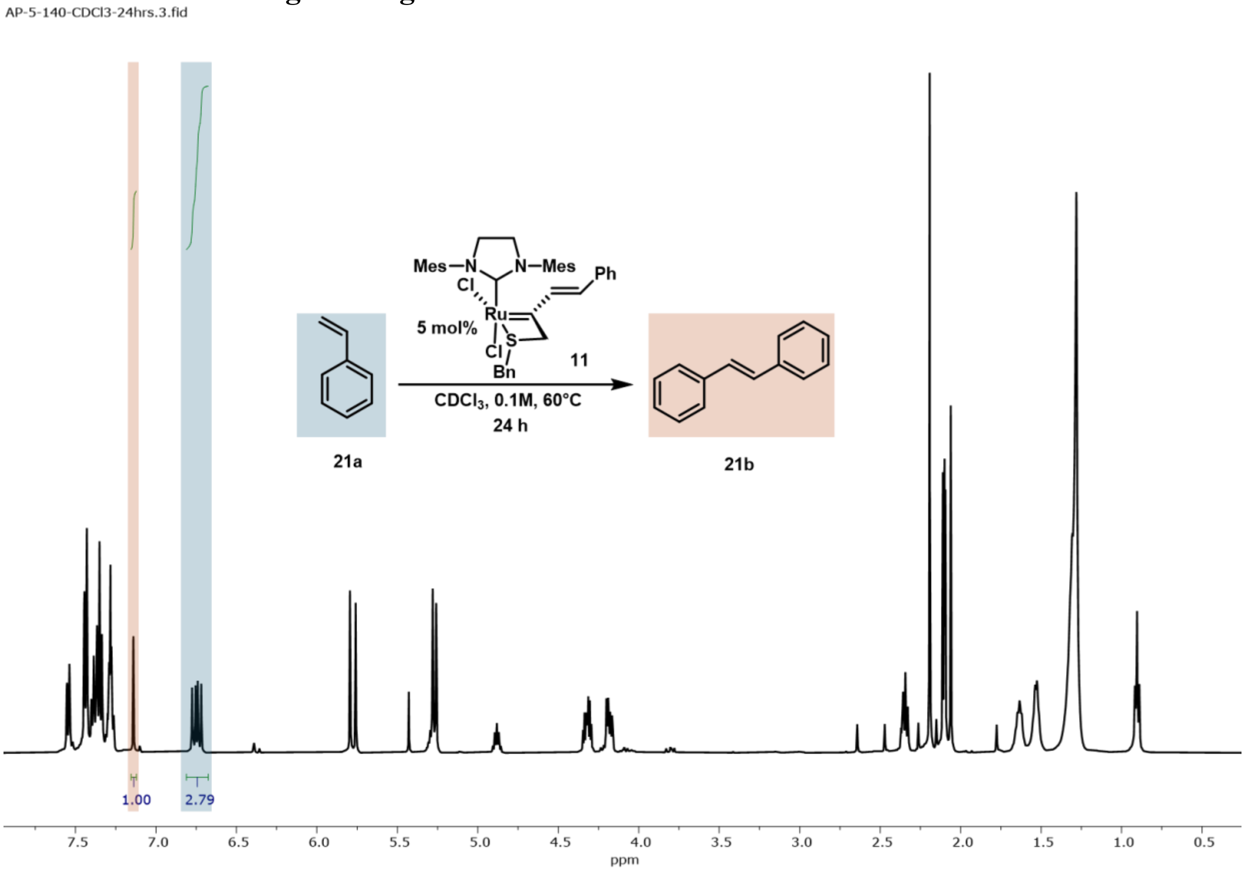

Figure S51. ^{1}H NMR of the RCM of **21a** in $CDCl_3$ at 60 °C for 24 h.

AP-5-145-CDCl3-24hrs.1.fid

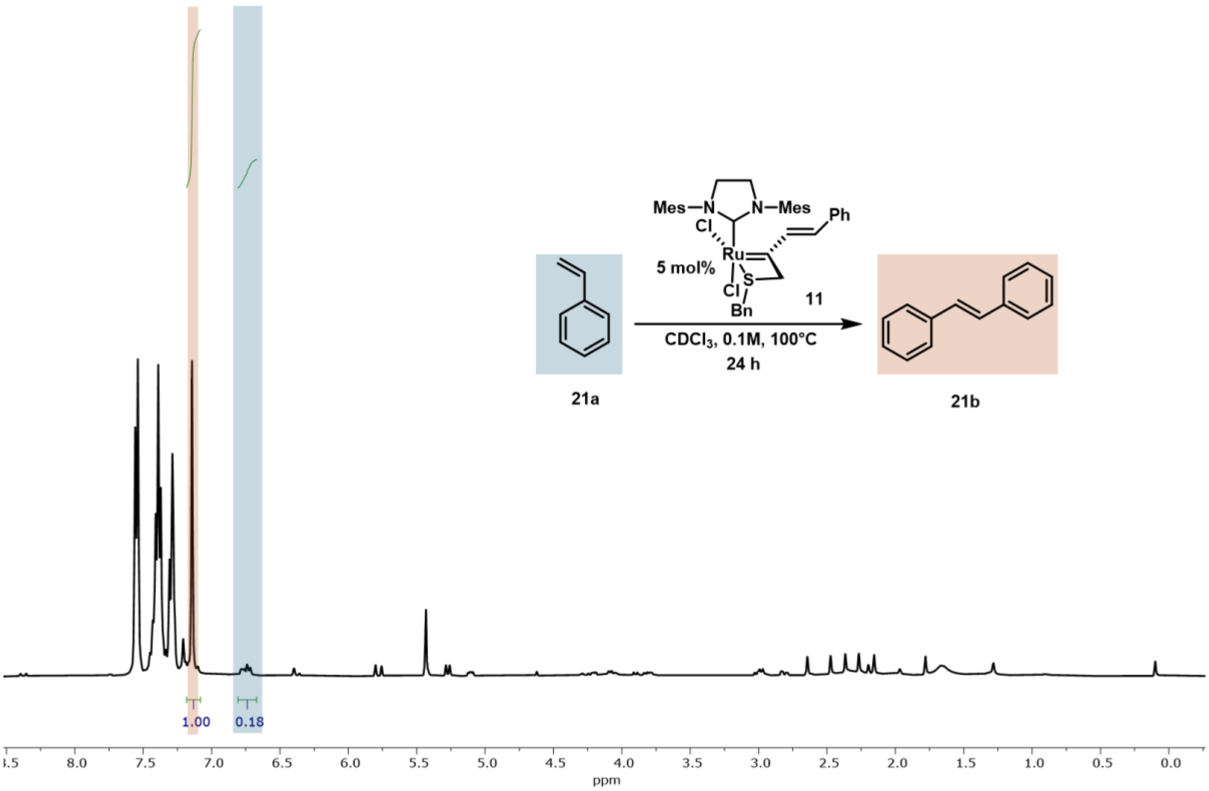

Figure S52. ^{1}H NMR of the RCM of **21a** in $CDCl_3$ at 100 °C for 24 h.

^{1}H NMRs of 22a Ring-Closing Metathesis Reactions

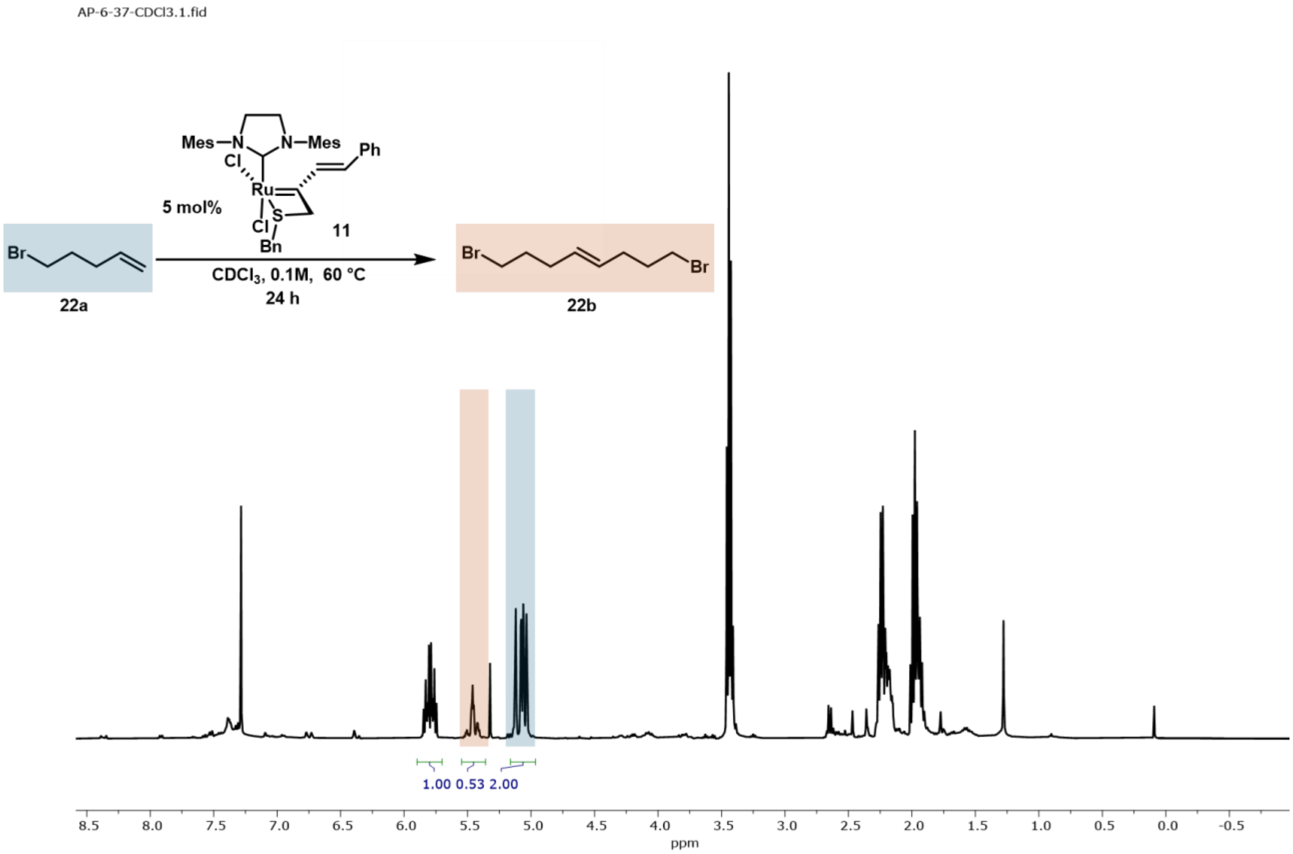

Figure S53. ^{1}H NMR of the RCM of **22a** in $CDCl_3$ at 60 °C for 24 h.

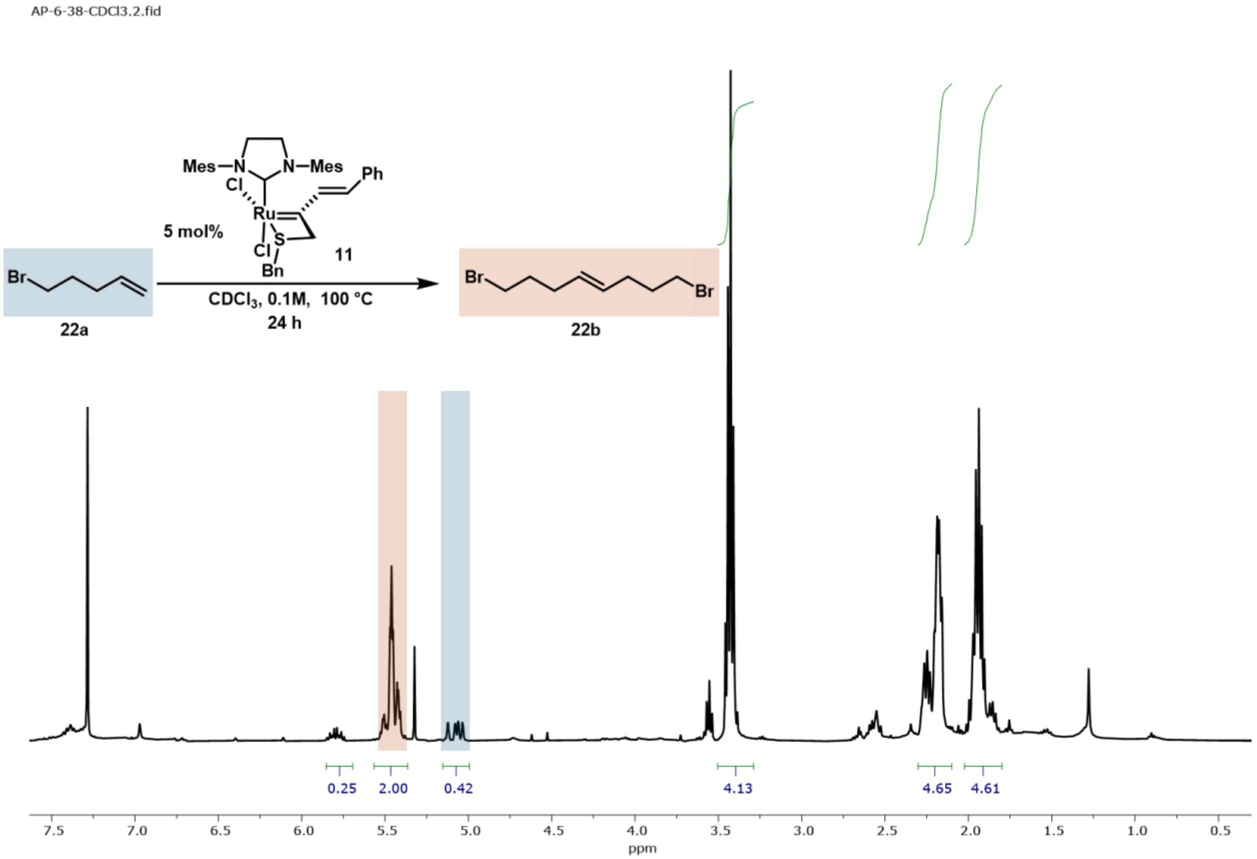

Figure S54. ^{1}H NMR of the RCM of **22a** in $CDCl_3$ at 100 °C for 24 h.

8. References

[1] L. Fu, T. Zhang, G. Fu, W. R. Gutekunst, *J. Am. Chem. Soc.* **2018**, *140*, 12181.

[2] T. Martzel, J.-F. Lohier, A.-C. Gaumont, J.-F. Brière, S. Perrio, *Eur. J. Org. Chem.* **2018**, *2018*, 5069.

[3] C. H. Kaschula, R. Hunter, N. Stellenboom, M. R. Caira, S. Winks, T. Ogunleye, P. Richards, J. Cotton, K. Zilbeyaz, Y. Wang, V. Siyo, E. Ngarande, M. I. Parker, *Eur. J. Med. Chem.* **2012**, *50*, 236.

[4] B. Schmidt, S. Krehl, E. Jablowski, *Org. Biomol. Chem.* **2012**, *10*, 5119.

[5] G. M. Sheldrick, *Acta Crystallogr. C* **2015**, *71*, 3.

[6] O. V Dolomanov, L. J. Bourhis, R. J. Gildea, J. A. K. Howard, H. Puschmann, *J. App.l Crystallogr.* **2009**, *42*, 339.

[7] L. J. Bourhis, O. V Dolomanov, R. J. Gildea, J. A. K. Howard, H. Puschmann, *Acta. Crystallogr. A* **2015**, *71*, 59.
